# Supplementary material for: Microbiotyping the Sinonasal Microbiome
Source: Front Cell Infect Microbiol. 2020 Apr 8;10:137. doi: 10.3389/fcimb.2020.00137 (PMC7156599; doi:10.3389/fcimb.2020.00137)
Supplement: Supplementary file 1 [file Data_Sheet_1.ZIP › international_microbiotypes_paper.html]

international\_microbiotypes\_paper\_revision


Notebook for the manuscript by Bassiouni et al.

# Load Experiments¶

In [1]:

```
%load_ext rpy2.ipython
%matplotlib inline

%load_ext autoreload
%autoreload 2

from matplotlib import pyplot
import numpy as np
import pandas as pd
import sklearn
import skbio
import sympy
from omicexperiment.experiment.microbiome import MicrobiomeExperiment
from omicexperiment.taxonomy import tax_as_dataframe
from omicexperiment.io.qiime2 import TaxonomyAssignment, BiomTable, Qiime2ArtifactFile, DistanceMatrix

from omicexperiment.transforms.observation import BinObservations, TopAbundantObservations, AbundancePrevalenceStatistics
from omicexperiment.transforms.sample import ExcludeSamples
from omicexperiment.transforms.taxonomy import TaxonomyGroupBy


#full OTU table
#counts_qza = "./results/deblur_tbl.qza"
counts_qza = "./results/filtered_tbl.qza"
counts_df = BiomTable(counts_qza).to_dataframe()

#rarefied OTU table
#counts_400_qza = "./results/counts_400.qza"
counts_400_qza = "./results/core-metrics-results/rarefied_table.qza"
counts_400_df = BiomTable(counts_400_qza).to_dataframe()

#metadata tsv file
metadata_tsv = "./data/int_microbiome_sample_metadata_23_11_2018_with_counts.tsv"

#taxonomy assignment (qiime2 blast assigner)
taxonomy_qza = "./results/blast_taxonomy.qza"
#extract taxonomy dataframe from the qiime2 taxonomy assignment
taxonomy_df = tax_as_dataframe(TaxonomyAssignment(taxonomy_qza).to_dataframe())

#rooted tree file (qiime 2 .qza file)
tree_qza = "./results/rooted_tree.qza"
#tree_qza = "./results/insertion_tree.qza"
#import the rooted tree qza into a tree node object
from omicexperiment.io.qiime2 import NewickTree
tree = NewickTree(tree_qza)
t = tree.to_treenode()


#Our MicrobiomeExperiment objects
exp_full = MicrobiomeExperiment(counts_df, metadata_tsv, taxonomy_df)
exp = MicrobiomeExperiment(counts_400_df, metadata_tsv, taxonomy_df)
exp_with_neg_ctrls = MicrobiomeExperiment(counts_400_df, metadata_tsv, taxonomy_df)

#set as 'dataset_main' in the metadata frame
exp_full.mapping_df['dataset'] = 'dataset_main'
exp.mapping_df['dataset'] = 'dataset_main'
exp_with_neg_ctrls.mapping_df['dataset'] = 'dataset_main'

#fastspar (SparCC) results - import to dataframes
fastspar_corr_df = pd.read_csv("./results/fastspar/correlation.tsv", sep="\t", index_col=0)
fastspar_pvalues_df = pd.read_csv("./results/fastspar/pvalues.tsv", sep="\t", index_col=0)

#results dataframe to import into our paper.md
results = pd.Series(name='value', index=pd.Index([], name='variable'), dtype='object')


#exclude neg control blank samples using the ExcludeSamples Transform
#################################################################################
control_samples = exp.mapping_df[exp.mapping_df.sample_type != "Microbiome swab"].index
exp = exp.apply(ExcludeSamples(control_samples))
exp.mapping_df = exp.mapping_df.reindex(exp.data_df.columns)

exp_full = exp_full.apply(ExcludeSamples(control_samples))
exp_full.mapping_df = exp_full.mapping_df.reindex(exp_full.data_df.columns)
#################################################################################


#genus level assignment
exp_genus = exp.apply(TaxonomyGroupBy("genus"))
```

##### Dataset 2 files¶

In [2]:

```
#full OTU table
dataset2_counts_qza = "./results/microbiotyping/dataset2_filtered_tbl.qza"
dataset2_counts_df = BiomTable(dataset2_counts_qza).to_dataframe()

#rarefied OTU table
dataset2_counts_400_qza = "./results/microbiotyping/core-metrics-results/rarefied_table.qza"
dataset2_counts_400_df = BiomTable(dataset2_counts_400_qza).to_dataframe()

#metadata tsv file
dataset2_metadata_tsv = "./data/2015_nasal_16s_2018_11_24.tsv"

#taxonomy assignment files
dataset2_taxonomy_qza = "./results/microbiotyping/dataset2_blast_taxonomy.qza"

#taxonomy dataframe
dataset2_taxonomy_df = tax_as_dataframe(TaxonomyAssignment(dataset2_taxonomy_qza).to_dataframe())

#rooted tree
dataset2_tree_qza = "./results/microbiotyping/dataset2_rooted_tree.qza"
#import the rooted tree qza into a tree node object
from omicexperiment.io.qiime2 import NewickTree
dataset2_tree = NewickTree(dataset2_tree_qza)
dataset2_t = tree.to_treenode()

#MicrobiomeExperiment objects
exp_full_dataset2 = MicrobiomeExperiment(dataset2_counts_df, dataset2_metadata_tsv, dataset2_taxonomy_df)
exp_dataset2 = MicrobiomeExperiment(dataset2_counts_400_df, dataset2_metadata_tsv, dataset2_taxonomy_df)

#set as dataset_two in the metadata frame
exp_full_dataset2.mapping_df['dataset'] = 'dataset_two'
exp_dataset2.mapping_df['dataset'] = 'dataset_two'


#sample with unknown classification "barotrauma : can't classify diseased vs non-diseased"
#for exclusion from the dataset 2 dataset
sample_unknown = ['5620-1377-ACAN3-GCTCATGA-GTAAGGAG-L001']
to_exclude = pd.Index(sample_unknown)

#exclude excluded samples#############
from omicexperiment.transforms.sample import ExcludeSamples
exp_full_dataset2 = exp_full_dataset2.apply(ExcludeSamples(to_exclude))
exp_full_dataset2.mapping_df = exp_full_dataset2.mapping_df.reindex(exp_full_dataset2.data_df.columns)

exp_dataset2 = exp_dataset2.apply(ExcludeSamples(to_exclude))
exp_dataset2.mapping_df = exp_dataset2.mapping_df.reindex(exp_dataset2.data_df.columns)

######################################

#genus level assignment
from omicexperiment.transforms.taxonomy import TaxonomyGroupBy
#exp_genus_full_dataset2 = exp_full_dataset2.apply(TaxonomyGroupBy("genus"))
exp_genus_dataset2 = exp_dataset2.apply(TaxonomyGroupBy("genus"))
```

In [3]:

```
results['paper'] = "microbiotyping"

results['sklearn_version'] = sklearn.__version__
results['skbio_version'] = skbio.__version__

results['n'] = len(exp.samples)
results['n_before_rarefaction'] = len(exp_full.samples)

results['n_Control'] = exp.mapping_df['diagnosis'].value_counts()['Control']
results['n_CRSsNP'] = exp.mapping_df['diagnosis'].value_counts()['CRSsNP']
results['n_CRSwNP'] = exp.mapping_df['diagnosis'].value_counts()['CRSwNP']
```

# Computing the Jensen-Shannon distance matrix¶

In [4]:

```
counts_df = exp_genus.to_relative_abundance().data_df

%load_ext rpy2.ipython
%R -i counts_df
%R library(philentropy)
%R jsd_dist <- as.matrix(JSD(t(counts_df/100),unit="log"))
#is equivalent to
#jsd_dist <- as.matrix(philentropy::distance(t(counts_df/100), "jensen-shannon"))
%R -o jsd_dist
jsd_dist = pd.DataFrame(jsd_dist, index=counts_df.columns, columns=counts_df.columns)
jsd_dist.head()
```

```
The rpy2.ipython extension is already loaded. To reload it, use:
  %reload_ext rpy2.ipython
```

```
/home/ahmed/dev/biomenv3/lib/python3.6/site-packages/rpy2/rinterface/__init__.py:185: RRuntimeWarning: Jensen-Shannon Divergence using unit 'log'.

  warnings.warn(x, RRuntimeWarning)
/home/ahmed/dev/biomenv3/lib/python3.6/site-packages/rpy2/rinterface/__init__.py:185: RRuntimeWarning: Metric: 'jensen-shannon' using unit: 'log'.

  warnings.warn(x, RRuntimeWarning)
```

Out[4]:

|  | AF18-16S-AR34D-TCGACGTC-GCGTAAGA | AX02-16S-B3RMH-ACTGAGCG-TCGACTAG | AK17-16S-AT526-GTAGAGGA-TCGACTAG | AK28-16S-AT526-AAGAGGCA-TTCTAGCT | AC08-16S-ATC1H-ACTGAGCG-ACTGCATA | AB09-16S-ATC1H-GCGTAGTA-TATCCTCT | AM08-16S-AT526-TAAGGCGA-AAGGCTAT | AB03-16S-ATC1H-CCTAAGAC-CTCTCTAT | AL10-16S-AT526-AAGAGGCA-CCTAGAGT | AQ21-16S-AT62V-TCGACGTC-CTATTAAG | ... | AG25-16S-AR34D-ACTCGCTA-TTATGCGA | AS09-16S-B39FG-GCTCATGA-CTCTCTAT | AB21-16S-ATC1H-GCGTAGTA-GTAAGGAG | AL27-16S-AT526-CGTACTAG-CTATTAAG | AW25-16S-B39FG-TAGGCATG-TCTCTCCG | AQ17-16S-AT62V-ACTGAGCG-CTATTAAG | AA14-16S-ARW7H-GTAGAGGA-GAGCCTTA | AB02-16S-ATC1H-ACTGAGCG-CTCTCTAT | AE06-16S-AR34D-TAGCGCTC-TCGACTAG | AL11-16S-AT526-GTAGAGGA-CCTAGAGT |
| --- | --- | --- | --- | --- | --- | --- | --- | --- | --- | --- | --- | --- | --- | --- | --- | --- | --- | --- | --- | --- | --- |
| AF18-16S-AR34D-TCGACGTC-GCGTAAGA | 0.000000 | 0.352815 | 0.621775 | 0.047359 | 0.478221 | 0.478221 | 0.052122 | 0.162747 | 0.490729 | 0.483222 | ... | 0.120272 | 0.678259 | 0.175925 | 0.061516 | 0.043533 | 0.693147 | 0.566617 | 0.059070 | 0.686832 | 0.496976 |
| AX02-16S-B3RMH-ACTGAGCG-TCGACTAG | 0.352815 | 0.000000 | 0.561892 | 0.539052 | 0.072859 | 0.072859 | 0.547324 | 0.427555 | 0.223163 | 0.087240 | ... | 0.117261 | 0.667639 | 0.299866 | 0.530212 | 0.528643 | 0.693147 | 0.560160 | 0.348893 | 0.684677 | 0.153801 |
| AK17-16S-AT526-GTAGAGGA-TCGACTAG | 0.621775 | 0.561892 | 0.000000 | 0.687585 | 0.553831 | 0.553831 | 0.693147 | 0.638572 | 0.545124 | 0.556636 | ... | 0.577022 | 0.680464 | 0.604573 | 0.661194 | 0.683716 | 0.693147 | 0.649302 | 0.624590 | 0.687585 | 0.564383 |
| AK28-16S-AT526-AAGAGGCA-TTCTAGCT | 0.047359 | 0.539052 | 0.687585 | 0.000000 | 0.684406 | 0.684406 | 0.001736 | 0.196207 | 0.657144 | 0.684500 | ... | 0.261045 | 0.690336 | 0.271361 | 0.012210 | 0.001946 | 0.693147 | 0.615950 | 0.108004 | 0.691414 | 0.679463 |
| AC08-16S-ATC1H-ACTGAGCG-ACTGCATA | 0.478221 | 0.072859 | 0.553831 | 0.684406 | 0.000000 | 0.000000 | 0.693147 | 0.550559 | 0.192996 | 0.025808 | ... | 0.198251 | 0.671035 | 0.386477 | 0.693147 | 0.677395 | 0.693147 | 0.588863 | 0.491480 | 0.684406 | 0.094545 |

5 rows × 410 columns

## Performing PCoA on the Jensen-Shannon and Weighted-Unifrac distance matrices¶

In [5]:

```
from omicexperiment.transforms.transform import Transform
from omicexperiment.transforms.diversity import BetaDiversity, AlphaDiversity
from scipy.spatial.distance import cdist
from functools import partial

from skbio.diversity import beta_diversity, alpha_diversity
from skbio.diversity.alpha import faith_pd

from omicexperiment.transforms.ordination import pcoa, PCoA


beta_div_exp = exp_genus.with_data_df(jsd_dist)
dist_matrix_pcoa = beta_div_exp.apply(PCoA)

pcoa_obj = dist_matrix_pcoa.metadata['pcoa']

print("JSD PCs: Proportions explained")
display(pcoa_obj.proportion_explained.head())

PC1_proportion_explained = round(pcoa_obj.proportion_explained['PC1'] * 100, 2)
PC2_proportion_explained = round(pcoa_obj.proportion_explained['PC2'] * 100, 2)
PC3_proportion_explained = round(pcoa_obj.proportion_explained['PC3'] * 100, 2)
results['PC1_proportion_explained'] = PC1_proportion_explained
results['PC2_proportion_explained'] = PC2_proportion_explained
results['PC3_proportion_explained'] = PC3_proportion_explained


weighted_unifrac_dm = DistanceMatrix('./results/core-metrics-results/weighted_unifrac_distance_matrix.qza')
weighted_unifrac_dm_df = weighted_unifrac_dm.to_dataframe()
weighted_unifrac = exp.with_data_df(weighted_unifrac_dm_df)
weighted_unifrac_pcoa = weighted_unifrac.apply(PCoA)

weighted_unifrac_pcoa_obj = weighted_unifrac_pcoa.metadata['pcoa']

print("Weighted Unifrac PCs: Proportions explained")
display(weighted_unifrac_pcoa_obj.proportion_explained.head())


weighted_unifrac_PC1_proportion_explained = round(weighted_unifrac_pcoa_obj.proportion_explained['PC1'] * 100, 2)
weighted_unifrac_PC2_proportion_explained = round(weighted_unifrac_pcoa_obj.proportion_explained['PC2'] * 100, 2)
weighted_unifrac_PC3_proportion_explained = round(weighted_unifrac_pcoa_obj.proportion_explained['PC3'] * 100, 2)

results['weighted_unifrac_PC1_proportion_explained'] = weighted_unifrac_PC1_proportion_explained
results['weighted_unifrac_PC2_proportion_explained'] = weighted_unifrac_PC2_proportion_explained
results['weighted_unifrac_PC3_proportion_explained'] = weighted_unifrac_PC3_proportion_explained
```

```
/home/ahmed/dev/biomenv3/lib/python3.6/site-packages/skbio/stats/ordination/_principal_coordinate_analysis.py:111: RuntimeWarning: The result contains negative eigenvalues. Please compare their magnitude with the magnitude of some of the largest positive eigenvalues. If the negative ones are smaller, it's probably safe to ignore them, but if they are large in magnitude, the results won't be useful. See the Notes section for more details. The smallest eigenvalue is -3.206381729895014 and the largest is 20.240781626448637.
  RuntimeWarning
```

```
JSD PCs: Proportions explained
```

```
PC1    0.366676
PC2    0.219273
PC3    0.091606
PC4    0.073005
PC5    0.053909
dtype: float64
```

```
Weighted Unifrac PCs: Proportions explained
```

```
/home/ahmed/dev/biomenv3/lib/python3.6/site-packages/skbio/stats/ordination/_principal_coordinate_analysis.py:111: RuntimeWarning: The result contains negative eigenvalues. Please compare their magnitude with the magnitude of some of the largest positive eigenvalues. If the negative ones are smaller, it's probably safe to ignore them, but if they are large in magnitude, the results won't be useful. See the Notes section for more details. The smallest eigenvalue is -1.0806168375284075 and the largest is 28.529795930827508.
  RuntimeWarning
```

```
PC1    0.401561
PC2    0.256718
PC3    0.068330
PC4    0.047572
PC5    0.037043
dtype: float64
```

In [6]:

```
joined_df = dist_matrix_pcoa.data_df.T.join(dist_matrix_pcoa.mapping_df)
joined_df = joined_df[joined_df['PC1'].notnull()]
three_PCs_df = dist_matrix_pcoa.data_df.T[['PC1', 'PC2', 'PC3']]

weighted_unifrac_joined_df = weighted_unifrac_pcoa.data_df.T.join(weighted_unifrac_pcoa.mapping_df)
weighted_unifrac_joined_df = weighted_unifrac_joined_df[weighted_unifrac_joined_df['PC1'].notnull()]
weighted_unifrac_three_PCs_df = weighted_unifrac_pcoa.data_df.T[['PC1', 'PC2', 'PC3']]
```

# PCoA Plots¶

In [7]:

```
%matplotlib inline

from matplotlib import pyplot
import matplotlib.pyplot as pyplt
from matplotlib.colors import to_hex

cmap_tab20 = pyplot.get_cmap('tab20')
colours_all = [to_hex(c) for c in cmap_tab20.colors]
colours = [to_hex(c) for c in cmap_tab20.colors[::2]]


fig_pcoa = pyplot.figure(figsize=(14, 14))
grid_fig_pcoa = pyplot.GridSpec(2, 2, wspace=0.2, hspace=0.2)


diagnoses = ['Control', 'CRSsNP', 'CRSwNP']
centres = exp_genus.mapping_df['centre'].sort_values().unique()


ax1 = fig_pcoa.add_subplot(grid_fig_pcoa[0,0])

scatters = []
for i, diagnosis in enumerate(diagnoses):
    subsetted_df = weighted_unifrac_joined_df[weighted_unifrac_joined_df['diagnosis'] == diagnosis]
    scatter = ax1.scatter(subsetted_df['PC1'], subsetted_df['PC2'], label=diagnosis, color=colours[i])
    scatters.append(scatter)
    
ax1.set_xlabel("PC1 ({}%)".format(weighted_unifrac_PC1_proportion_explained))
ax1.set_ylabel("PC2 ({}%)".format(weighted_unifrac_PC2_proportion_explained))
ax1.set_title("Weighted UniFrac PCoA - by Diagnosis")
ax1.legend(scatters, diagnoses)

pyplot.figtext(0.10,0.90, "A.", fontsize=16, axes=ax1)

ax2 = fig_pcoa.add_subplot(grid_fig_pcoa[1,0])

scatters = []
for i, centre in enumerate(centres):
    subsetted_df = weighted_unifrac_joined_df[weighted_unifrac_joined_df['centre'] == centre]
    scatter = ax2.scatter(subsetted_df['PC1'], subsetted_df['PC2'], label=centre, color=colours_all[i])
    scatters.append(scatter)

ax2.set_xlabel("PC1 ({}%)".format(weighted_unifrac_PC1_proportion_explained))
ax2.set_ylabel("PC2 ({}%)".format(weighted_unifrac_PC2_proportion_explained))
ax2.set_title("Weighted UniFrac PCoA - by Centre")
ax2.legend(scatters, centres, loc='upper right')

pyplot.figtext(0.50,0.90, "C.", fontsize=16, axes=ax2)

ax3 = fig_pcoa.add_subplot(grid_fig_pcoa[0,1])

scatters = []
for i, diagnosis in enumerate(diagnoses):
    subsetted_df = joined_df[joined_df['diagnosis'] == diagnosis]
    scatter = ax3.scatter(subsetted_df['PC1'], subsetted_df['PC2'], label=diagnosis, color=colours[i])
    scatters.append(scatter)
    
ax3.set_xlabel("PC1 ({}%)".format(PC1_proportion_explained))
ax3.set_ylabel("PC2 ({}%)".format(PC2_proportion_explained))
ax3.set_title("Jensen-Shannon PCoA - by Diagnosis")
ax3.legend(scatters, diagnoses)


pyplot.figtext(0.10,0.49, "B.", fontsize=16, axes=ax3)

ax4 = fig_pcoa.add_subplot(grid_fig_pcoa[1,1])

scatters = []
for i, centre in enumerate(centres):
    subsetted_df = joined_df[joined_df['centre'] == centre]
    scatter = ax4.scatter(subsetted_df['PC1'], subsetted_df['PC2'], label=centre, color=colours_all[i])
    scatters.append(scatter)

ax4.set_xlabel("PC1 ({}%)".format(PC1_proportion_explained))
ax4.set_ylabel("PC2 ({}%)".format(PC2_proportion_explained))
ax4.set_title("Jensen-Shannon PCoA - by Centre")
ax4.legend(scatters, centres, loc='lower right')

pyplot.figtext(0.50,0.49, "D.", fontsize=16, axes=ax3)

fig_pcoa.savefig("./results/microbiotyping/figures/beta_div_jsd_and_unifrac_diagnosis_centre.jpg", bbox_inches='tight', dpi=300)
```

# K-Means Clustering¶

We use K-Means Clustering algorithm, as implemented in *scikit-learn*, on the first two Principal Components from the PCoA above.

We decided to set n\_clusters = 3, based on review of the above plots, which show a "triangular" distribution.

In [8]:

```
from sklearn.cluster import KMeans

clusterer = kmeans = kmeans_main = KMeans(n_clusters=3, random_state=0).fit(three_PCs_df[['PC1', 'PC2']])

clusters = pd.Series(["cluster_" + str(x + 1) for x in clusterer.labels_], index=three_PCs_df.index)
microbiotypes = pd.Series(["microbiotype_" + str(x + 1) for x in clusterer.labels_], index=three_PCs_df.index)

joined_df['cluster'] = clusters
exp_genus.mapping_df['cluster'] = clusters

#Name the clusters or "microbiotypes"
cluster_coryne_max = exp_genus.groupby("cluster").to_relative_abundance().data_df.T['g__Corynebacterium'].sort_values().index[-1]
cluster_staph_max = exp_genus.groupby("cluster").to_relative_abundance().data_df.T['g__Staphylococcus'].sort_values().index[-1]
cluster_haemophilus_max = exp_genus.groupby("cluster").to_relative_abundance().data_df.T['g__Haemophilus'].sort_values().index[-1]

microbiotype_rename_dict = {cluster_coryne_max: 'microbiotype_1',
                           cluster_staph_max: 'microbiotype_2',
                           cluster_haemophilus_max: 'microbiotype_3'}

microbiotypes = exp_genus.mapping_df['cluster'].replace(microbiotype_rename_dict)
joined_df['microbiotype'] = microbiotypes
exp_genus.mapping_df['microbiotype'] = microbiotypes
```

In [9]:

```
from sklearn.metrics import silhouette_samples, silhouette_score

range_n_clusters = [2, 3, 4, 5, 6, 7, 8]

s_scores = {}

for n_clusters in range_n_clusters:
    # Initialize the clusterer with n_clusters value and a random generator
    # seed of 10 for reproducibility.
    silhouette_clusterer = KMeans(n_clusters=n_clusters)
    silhouette_cluster_labels = silhouette_clusterer.fit_predict(three_PCs_df[['PC1', 'PC2']])

    # The silhouette_score gives the average value for all the samples.
    # This gives a perspective into the density and separation of the formed
    # clusters
    silhouette_avg = silhouette_score(three_PCs_df[['PC1', 'PC2']], silhouette_cluster_labels)
    print("For n_clusters =", n_clusters,
          "The average silhouette_score is :", silhouette_avg)
    
    s_scores[n_clusters] = silhouette_avg

pyplot.plot([n for n, s in s_scores.items()], [s for n, s in s_scores.items()])
pyplot.ylim(0.485, 0.7)
pyplot.xlabel("n")
pyplot.ylabel("Silhouette score")
```

```
For n_clusters = 2 The average silhouette_score is : 0.489419645415
For n_clusters = 3 The average silhouette_score is : 0.598766739279
For n_clusters = 4 The average silhouette_score is : 0.606981167827
For n_clusters = 5 The average silhouette_score is : 0.572312652074
For n_clusters = 6 The average silhouette_score is : 0.558887727927
For n_clusters = 7 The average silhouette_score is : 0.57394705021
For n_clusters = 8 The average silhouette_score is : 0.544394516983
```

Out[9]:

```
Text(0,0.5,'Silhouette score')
```

In [10]:

```
results['silhouette_score_k_3'] = round(s_scores[3], 2)
results['silhouette_score_k_4'] = round(s_scores[4], 2)
```

A high Silhouette score indicates a model with better clustering.  
Here k=4 and k=3 have the highest silhouette scores.

In [11]:

```
###########################################
#Essential plotting functions
###########################################
from matplotlib import pyplot
from omicexperiment.transforms.observation import AbundancePrevalenceStatistics

def top_abundant_taxa(exp, n):
    from omicexperiment.transforms.observation import AbundancePrevalenceStatistics
    
    abund_prev_df = exp.apply(AbundancePrevalenceStatistics()).data_df
    abund_series = abund_prev_df['mean_relative_abundance']
    
    top_taxa = list(abund_series.head(n).index)
    
    return top_taxa


def return_cumsum_df(exp, top_taxa=10):
    flipped_df = exp.data_df.iloc[::-1]
    flipped_index = flipped_df.index
    flipped_columns = flipped_df.columns
    
    cumsum_df = pd.DataFrame(np.cumsum(flipped_df.as_matrix(), axis=0),
                             index=flipped_index,
                             columns=flipped_columns).iloc[::-1]
        
    return cumsum_df

def taxa_bar_plot(exp, top_taxa=10):
    data_df = exp.data_df
    cumsum_df = return_cumsum_df(exp, top_taxa)
    fig = pyplot.figure(figsize=(10, 6))
    grid = pyplot.GridSpec(1, 2, wspace=0.2, hspace=0.1)
    main_ax = fig.add_subplot(grid[0,0])
    main_ax.set_ylim(0,100)
    main_ax.set_ylabel("Relative Abundance (%)")
    bars = []
    for i, arr in enumerate(cumsum_df.as_matrix()):
        bar_ = main_ax.bar(cumsum_df.columns, arr)
        bars.append(bar_[0])
        
    ax_leg = fig.add_subplot(grid[0, 1])
    pyplot.axis('off')
    ax_leg.legend(bars,list(cumsum_df.index), loc='center left')

    return fig


from omicexperiment.transforms.observation import BinObservations, TopAbundantObservations

exp_genus_microbiotypes = exp_genus.to_relative_abundance().groupby('microbiotype')

genus_microbiotypes_df = exp_genus_microbiotypes.data_df
genus_microbiotypes_cumsum_df = return_cumsum_df(exp_genus_microbiotypes)

top_taxa = top_abundant_taxa(exp_genus, 10)
other_taxa = list(genus_microbiotypes_cumsum_df.index.difference(top_taxa))

exp_genus_microbiotypes_other = exp_genus_microbiotypes.apply(BinObservations(other_taxa))
exp_genus_other = exp_genus.to_relative_abundance().apply(BinObservations(other_taxa))

taxa_not_other = [taxon for taxon in exp_genus_other.apply(AbundancePrevalenceStatistics()).data_df.index
                  if taxon != 'Other']

taxa_plus_other = ['Other'] + sorted(taxa_not_other) #sorted alphabetically here - will be used for plot legends

exp_genus_other_microbiotype1 = exp_genus_other[exp_genus_other.Sample.c.microbiotype=='microbiotype_1']
exp_genus_other_microbiotype2 = exp_genus_other[exp_genus_other.Sample.c.microbiotype=='microbiotype_2']
exp_genus_other_microbiotype3 = exp_genus_other[exp_genus_other.Sample.c.microbiotype=='microbiotype_3']

results['mra_coryne_microbiotype_1'] = round(exp_genus_microbiotypes.data_df.T['g__Corynebacterium']['microbiotype_1'], 2)
results['mra_staph_microbiotype_2'] = round(exp_genus_microbiotypes.data_df.T['g__Staphylococcus']['microbiotype_2'], 2)
```

# The taxonomic composition of the three Microbiotypes¶

In [12]:

```
#functions to clean taxa names from Greengenes

def clean_taxon_name(taxon):
    return \
        taxon.replace("_f__", " ")\
             .replace("g__unidentified", "unidentified") \
             .replace("g__", "") \
             .replace("f__", "") \
    

def clean_taxa_in_index(dataframe):
    return dataframe.set_index(pd.Index([clean_taxon_name(x) for x in dataframe.index],
                                       name=dataframe.index.name))
```

##### Genus-level assignment¶

In [13]:

```
fig = exp_genus.apply(TopAbundantObservations(10)).to_relative_abundance().groupby('microbiotype').plot(backend='matplotlib')
```

In [14]:

```
from omicexperiment.transforms.observation import AbundancePrevalenceStatistics
abund_prev_df_microbiotype1 = exp_genus[exp_genus.Sample.c.microbiotype =='microbiotype_1'].apply(AbundancePrevalenceStatistics()).data_df.head(10)
abund_prev_df_microbiotype2 = exp_genus[exp_genus.Sample.c.microbiotype =='microbiotype_2'].apply(AbundancePrevalenceStatistics()).data_df.head(10)
abund_prev_df_microbiotype3 = exp_genus[exp_genus.Sample.c.microbiotype =='microbiotype_3'].apply(AbundancePrevalenceStatistics()).data_df.head(10)

#abund_prev_df_microbiotype1.to_csv("./results/microbiotyping/tables/SUPP_abund_prev_microbiotype_1", sep="\t")
#abund_prev_df_microbiotype2.to_csv("./results/microbiotyping/tables/SUPP_abund_prev_microbiotype_2", sep="\t")
#abund_prev_df_microbiotype3.to_csv("./results/microbiotyping/tables/SUPP_abund_prev_microbiotype_3", sep="\t")
```

In [15]:

```
print("Genus-level Composition of Microbiotype 1:")
abund_prev_df_microbiotype1.rename({"mean_relative_abundance": "Mean Relative Abundance (%)",
                                      "prevalence": "Prevalence (%)"}, axis=1, inplace=True)
abund_prev_df_microbiotype1 = abund_prev_df_microbiotype1.apply(round, args=(2,))
display(clean_taxa_in_index(abund_prev_df_microbiotype1))
clean_taxa_in_index(abund_prev_df_microbiotype1).to_csv("./results/microbiotyping/tables/SUPP_microbiotype1.tsv", sep="\t")

print("Genus-level Composition of Microbiotype 2:")
abund_prev_df_microbiotype2 = abund_prev_df_microbiotype2.apply(round, args=(2,))
abund_prev_df_microbiotype2.rename({"mean_relative_abundance": "Mean Relative Abundance (%)",
                                      "prevalence": "Prevalence (%)"}, axis=1, inplace=True)
display(clean_taxa_in_index(abund_prev_df_microbiotype2))
clean_taxa_in_index(abund_prev_df_microbiotype2).to_csv("./results/microbiotyping/tables/SUPP_microbiotype2.tsv", sep="\t")

print("Genus-level Composition of Microbiotype 3:")
abund_prev_df_microbiotype3 = abund_prev_df_microbiotype3.apply(round, args=(2,))
abund_prev_df_microbiotype3.rename({"mean_relative_abundance": "Mean Relative Abundance (%)",
                                      "prevalence": "Prevalence (%)"}, axis=1, inplace=True)
display(clean_taxa_in_index(abund_prev_df_microbiotype3))
clean_taxa_in_index(abund_prev_df_microbiotype3).to_csv("./results/microbiotyping/tables/SUPP_microbiotype3.tsv", sep="\t")
```

```
Genus-level Composition of Microbiotype 1:
```

|  | Mean Relative Abundance (%) | Prevalence (%) |
| --- | --- | --- |
| genus |  |  |
| Corynebacterium | 75.29 | 100.00 |
| Staphylococcus | 10.69 | 76.58 |
| Alloiococcus | 2.79 | 28.83 |
| Moraxella | 2.31 | 9.91 |
| unidentified (Enterobacteriaceae) | 1.41 | 15.32 |
| unidentified (Neisseriaceae) | 1.18 | 20.72 |
| Streptococcus | 1.00 | 21.62 |
| Haemophilus | 0.56 | 9.91 |
| unidentified (Moraxellaceae) | 0.44 | 2.70 |
| Ralstonia | 0.34 | 10.36 |

```
Genus-level Composition of Microbiotype 2:
```

|  | Mean Relative Abundance (%) | Prevalence (%) |
| --- | --- | --- |
| genus |  |  |
| Staphylococcus | 74.96 | 100.00 |
| Corynebacterium | 9.87 | 64.10 |
| Streptococcus | 3.22 | 25.64 |
| unidentified (Enterobacteriaceae) | 1.82 | 15.38 |
| Haemophilus | 1.41 | 10.26 |
| Moraxella | 1.27 | 5.13 |
| Ralstonia | 1.19 | 11.97 |
| Pseudomonas | 1.05 | 6.84 |
| Parvimonas | 0.72 | 0.85 |
| unidentified (Neisseriaceae) | 0.61 | 7.69 |

```
Genus-level Composition of Microbiotype 3:
```

|  | Mean Relative Abundance (%) | Prevalence (%) |
| --- | --- | --- |
| genus |  |  |
| Haemophilus | 23.78 | 40.85 |
| Streptococcus | 23.22 | 46.48 |
| Moraxella | 12.11 | 19.72 |
| Pseudomonas | 9.17 | 15.49 |
| unidentified (Enterobacteriaceae) | 5.74 | 9.86 |
| Serratia | 5.70 | 8.45 |
| Klebsiella | 2.75 | 4.23 |
| Corynebacterium | 2.56 | 46.48 |
| Prevotella | 1.44 | 12.68 |
| Acinetobacter | 1.38 | 1.41 |

### Differential abundance of genera between the three microbiotypes¶

Here we used ANCOM (Mandal et al.), which accounts for the compositional nature of the data, in the implemention in scikit-bio.  
ANCOM here is applied after application of "multiplicative replacement" to replace zeros in the data.

In [16]:

```
from skbio.stats.composition import ancom, multiplicative_replacement
mult_rep_df = pd.DataFrame(multiplicative_replacement(exp_genus.data_df), index=exp_genus.data_df.index, columns=exp_genus.data_df.columns)

ancom_results = ancom(mult_rep_df.T, exp_genus.mapping_df.reindex(exp_genus.data_df.T.index)['microbiotype'])
ancom_results[0][ancom_results[0]['Reject null hypothesis'] == True]
```

```
/home/ahmed/dev/biomenv3/lib/python3.6/site-packages/scipy/stats/stats.py:2926: RuntimeWarning: invalid value encountered in double_scalars
  f = msb / msw
```

Out[16]:

|  | Reject null hypothesis | W |
| --- | --- | --- |
| genus |  |  |
| g\_\_Alloiococcus | True | 191 |
| g\_\_Corynebacterium | True | 192 |
| g\_\_Haemophilus | True | 191 |
| g\_\_Staphylococcus | True | 192 |
| g\_\_Streptococcus | True | 191 |

### Biplot of features¶

In [17]:

```
#Here we calculate the biplot using the *genera* as features
from skbio.stats.ordination import pcoa_biplot
biplot_genus_jsd = pcoa_biplot(pcoa_obj, exp_genus.data_df.T)
biplot_jsd_genus_feature_PCs_df = biplot_genus_jsd.features[['PC1', 'PC2']]
biplot_jsd_genus_feature_PCs_df = biplot_jsd_genus_feature_PCs_df.reindex(taxa_not_other)
biplot_jsd_genus_feature_PCs_df
```

Out[17]:

|  | PC1 | PC2 |
| --- | --- | --- |
| genus |  |  |
| g\_\_Corynebacterium | 603.370986 | 322.044038 |
| g\_\_Staphylococcus | -491.607317 | 439.522591 |
| g\_\_Streptococcus | -44.521385 | -222.194062 |
| g\_\_Haemophilus | -41.408488 | -241.116937 |
| g\_\_Moraxella | 2.055630 | -96.655538 |
| g\_\_unidentified (f\_\_Enterobacteriaceae) | -6.243906 | -25.583841 |
| g\_\_Pseudomonas | -16.872451 | -54.851540 |
| g\_\_Alloiococcus | 27.593659 | -5.443619 |
| g\_\_Serratia | -7.583216 | -32.805125 |
| g\_\_unidentified (f\_\_Neisseriaceae) | 3.525380 | 7.850499 |

In [18]:

```
taxa_not_other = [taxon for taxon in 
                  exp_genus_other.apply(AbundancePrevalenceStatistics()).data_df.index
                  if taxon != 'Other']

taxa_plus_other = ['Other'] + sorted(taxa_not_other)

#set colours for the taxa
cmap_tab10 = pyplot.get_cmap('tab10')
from matplotlib.colors import to_hex
colours_tab10_11 = [to_hex(c) for c in (list(cmap_tab10.colors) + [cmap_tab20.colors[0]])]

taxa_colours = {tax: col for tax, col in zip(taxa_plus_other, colours_tab10_11)}

from matplotlib.patches import Circle
from matplotlib.patheffects import withStroke

#calculations required for biplot
####################################
pcoa_plot_centre = joined_df[['PC1', 'PC2']].apply(lambda x: x.min() + ((np.sqrt((x.max() - x.min())**2) / 2)))
pcoa_plot_centre = pcoa_plot_centre['PC1'], pcoa_plot_centre['PC2']
pcoa_plot_centre = sympy.Rational(str(pcoa_plot_centre[0])), sympy.Rational(str(pcoa_plot_centre[1]))

pcoa_plot_range = joined_df[['PC1', 'PC2']].apply(lambda x: np.sqrt((x.max() - x.min())**2))
pcoa_plot_range = pcoa_plot_range['PC1'], pcoa_plot_range['PC2']
sympy_circle_radius = sympy.Rational(str(max(pcoa_plot_range) / 2))
sympy_circle = sympy.Circle(pcoa_plot_centre, sympy_circle_radius)


fig = pyplot.figure(figsize=(10, 10))
grid = pyplot.GridSpec(1, 1, wspace=0.6, hspace=0.3)
ax = fig.add_subplot(grid[0,0])

for taxon in taxa_not_other[:5]:
    print(taxon)
    pyplot.scatter(biplot_jsd_genus_feature_PCs_df['PC1'][taxon], biplot_jsd_genus_feature_PCs_df['PC2'][taxon],
                   c=taxa_colours[taxon], s=80, marker='X')
    circle = Circle(pcoa_plot_centre, min(pcoa_plot_range), clip_on=False, zorder=10, linewidth=1,
                    alpha=0.03,
                    edgecolor='black', facecolor='white',
                    path_effects=[withStroke(linewidth=5, foreground='w')])
    ax.add_artist(circle)

pyplot.legend(list(taxa_not_other[:5]))
```

```
g__Corynebacterium
g__Staphylococcus
g__Streptococcus
g__Haemophilus
g__Moraxella
```

Out[18]:

```
<matplotlib.legend.Legend at 0x7fa43ef147b8>
```

In [19]:

```
import matplotlib.pyplot as plt
import matplotlib.gridspec as gridspec

#set figure and grid
fig = pyplot.figure(figsize=(10, 10))
grid = pyplot.GridSpec(1, 1, wspace=0.6, hspace=0.3)

#set colours
cmap_tab10 = pyplot.get_cmap('tab10')
colours_tab10_11 = list(cmap_tab10.colors) + [cmap_tab20.colors[0]]
from matplotlib.colors import to_hex
colours_tab10_11 = [to_hex(c) for c in colours]

colours_microbiotypes = ['royalblue', 'orange', 'indianred']


#microbiotyping scatter
####################################
ax12 = fig.add_subplot(grid[0,0])

taxa_not_other = [taxon for taxon in 
                  exp_genus_other.apply(AbundancePrevalenceStatistics()).data_df.index
                  if taxon != 'Other']

scatters = []
for i, cluster in enumerate(['microbiotype_1', 'microbiotype_2', 'microbiotype_3']):
    scatter = ax12.scatter(joined_df[joined_df['microbiotype']==cluster]['PC1'],
                   joined_df[joined_df['microbiotype']==cluster]['PC2'],
                   c=colours_microbiotypes[i])
    scatters.append(scatter)

ax12.legend(scatters)

#calculations required for biplot
####################################
pcoa_plot_centre = joined_df[['PC1', 'PC2']].apply(lambda x: x.min() + ((np.sqrt((x.max() - x.min())**2) / 2)))
pcoa_plot_centre = pcoa_plot_centre['PC1'], pcoa_plot_centre['PC2']
pcoa_plot_centre = sympy.Rational(str(pcoa_plot_centre[0])), sympy.Rational(str(pcoa_plot_centre[1]))

pcoa_plot_range = joined_df[['PC1', 'PC2']].apply(lambda x: np.sqrt((x.max() - x.min())**2))
pcoa_plot_range = pcoa_plot_range['PC1'], pcoa_plot_range['PC2']
sympy_circle_radius = sympy.Rational(str(max(pcoa_plot_range) / 2))
sympy_circle = sympy.Circle(pcoa_plot_centre, sympy_circle_radius)

#here we choose top five taxa
top_taxa_for_biplot = taxa_not_other[:5]

#calculate longest of the taxa lines - required for scaling the biplot feature arrows
taxa_lines_lengths = []

for taxon in top_taxa_for_biplot:
    taxon_biplot_PCs = biplot_jsd_genus_feature_PCs_df['PC1'][taxon], biplot_jsd_genus_feature_PCs_df['PC2'][taxon]
    taxon_line = sympy.Segment2D(pcoa_plot_centre, taxon_biplot_PCs)
    taxa_lines_lengths.append(taxon_line.length)

taxa_lines_lengths_max = max(taxa_lines_lengths)

# Draw biplot circle
####################################
from matplotlib.patches import Circle
from matplotlib.patheffects import withStroke

mpl_circle = Circle(pcoa_plot_centre, max(pcoa_plot_range) / 2, clip_on=False, zorder=10, linewidth=1,
                alpha=0.1, edgecolor='black', facecolor='white',
                path_effects=[withStroke(linewidth=5, foreground='w')])

ax12.add_artist(mpl_circle)


# Draw biplot arrows for top 5 taxa
####################################
arrows = []

for taxon in top_taxa_for_biplot[::-1]:
    taxon_biplot_PCs = biplot_jsd_genus_feature_PCs_df['PC1'][taxon], biplot_jsd_genus_feature_PCs_df['PC2'][taxon]
    
    taxon_line = sympy.Segment2D(pcoa_plot_centre, taxon_biplot_PCs)
    
    #intersection of the taxon line (or Segment) with the circle surrounding the sample scatters
    intersec = sympy.intersection(sympy_circle, taxon_line)
    
    #the line up until the intersection with the circle
    taxon_line_to_intersec = sympy.Segment2D(pcoa_plot_centre, intersec[0])
      
    dx = float(intersec[0][0]) - float(pcoa_plot_centre[0])
    dy = float(intersec[0][1]) - float(pcoa_plot_centre[1])
    
    #calculate scaled values
    line_scale = (taxon_line.length / taxa_lines_lengths_max)
    intersec_scaled = intersec[0].scale(float(line_scale), float(line_scale), pt=pcoa_plot_centre)
    
    dx_scaled = float(intersec_scaled[0]) - float(pcoa_plot_centre[0])
    dy_scaled = float(intersec_scaled[1]) - float(pcoa_plot_centre[1])
    
    #finally draw the arrow
    arrow = ax12.arrow(x=float(pcoa_plot_centre[0]), y=float(pcoa_plot_centre[1]), dx=dx_scaled, dy=dy_scaled,
              color=taxa_colours[taxon], linewidth=2.5, head_width=0.01)
    arrows.append(arrow)

ax12.set_xlabel("PC1 ({}%)".format(str(round(results['PC1_proportion_explained'], 2))))
ax12.set_ylabel("PC2 ({}%)".format(str(round(results['PC2_proportion_explained'], 2))))
ax12.legend(arrows[::-1], top_taxa_for_biplot)
ax12.set_title('Microbiotyping (PCoA on Jensen-Shannon distance matrix)')
```

Out[19]:

```
Text(0.5,1,'Microbiotyping (PCoA on Jensen-Shannon distance matrix)')
```

### Species-level assignment¶

In [20]:

```
exp_species = exp.Taxonomy.groupby("species")
exp_species.mapping_df['microbiotype'] = microbiotypes

exp_species_microbiotypes = exp_species.to_relative_abundance().groupby('microbiotype')

results['mra_staph_aureus_microbiotype_2'] = round(exp_species_microbiotypes.data_df.T['s__aureus']['microbiotype_2'], 2)
results['mra_staph_aureus_microbiotype_1'] = round(exp_species_microbiotypes.data_df.T['s__aureus']['microbiotype_1'], 2)
results['mra_staph_aureus_microbiotype_3'] = round(exp_species_microbiotypes.data_df.T['s__aureus']['microbiotype_3'], 2)

fig = exp_species.apply(TopAbundantObservations(10)).to_relative_abundance().groupby('microbiotype').plot(backend='matplotlib')
```

Although species-level assignment should always be **taken cautiously**, the result above showed an interesting clustering of Staphylococcus aureus in microbiotype 2.

In [21]:

```
exp_rel_abundance = exp.to_relative_abundance()
exp_staph = exp_rel_abundance[exp_rel_abundance.Taxonomy.genus == 'g__Staphylococcus'].Taxonomy.groupby("species")

staph_df = exp_staph.groupby("microbiotype").data_df

display(staph_df.apply(round, args=(2,)).astype("str") + "%")

fig = pyplot.figure(figsize=(14, 8))
grid = pyplot.GridSpec(1, 1)
ax_staph = fig.add_subplot(grid[0,0])
ax_staph.set_ylim(0,100)
ax_staph.set_ylabel("Relative Abundance (%)")
bars_staph = []
colours_staph = ['goldenrod', 'palegoldenrod', 'indianred']

for i, arr in enumerate(staph_df.as_matrix()):
    bar_ = ax_staph.bar([i+1,i+5,i+9], arr, width=1, color=colours_staph[i])
    rounded = [str(round(a, 2)) + "%" for a in arr]
    bars_staph.append(bar_[0])
    ax_staph.text(i+1, arr[0]+5, rounded[0], horizontalalignment='center')
    ax_staph.text(i+5, arr[1]+5, rounded[1], horizontalalignment='center')
    ax_staph.text(i+9, arr[2]+5, rounded[2], horizontalalignment='center')
    
ax_staph.legend(bars_staph, ['Staph aureus', 'Staph epidermidis', 'Staph sp. unidentified/other'])
ax_staph.set_xticks([2,6,10])
ax_staph.set_xticklabels(["microbiotype_1", "microbiotype_2", "microbiotype_3"])
ax_staph.set_title("Species-level assignment of Staphylococci in the three microbiotypes.")
```

| microbiotype | microbiotype\_1 | microbiotype\_2 | microbiotype\_3 |
| --- | --- | --- | --- |
| s\_\_aureus | 1.36% | 47.81% | 0.3% |
| s\_\_epidermidis | 8.43% | 22.72% | 0.53% |
| s\_\_unidentified (g\_\_Staphylococcus) | 0.9% | 4.43% | 0.09% |

Out[21]:

```
Text(0.5,1,'Species-level assignment of Staphylococci in the three microbiotypes.')
```

##### Differential abundance using ANCOM confirms the differential abundance of Staph aureus and epidermidis between the three microbiotypes.¶

In [22]:

```
from skbio.stats.composition import ancom, multiplicative_replacement
mult_rep_species_df = pd.DataFrame(multiplicative_replacement(exp_species.data_df), index=exp_species.data_df.index, columns=exp_species.data_df.columns)

ancom_results_species = ancom(mult_rep_species_df.T, exp_species.mapping_df.reindex(exp_species.data_df.T.index)['microbiotype'])
ancom_results_species[0][ancom_results_species[0]['Reject null hypothesis'] == True]
```

```
/home/ahmed/dev/biomenv3/lib/python3.6/site-packages/scipy/stats/stats.py:2926: RuntimeWarning: invalid value encountered in double_scalars
  f = msb / msw
```

Out[22]:

|  | Reject null hypothesis | W |
| --- | --- | --- |
| species |  |  |
| s\_\_aureus | True | 233 |
| s\_\_epidermidis | True | 233 |
| s\_\_unidentified (g\_\_Alloiococcus) | True | 232 |
| s\_\_unidentified (g\_\_Corynebacterium) | True | 233 |
| s\_\_unidentified (g\_\_Haemophilus) | True | 232 |
| s\_\_unidentified (g\_\_Streptococcus) | True | 232 |

# SparCC correlation coefficients and p values¶

Based on the species-level assignment results above, we are now interested in the relationship between Corynebacteria and Staph aureus and epidermidis.

In [23]:

```
corr_pval_df = fastspar_corr_df.astype(str) + " (p=" + fastspar_pvalues_df.astype(str) + ")"
corr_pval_df_important = corr_pval_df.drop(other_taxa, axis=0, errors='ignore').drop(other_taxa, axis=1, errors='ignore')

results['sparcc_corr_staph_aureus_coryne'] = round(fastspar_corr_df['g__Corynebacterium']['s__aureus'], 3)
results['sparcc_pval_staph_aureus_coryne'] = round(fastspar_pvalues_df['g__Corynebacterium']['s__aureus'], 3)
results['sparcc_corr_staph_epi_coryne'] = round(fastspar_corr_df['g__Corynebacterium']['s__epidermidis'], 3)
results['sparcc_pval_staph_epi_coryne'] = round(fastspar_pvalues_df['g__Corynebacterium']['s__epidermidis'], 3)
results['sparcc_corr_staph_epi_staph_aureus'] = round(fastspar_corr_df['s__epidermidis']['s__aureus'], 3)
results['sparcc_pval_staph_epi_staph_aureus'] = round(fastspar_pvalues_df['s__epidermidis']['s__aureus'], 3)

corr_pval_df_coryne_staph = corr_pval_df_important[['g__Corynebacterium', 's__aureus', 's__epidermidis']]\
                                .T[['g__Corynebacterium', 's__aureus', 's__epidermidis']]

display(corr_pval_df_coryne_staph)
```

| #OTU ID | g\_\_Corynebacterium | s\_\_aureus | s\_\_epidermidis |
| --- | --- | --- | --- |
| g\_\_Corynebacterium | 1.0 (p=1.0) | -0.3392 (p=0.001) | 0.2708 (p=0.001) |
| s\_\_aureus | -0.3392 (p=0.001) | 1.0 (p=1.0) | -0.2693 (p=0.001) |
| s\_\_epidermidis | 0.2708 (p=0.001) | -0.2693 (p=0.001) | 1.0 (p=1.0) |

## Exploring the constitution of "microbiotype 3"¶

We then sought to explore the "more varied" microbiotype 3, and we initially applied the same K-Means algorithm (run on the first two Principal Components) that was used to define the three microbiotypes.
But since one has to provide the algorithm with the \_n*clusters* and we did not know how many natural clusters are in the data, we tried several; but demonstrated here below is n\_clusters=5 (based on the "core organisms" reported in our other manuscript Paramasivan et al).

In [24]:

```
three_PCs_thirdcluster_df = joined_df[joined_df['microbiotype']=='microbiotype_3'][['PC1', 'PC2', 'PC3']]

from sklearn.cluster import KMeans
kmeans_third_cluster = KMeans(n_clusters=5, random_state=0).fit(three_PCs_thirdcluster_df[['PC1', 'PC2']])

#add the cluster labels back to our Experiment object
clusters_third_cluster = pd.Series(["thirdcluster_" + str(x + 1) for x in kmeans_third_cluster.labels_],
                                index=three_PCs_thirdcluster_df.index, name='thirdcluster')
exp_genus.mapping_df['thirdcluster'] = clusters_third_cluster

display(exp_genus.mapping_df['thirdcluster'].value_counts())

exp_genus_other.groupby('thirdcluster').plot(backend="matplotlib")

import seaborn
seaborn.lmplot('PC1', 'PC2', hue='thirdcluster', fit_reg=False, data=three_PCs_thirdcluster_df.join(clusters_third_cluster))
```

```
thirdcluster_2    34
thirdcluster_3    18
thirdcluster_4     8
thirdcluster_1     6
thirdcluster_5     5
Name: thirdcluster, dtype: int64
```

Out[24]:

```
<seaborn.axisgrid.FacetGrid at 0x7fa43f0b9b38>
```

We notice that there is poor definition of the clusters: Since the samples in this microbiotype are more varied,
a K-Means algorithm run on the first two Principal Components would not be enough to obtain adequate clustering, since a lot more information has already been lost to the dimensionality reduction. We then sought a different algorithm.

We thus employed the hieararchical density-based clustering algorithm **"hdbscan" (McInnes, 2017)** on the full-dimensional OTU table.  
One advantage of this algorithm is that it can estimate the number of clusters, without a priori specification by the user.  
This algorithm also has the ability to detect "outliers" that fail to cluster with the rest of the groups and detaches them into a separate "Miscellaneous/Other" group. We ran this algorithm on samples in Microbiotype 3

In [25]:

```
import hdbscan
clusterer = hdbscan.HDBSCAN()

full_dimensional_for_hdbscan = three_PCs_thirdcluster_df.join(exp_genus.data_df.T).drop(['PC1', 'PC2', 'PC3'], axis=1)
clusterer.fit(full_dimensional_for_hdbscan)

#add the cluster labels back to our Experiment object
from pandas import Series
hdb_clusters_third_cluster = Series(["hdb_thirdcluster_" + str(x) for x in clusterer.labels_],
                                index=three_PCs_thirdcluster_df.index, name='hdb_thirdcluster')
exp_genus.mapping_df['hdb_thirdcluster'] = hdb_clusters_third_cluster

#plot the 2 PCS with labeled samples
import seaborn
seaborn.lmplot('PC1', 'PC2', hue='hdb_thirdcluster', fit_reg=False, data=three_PCs_thirdcluster_df.join(hdb_clusters_third_cluster))

#plot the hdbscan assigned clusters for microbiotype 3
from omicexperiment.transforms.observation import TopAbundantObservations
exp_genus_thirdcluster = exp_genus.to_relative_abundance().apply(TopAbundantObservations(10)).groupby('hdb_thirdcluster')

microbiotype_3_replace_dict = {}
microbiotype_3_replace_dict[exp_genus_thirdcluster.data_df.T['g__Streptococcus'].sort_values(ascending=False).index[0]] = 'microbiotype_3S'
microbiotype_3_replace_dict[exp_genus_thirdcluster.data_df.T['g__Haemophilus'].sort_values(ascending=False).index[0]] = 'microbiotype_3H'
microbiotype_3_replace_dict[exp_genus_thirdcluster.data_df.T['g__Moraxella'].sort_values(ascending=False).index[0]] = 'microbiotype_3M'
microbiotype_3_replace_dict[exp_genus_thirdcluster.data_df.T['g__Pseudomonas'].sort_values(ascending=False).index[0]] = 'microbiotype_3P'
microbiotype_3_replace_dict[exp_genus_thirdcluster.data_df.T['Other'].sort_values(ascending=False).index[0]] = 'microbiotype_3O'

print(microbiotype_3_replace_dict)

microbiotype_3_df = exp_genus_thirdcluster.data_df.rename(microbiotype_3_replace_dict, axis=1)

submicrobiotypes_3 = ['microbiotype_3S', 'microbiotype_3H', 'microbiotype_3M', 'microbiotype_3P', 'microbiotype_3O']
exp_genus_thirdcluster.data_df = microbiotype_3_df[submicrobiotypes_3]
fig = exp_genus_thirdcluster.plot(backend='matplotlib')
```

```
{'hdb_thirdcluster_2': 'microbiotype_3S', 'hdb_thirdcluster_3': 'microbiotype_3H', 'hdb_thirdcluster_1': 'microbiotype_3M', 'hdb_thirdcluster_0': 'microbiotype_3P', 'hdb_thirdcluster_-1': 'microbiotype_3O'}
```

In [26]:

```
microbiotype_3_counts_series = exp_genus.mapping_df['hdb_thirdcluster'].replace(microbiotype_3_replace_dict).value_counts()

display(microbiotype_3_counts_series)

results['n_microbiotype_3S'] = microbiotype_3_counts_series['microbiotype_3S']
results['n_microbiotype_3H'] = microbiotype_3_counts_series['microbiotype_3H']
results['n_microbiotype_3M'] = microbiotype_3_counts_series['microbiotype_3M']
results['n_microbiotype_3P'] = microbiotype_3_counts_series['microbiotype_3P']
results['n_microbiotype_3O'] = microbiotype_3_counts_series['microbiotype_3O']
```

```
microbiotype_3S    21
microbiotype_3O    18
microbiotype_3H    16
microbiotype_3M     9
microbiotype_3P     7
Name: hdb_thirdcluster, dtype: int64
```

In [27]:

```
microbiotype_3_genera_ranges_df = exp_genus_thirdcluster.data_df.T[['g__Streptococcus', 'g__Haemophilus', 'g__Moraxella', 'g__Pseudomonas']].max()

display(microbiotype_3_genera_ranges_df)

results['microbiotype_3_genera_mra_range_min'] = round(microbiotype_3_genera_ranges_df.min(), 2)
results['microbiotype_3_genera_mra_range_max'] = round(microbiotype_3_genera_ranges_df.max(), 2)
```

```
g__Streptococcus    73.488095
g__Haemophilus      95.500000
g__Moraxella        82.638889
g__Pseudomonas      88.857143
dtype: float64
```

In [28]:

```
exp_genus_thirdcluster.data_df
```

Out[28]:

| hdb\_thirdcluster | microbiotype\_3S | microbiotype\_3H | microbiotype\_3M | microbiotype\_3P | microbiotype\_3O |
| --- | --- | --- | --- | --- | --- |
| Other | 15.202381 | 0.640625 | 1.527778 | 4.250000 | 42.625000 |
| g\_\_Alloiococcus | 0.023810 | 0.000000 | 4.388889 | 0.642857 | 0.152778 |
| g\_\_Corynebacterium | 4.428571 | 1.328125 | 2.805556 | 2.107143 | 1.513889 |
| g\_\_Haemophilus | 2.333333 | 95.500000 | 1.055556 | 0.000000 | 5.666667 |
| g\_\_Moraxella | 2.904762 | 0.000000 | 82.638889 | 0.000000 | 3.055556 |
| g\_\_Pseudomonas | 0.345238 | 0.000000 | 1.472222 | 88.857143 | 0.472222 |
| g\_\_Serratia | 0.690476 | 0.000000 | 0.000000 | 0.071429 | 21.638889 |
| g\_\_Staphylococcus | 0.404762 | 0.125000 | 2.638889 | 1.785714 | 1.027778 |
| g\_\_Streptococcus | 73.488095 | 2.406250 | 3.138889 | 1.357143 | 1.625000 |
| g\_\_unidentified (f\_\_Enterobacteriaceae) | 0.178571 | 0.000000 | 0.000000 | 0.928571 | 22.069444 |
| g\_\_unidentified (f\_\_Neisseriaceae) | 0.000000 | 0.000000 | 0.333333 | 0.000000 | 0.152778 |

As seen above, this hdbscan clustering approach on this data appears more reasonable, with each group/cluster dominated by one of the abundant organisms in the dataset, as well as a final "other" group.

In [29]:

```
hdbclusterer = hdbscan.HDBSCAN()
hdbclusterer.fit(exp_genus.data_df.T)
len(hdbclusterer.labels_)
hdbcluster_df = \
exp_genus.data_df.T.join(Series(["hdb_cluster_" + str(x) for x in hdbclusterer.labels_],
                                name='hdbcluster',
                                index=exp_genus.data_df.T.index)).groupby('hdbcluster').mean().T

display(exp_genus.with_data_df(hdbcluster_df).to_relative_abundance().apply(TopAbundantObservations(10)).data_df)
exp_genus.with_data_df(hdbcluster_df).to_relative_abundance().apply(TopAbundantObservations(10))\
.plot(backend='matplotlib')

pyplot.show()
```

| hdbcluster | hdb\_cluster\_-1 | hdb\_cluster\_0 | hdb\_cluster\_1 | hdb\_cluster\_10 | hdb\_cluster\_11 | hdb\_cluster\_12 | hdb\_cluster\_13 | hdb\_cluster\_14 | hdb\_cluster\_15 | hdb\_cluster\_2 | hdb\_cluster\_3 | hdb\_cluster\_4 | hdb\_cluster\_5 | hdb\_cluster\_6 | hdb\_cluster\_7 | hdb\_cluster\_8 | hdb\_cluster\_9 |
| --- | --- | --- | --- | --- | --- | --- | --- | --- | --- | --- | --- | --- | --- | --- | --- | --- | --- |
| cluster |  |  |  |  |  |  |  |  |  |  |  |  |  |  |  |  |  |
| Other | 13.217949 | 1.10 | 0.00 | 1.508333 | 0.333333 | 0.416667 | 0.18750 | 0.041667 | 0.020833 | 0.602941 | 3.71875 | 9.930556 | 1.325 | 2.171875 | 0.559091 | 3.05 | 0.25000 |
| g\_\_Alloiococcus | 2.612821 | 0.00 | 0.00 | 0.083333 | 0.041667 | 0.000000 | 2.12500 | 0.000000 | 0.000000 | 0.000000 | 0.56250 | 0.083333 | 2.400 | 0.000000 | 0.000000 | 0.00 | 15.18750 |
| g\_\_Corynebacterium | 55.574359 | 7.30 | 0.95 | 65.108333 | 84.875000 | 92.000000 | 97.28125 | 99.416667 | 99.791667 | 1.250000 | 1.84375 | 4.138889 | 7.025 | 26.656250 | 1.722727 | 41.95 | 84.15625 |
| g\_\_Haemophilus | 1.707692 | 0.00 | 0.00 | 0.116667 | 0.000000 | 0.000000 | 0.00000 | 0.000000 | 0.000000 | 94.264706 | 0.00000 | 1.361111 | 0.950 | 0.328125 | 0.000000 | 0.00 | 0.00000 |
| g\_\_Moraxella | 3.501282 | 0.00 | 0.00 | 0.000000 | 0.000000 | 0.000000 | 0.00000 | 0.000000 | 0.000000 | 0.000000 | 0.00000 | 0.125000 | 83.525 | 0.000000 | 0.036364 | 0.00 | 0.00000 |
| g\_\_Pseudomonas | 0.607692 | 0.00 | 0.00 | 0.066667 | 0.000000 | 0.000000 | 0.00000 | 0.000000 | 0.000000 | 0.000000 | 88.12500 | 0.402778 | 0.025 | 0.000000 | 0.036364 | 0.00 | 0.00000 |
| g\_\_Serratia | 0.096154 | 89.75 | 0.00 | 0.000000 | 0.083333 | 0.000000 | 0.09375 | 0.000000 | 0.000000 | 0.000000 | 0.06250 | 0.805556 | 0.000 | 0.000000 | 0.100000 | 0.00 | 0.00000 |
| g\_\_Staphylococcus | 16.580769 | 1.85 | 3.30 | 32.733333 | 14.541667 | 7.416667 | 0.21875 | 0.541667 | 0.187500 | 1.617647 | 3.68750 | 0.472222 | 2.275 | 70.328125 | 97.113636 | 54.40 | 0.15625 |
| g\_\_Streptococcus | 3.652564 | 0.00 | 0.00 | 0.158333 | 0.125000 | 0.166667 | 0.00000 | 0.000000 | 0.000000 | 2.264706 | 1.18750 | 79.486111 | 2.475 | 0.375000 | 0.313636 | 0.00 | 0.25000 |
| g\_\_Veillonella | 0.243590 | 0.00 | 0.00 | 0.033333 | 0.000000 | 0.000000 | 0.00000 | 0.000000 | 0.000000 | 0.000000 | 0.00000 | 2.986111 | 0.000 | 0.140625 | 0.000000 | 0.00 | 0.00000 |
| g\_\_unidentified (f\_\_Enterobacteriaceae) | 2.205128 | 0.00 | 95.75 | 0.191667 | 0.000000 | 0.000000 | 0.09375 | 0.000000 | 0.000000 | 0.000000 | 0.81250 | 0.208333 | 0.000 | 0.000000 | 0.118182 | 0.60 | 0.00000 |

# Plot: Master Figure of the microbiotyping manuscript¶

In [30]:

```
import matplotlib.pyplot as plt
import matplotlib.gridspec as gridspec

#set figure and grid
###
fig = pyplot.figure(figsize=(32, 20))
grid = pyplot.GridSpec(3, 10, wspace=0.6, hspace=0.3)
lower_row_of_grid = grid[2,:]
inner_grid = gridspec.GridSpecFromSubplotSpec(1, 3, lower_row_of_grid)

#set colours
###
from matplotlib.colors import to_hex

cmap_tab10 = pyplot.get_cmap('tab10')
colours_tab10_11 = list(cmap_tab10.colors) + [cmap_tab20.colors[0]]
colours_tab10_11 = [to_hex(c) for c in colours_tab10_11]

taxa_colours = {tax: col for tax, col in zip(taxa_plus_other, colours_tab10_11)}

cmap_tab20 = pyplot.get_cmap('tab20')
colours = list(cmap_tab20.colors[::2])
colours = [to_hex(c) for c in colours]
colours_light = list(cmap_tab20.colors[1::2])
colours_light = [to_hex(c) for c in colours_light]
colours_11 = colours + [to_hex(cmap_tab20.colors[::2][0])]
colours_shift1 = colours[1:] + [colours[0]]
colours_light_shift1 = colours_light[1:] + [colours_light[0]]

colours_microbiotypes = ['royalblue', 'orange', 'indianred']


#microbiotypes
#####################
fig_cumsum_df = return_cumsum_df(exp_genus_other.groupby("microbiotype"))
fig_cumsum_df = clean_taxa_in_index(fig_cumsum_df)
ax1 = fig.add_subplot(grid[0,4:9])
ax1.set_ylim(0,100)
ax1.set_ylabel("Relative Abundance (%)")
ax1.set_title("Microbiotypes of the sinonasal cavity")
bars1 = []

for i, arr in enumerate(fig_cumsum_df.as_matrix()):
    bar_ = ax1.bar(fig_cumsum_df.columns, arr, width=0.6, color=colours_11[i])
    bars1.append(bar_[0])

    
#microbiotyping scatter
####################################
ax12 = fig.add_subplot(grid[0:2,0:4])

scatters = []
for i, cluster in enumerate(['microbiotype_1', 'microbiotype_2', 'microbiotype_3']):
    scatter = ax12.scatter(joined_df[joined_df['microbiotype']==cluster]['PC1'],
                   joined_df[joined_df['microbiotype']==cluster]['PC2'],
                   c=colours_microbiotypes[i], alpha=0.7)
    scatters.append(scatter)

ax12.set_xlabel("PC1 ({}%)".format(str(round(results['PC1_proportion_explained'], 2))))
ax12.set_ylabel("PC2 ({}%)".format(str(round(results['PC2_proportion_explained'], 2))))
ax12.legend(scatters, ['microbiotype_1', 'microbiotype_2', 'microbiotype_3'])
ax12.set_title('The (Jensen-Shannon) PCoA Biplot.\nFeatures are projected into the principal coordinates matrix and represented as scaled arrows.')

#calculations required for biplot
####################################
pcoa_plot_centre = joined_df[['PC1', 'PC2']].apply(lambda x: x.min() + ((np.sqrt((x.max() - x.min())**2) / 2)))
pcoa_plot_centre = pcoa_plot_centre['PC1'], pcoa_plot_centre['PC2']
pcoa_plot_centre = sympy.Rational(str(pcoa_plot_centre[0])), sympy.Rational(str(pcoa_plot_centre[1]))

pcoa_plot_range = joined_df[['PC1', 'PC2']].apply(lambda x: np.sqrt((x.max() - x.min())**2))
pcoa_plot_range = pcoa_plot_range['PC1'], pcoa_plot_range['PC2']
sympy_circle_radius = sympy.Rational(str(max(pcoa_plot_range) / 2))
sympy_circle = sympy.Circle(pcoa_plot_centre, sympy_circle_radius)

#here we choose top five taxa
biplot_n_taxa_arrows = 5
results['biplot_n_taxa_arrows'] = biplot_n_taxa_arrows
top_taxa_for_biplot = taxa_not_other[:biplot_n_taxa_arrows]

#calculate longest of the taxa lines - required for scaling the biplot feature arrows
taxa_lines_lengths = []

for taxon in top_taxa_for_biplot:
    taxon_biplot_PCs = biplot_jsd_genus_feature_PCs_df['PC1'][taxon], biplot_jsd_genus_feature_PCs_df['PC2'][taxon]
    taxon_line = sympy.Segment2D(pcoa_plot_centre, taxon_biplot_PCs)
    taxa_lines_lengths.append(taxon_line.length)

taxa_lines_lengths_max = max(taxa_lines_lengths)

# Draw biplot circle
####################################
from matplotlib.patches import Circle
from matplotlib.patheffects import withStroke

mpl_circle = Circle(pcoa_plot_centre, max(pcoa_plot_range) / 2, clip_on=False, zorder=10, linewidth=1,
                alpha=0.1, edgecolor='black', facecolor='white',
                path_effects=[withStroke(linewidth=5, foreground='w')])

ax12.add_artist(mpl_circle)

# Draw biplot arrows for top 5 taxa
####################################
arrows = []

for taxon in top_taxa_for_biplot[::-1]:
    taxon_biplot_PCs = biplot_jsd_genus_feature_PCs_df['PC1'][taxon], biplot_jsd_genus_feature_PCs_df['PC2'][taxon]
    
    taxon_line = sympy.Segment2D(pcoa_plot_centre, taxon_biplot_PCs)
    
    #intersection of the taxon line (or Segment) with the circle surrounding the sample scatters
    intersec = sympy.intersection(sympy_circle, taxon_line)
    
    #the line up until the intersection with the circle
    taxon_line_to_intersec = sympy.Segment2D(pcoa_plot_centre, intersec[0])
      
    dx = float(intersec[0][0]) - float(pcoa_plot_centre[0])
    dy = float(intersec[0][1]) - float(pcoa_plot_centre[1])
    
    #calculate scaled values
    line_scale = (taxon_line.length / taxa_lines_lengths_max)
    intersec_scaled = intersec[0].scale(float(line_scale), float(line_scale), pt=pcoa_plot_centre)
    
    dx_scaled = float(intersec_scaled[0]) - float(pcoa_plot_centre[0])
    dy_scaled = float(intersec_scaled[1]) - float(pcoa_plot_centre[1])
    
    #finally draw the arrow
    arrow = ax12.arrow(x=float(pcoa_plot_centre[0]), y=float(pcoa_plot_centre[1]), dx=dx_scaled, dy=dy_scaled,
              color=taxa_colours[taxon], linewidth=3, head_width=0.01, length_includes_head=True)
    arrows.append(arrow)

    
#Coryne and Staph histograms
####################################
density=False
ax20 = fig.add_subplot(inner_grid[0,0])
ax20.hist(exp_genus_other_microbiotype1.data_df.T['g__Corynebacterium'], color='tab:green', histtype='step', density=density, range=(0,100))
ax20.hist(exp_genus_other_microbiotype1.data_df.T['g__Staphylococcus'], color= 'tab:grey', histtype='step', density=density, range=(0,100))
ax20.legend()
ax20.set_xlabel('Relative abundance (%)')
ax20.set_ylabel('Number of samples')
ax20.set_title('Distribution of Corynebacteria and Staphylococci\nin Microbiotype 1')

ax21 = fig.add_subplot(inner_grid[0,1], sharey=ax20)
ax21.hist(exp_genus_other_microbiotype2.data_df.T['g__Corynebacterium'], color='tab:green', histtype='step', density=density, range=(0,100))
ax21.hist(exp_genus_other_microbiotype2.data_df.T['g__Staphylococcus'], color= 'tab:grey', histtype='step', density=density, range=(0,100))
ax21.legend()
ax21.set_xlabel('Relative abundance (%)')
ax21.set_ylabel('Number of samples')
ax21.set_title('Distribution of Corynebacteria and Staphylococci\nin Microbiotype 2')

ax22 = fig.add_subplot(inner_grid[0,2], sharey=ax20)
hist22_coryne = ax22.hist(exp_genus_other_microbiotype3.data_df.T['g__Corynebacterium'], color='tab:green', histtype='step', density=density, range=(0,100))
hist22_staph = ax22.hist(exp_genus_other_microbiotype3.data_df.T['g__Staphylococcus'], color= 'tab:grey', histtype='step', density=density, range=(0,100))
ax22.legend()
ax22.set_xlabel('Relative abundance (%)')
ax22.set_ylabel('Number of samples')
ax22.set_title('Distribution of Corynebacteria and Staphylococci\nin Microbiotype 3')


#Staph species
##############
exp_rel_abundance = exp.to_relative_abundance()
exp_staph = exp_rel_abundance[exp_rel_abundance.Taxonomy.genus == 'g__Staphylococcus'].Taxonomy.groupby("species")

staph_df = exp_staph.groupby("microbiotype").data_df

ax_staph = fig.add_subplot(grid[1,7:])
ax_staph.set_ylim(0,100)
ax_staph.set_ylabel("Relative Abundance (%)")
ax_staph.set_title("Microbiotypes of the sinonasal cavity")
bars_staph = []
colours_staph = ['goldenrod', 'palegoldenrod', 'indianred']

for i, arr in enumerate(staph_df.as_matrix()):
    bar_ = ax_staph.bar([i+1,i+5,i+9], arr, width=1, color=colours_staph[i])
    rounded = [str(round(a, 2)) + "%" for a in arr]
    bars_staph.append(bar_[0])
    ax_staph.text(i+1, arr[0]+5, rounded[0], horizontalalignment='center')
    ax_staph.text(i+5, arr[1]+5, rounded[1], horizontalalignment='center')
    ax_staph.text(i+9, arr[2]+5, rounded[2], horizontalalignment='center')
    
ax_staph.legend(bars_staph, ['Staph aureus', 'Staph epidermidis', 'Staph sp. unidentified/other'])
ax_staph.set_xticks([2,6,10])
ax_staph.set_xticklabels(["microbiotype_1", "microbiotype_2", "microbiotype_3"])
ax_staph.set_title("Species-level assignment of Staphylococci in the three microbiotypes.")

#third microbiotype
#######################
fig_thirdcluster_cumsum_df = return_cumsum_df(exp_genus_thirdcluster)

ax_third = fig.add_subplot(grid[1,4:7])
ax_third.set_ylim(0,100)
ax_third.set_ylabel("Relative Abundance (%)")
ax_third.set_title("Subgroups of Microbiotype 3")

bars_third = []

for i, arr in enumerate(fig_thirdcluster_cumsum_df.as_matrix()):
    bar_ = ax_third.bar(fig_thirdcluster_cumsum_df.columns, arr, width=0.6, color=colours_11[i])
    bars_third.append(bar_[0])

#legend
##############
ax_leg = fig.add_subplot(grid[0,9])
ax_leg.axis('off')
ax_leg.legend(bars1,list(fig_cumsum_df.index), loc='lower center', title="Taxonomy composition - genus level\n")

#figtexts
##############
pyplot.figtext(0.11,0.90, "A.", fontsize=16, axes=ax12)
pyplot.figtext(0.43,0.90, "B.", fontsize=16, axes=ax1)
pyplot.figtext(0.11,0.35, "C.", fontsize=16, axes=ax20)
pyplot.figtext(0.43,0.63, "D.", fontsize=16, axes=ax_staph)
pyplot.figtext(0.67,0.63, "E.", fontsize=16, axes=ax_third)

fig.savefig("./results/microbiotyping/figures/microbiotypes_taxa_staph_coryne.jpg", bbox_inches="tight", dpi=300)
```

# Prevalence and distribution of the microbiotypes¶

In [31]:

```
def crosstab_percentages(dataframe, column_axis0, column_axis1, axis=0, percent=100):
    
    if axis==0:
        tbl_crosstab = dataframe.groupby(column_axis0)[column_axis1].value_counts().to_frame().unstack()
        tbl_crosstab = tbl_crosstab.T
    elif axis==1:
        tbl_crosstab = dataframe.groupby(column_axis1)[column_axis0].value_counts().to_frame().unstack()
        tbl_crosstab = tbl_crosstab.T
        
    tbl_crosstab_percent = tbl_crosstab.apply(lambda x: x / x.sum()) * percent
    
    if axis==0:
        tbl_crosstab_percent = tbl_crosstab_percent.reset_index(level=1).set_index(column_axis1)
        tbl_crosstab_percent = tbl_crosstab_percent.T
    elif axis==1:
        tbl_crosstab_percent = tbl_crosstab_percent.reset_index(level=1).set_index(column_axis0)
    
    return tbl_crosstab_percent

def crosstab_with_percentages(dataframe, column_axis0, column_axis1, axis=0, percent=100, decimal_places=2):
    
    if axis==0:
        tbl_crosstab = dataframe.groupby(column_axis0)[column_axis1].value_counts().to_frame().unstack()
        tbl_crosstab = tbl_crosstab.T
    elif axis==1:
        tbl_crosstab = dataframe.groupby(column_axis1)[column_axis0].value_counts().to_frame().unstack()
        tbl_crosstab = tbl_crosstab.T
        
    tbl_crosstab_percent = tbl_crosstab.apply(lambda x: x / x.sum()) * percent
    
    #round to decimal places as specified
    tbl_crosstab_percent = tbl_crosstab_percent.apply(round, args=(decimal_places,))

    tbl_crosstab_str = tbl_crosstab.astype('str').stack() \
                                        + " (" \
                                        + tbl_crosstab_percent.astype('str').stack() \
                                        + "%)"

    tbl_crosstab_str = tbl_crosstab_str.unstack()
    if axis==0:
        tbl_crosstab_str = tbl_crosstab_str.reset_index(level=1).set_index(column_axis1)
        tbl_crosstab_str = tbl_crosstab_str.T
    elif axis==1:
        tbl_crosstab_str = tbl_crosstab_str.reset_index(level=1).set_index(column_axis0)
    
    return tbl_crosstab_str
```

#### Prevalence¶

In [32]:

```
tbl_dist_microbiotypes = exp_genus.mapping_df['microbiotype'].value_counts()
#tbl_dist_microbiotypes
tbl_dist_microbiotypes_percent = exp_genus.mapping_df['microbiotype'].value_counts() / exp_genus.mapping_df['microbiotype'].value_counts().sum() * 100

print("N for each microbiotype:")
display(tbl_dist_microbiotypes.to_frame())

print("Prevalence (%) of each microbiotype:")
display(tbl_dist_microbiotypes_percent.to_frame())

results['n_microbiotype1'] = tbl_dist_microbiotypes['microbiotype_1']
results['n_microbiotype2'] = tbl_dist_microbiotypes['microbiotype_2']
results['n_microbiotype3'] = tbl_dist_microbiotypes['microbiotype_3']
results['n_microbiotype1_percent'] = round(tbl_dist_microbiotypes_percent['microbiotype_1'], 1) #round to 2?
results['n_microbiotype2_percent'] = round(tbl_dist_microbiotypes_percent['microbiotype_2'], 1) #round to 2?
results['n_microbiotype3_percent'] = round(tbl_dist_microbiotypes_percent['microbiotype_3'], 1) #round to 2?
```

```
N for each microbiotype:
```

|  | microbiotype |
| --- | --- |
| microbiotype\_1 | 222 |
| microbiotype\_2 | 117 |
| microbiotype\_3 | 71 |

```
Prevalence (%) of each microbiotype:
```

|  | microbiotype |
| --- | --- |
| microbiotype\_1 | 54.146341 |
| microbiotype\_2 | 28.536585 |
| microbiotype\_3 | 17.317073 |

#### Distribution by diagnosis¶

In [33]:

```
#Distribution by diagnosis
tbl_dist_microbiotypes_diagnosis = pd.crosstab(exp_genus.mapping_df['diagnosis'], exp_genus.mapping_df['microbiotype'])
#display(tbl_dist_microbiotypes_diagnosis)

display(crosstab_with_percentages(exp_genus.mapping_df, "diagnosis", "microbiotype", axis=0, percent=100))
from scipy.stats import chi2_contingency

print("Chi squared test:")
print(chi2_contingency(tbl_dist_microbiotypes_diagnosis.as_matrix())[0:2])

tbl_dist_microbiotypes_diagnosis_percent = crosstab_percentages(exp_genus.mapping_df, "diagnosis", "microbiotype", axis=0, percent=100)
```

| microbiotype | microbiotype\_1 | microbiotype\_2 | microbiotype\_3 |
| --- | --- | --- | --- |
| diagnosis |  |  |  |
| CRSsNP | 56 (56.57%) | 27 (27.27%) | 16 (16.16%) |
| CRSwNP | 85 (49.42%) | 48 (27.91%) | 39 (22.67%) |
| Control | 81 (58.27%) | 42 (30.22%) | 16 (11.51%) |

```
Chi squared test:
(7.1040247035876991, 0.13049205419258089)
```

#### Distribution by continent¶

In [34]:

```
#Distribution by continent
tbl_dist_microbiotypes_continent = pd.crosstab(exp_genus.mapping_df['continent'], exp_genus.mapping_df['microbiotype'])

#display(tbl_dist_microbiotypes_continent)

display(crosstab_with_percentages(exp_genus.mapping_df, "continent", "microbiotype", axis=0, percent=100))

from scipy.stats import chi2_contingency

print("Chi squared test:")
print(chi2_contingency(tbl_dist_microbiotypes_continent.as_matrix())[0:2])


tbl_dist_microbiotypes_continent_percent = crosstab_percentages(exp_genus.mapping_df, "continent", "microbiotype", axis=0, percent=100)
microbiotype2_in_europe_percent = round(tbl_dist_microbiotypes_continent_percent['microbiotype_2']['Europe'], 2)
microbiotype3_in_asia_percent = round(tbl_dist_microbiotypes_continent_percent['microbiotype_3']['Asia'], 2)
```

| microbiotype | microbiotype\_1 | microbiotype\_2 | microbiotype\_3 |
| --- | --- | --- | --- |
| continent |  |  |  |
| Asia | 27 (69.23%) | 11 (28.21%) | 1 (2.56%) |
| Australasia | 67 (61.47%) | 23 (21.1%) | 19 (17.43%) |
| Europe | 7 (18.42%) | 22 (57.89%) | 9 (23.68%) |
| North\_America | 89 (56.33%) | 43 (27.22%) | 26 (16.46%) |
| South\_America | 32 (48.48%) | 18 (27.27%) | 16 (24.24%) |

```
Chi squared test:
(33.616809948128576, 4.7661482119656179e-05)
```

### Plot: Prevalence and Distribution by Diagnosis and Continent¶

In [35]:

```
tbl_dist_microbiotypes_diagnosis_cumsum_matrix = np.flip(np.cumsum(tbl_dist_microbiotypes_diagnosis_percent.T.as_matrix(), axis=0), axis=0)
tbl_dist_microbiotypes_continent_cumsum_matrix = np.flip(np.cumsum(tbl_dist_microbiotypes_continent_percent.T.as_matrix(), axis=0), axis=0)

axis_group_labels_diagnosis = [i for i in tbl_dist_microbiotypes_diagnosis_percent.T.columns]
axis_group_labels_continent = [i for i in tbl_dist_microbiotypes_continent_percent.T.columns]

colours = ['royalblue', 'orange', 'forestgreen']
#colours = ['royalblue', 'orange', 'crimson']
colours_reversed = [c for c in reversed(colours)]

fig = pyplot.figure(figsize=(12, 12))
grid = pyplot.GridSpec(5, 5, wspace=0.2, hspace=0.4)
main_ax = fig.add_subplot(grid[0:2, 0:2])

tbl_dist_microbiotypes_percent.apply(round, args=(2,))\
.plot(kind='pie',     # with the labels being officer names
    shadow=False,
    colors=colours,
    # with the start angle at 90%
    startangle=0,
    explode=(0.01, 0.01, 0.01),
    # with the percent listed as a fraction
    autopct='%1.2f%%',)

main_ax.set_title("Prevalence of the sinonasal microbiotypes")

ax2 = fig.add_subplot(grid[0:1, 3:])
ax3 = fig.add_subplot(grid[1:2, 3:], sharex=ax2)

for i, arr in enumerate(tbl_dist_microbiotypes_diagnosis_cumsum_matrix):
    ax2.barh(axis_group_labels_diagnosis, arr, color=colours_reversed[i])
    #for ind_arr, val in enumerate(arr):
    #    ax2.annotate(str(round(val, 0)) + "%", (val-2, ind_arr), (val-2, ind_arr), ha="right")
        
ax2.set_title("Distribution of microbiotypes by diagnosis")

for i, arr in enumerate(tbl_dist_microbiotypes_continent_cumsum_matrix):
    ax3.barh(axis_group_labels_continent, arr, color=colours_reversed[i])
ax3.set_title("Distribution of microbiotypes by continent")

pyplot.xlim(0, 100)

fig.savefig("./results/microbiotyping/figures/microbiotypes_distribution.jpg", bbox_inches="tight", dpi=300)
```

## Clinical covariates: Contingency tables and significance testing¶

In [36]:

```
display(crosstab_with_percentages(exp_genus.mapping_df, "CRS", "microbiotype", axis=0, percent=100))
display(chi2_contingency(crosstab_percentages(exp_genus.mapping_df, "CRS", "microbiotype", axis=0, percent=100)))

display(crosstab_with_percentages(exp_genus.mapping_df, "diagnosis", "microbiotype", axis=0, percent=100))
display(chi2_contingency(crosstab_percentages(exp_genus.mapping_df, "diagnosis", "microbiotype", axis=0, percent=100)))

display(crosstab_with_percentages(exp_genus.mapping_df, "continent", "microbiotype", axis=0, percent=100))
display(chi2_contingency(crosstab_percentages(exp_genus.mapping_df, "continent", "microbiotype", axis=0, percent=100)))

display(crosstab_with_percentages(exp_genus.mapping_df, "prev_surgery_group", "microbiotype", axis=0, percent=100))
display(chi2_contingency(crosstab_percentages(exp_genus.mapping_df, "prev_surgery_group", "microbiotype", axis=0, percent=100)))

display(crosstab_with_percentages(exp_genus.mapping_df, "asthma_english", "microbiotype", axis=0, percent=100))
display(chi2_contingency(crosstab_percentages(exp_genus.mapping_df, "asthma_english", "microbiotype", axis=0, percent=100)))

display(crosstab_with_percentages(exp_genus.mapping_df, "aspirin_sensitivity_english", "microbiotype", axis=0, percent=100))
display(chi2_contingency(crosstab_percentages(exp_genus.mapping_df, "aspirin_sensitivity_english", "microbiotype", axis=0, percent=100)))

display(crosstab_with_percentages(exp_genus.mapping_df, "diabetes_english", "microbiotype", axis=0, percent=100))
display(chi2_contingency(crosstab_percentages(exp_genus.mapping_df, "diabetes_english", "microbiotype", axis=0, percent=100)))

display(crosstab_with_percentages(exp_genus.mapping_df, "smoker_current_english", "microbiotype", axis=0, percent=100))
display(chi2_contingency(crosstab_percentages(exp_genus.mapping_df, "smoker_current_english", "microbiotype", axis=0, percent=100)))
```

| microbiotype | microbiotype\_1 | microbiotype\_2 | microbiotype\_3 |
| --- | --- | --- | --- |
| CRS |  |  |  |
| No | 81 (58.27%) | 42 (30.22%) | 16 (11.51%) |
| Yes | 141 (52.03%) | 75 (27.68%) | 55 (20.3%) |

```
(2.8910780336361563,
 0.23561903969638162,
 2,
 array([[ 55.1514508 ,  28.94555205,  15.90299716],
        [ 55.1514508 ,  28.94555205,  15.90299716]]))
```

| microbiotype | microbiotype\_1 | microbiotype\_2 | microbiotype\_3 |
| --- | --- | --- | --- |
| diagnosis |  |  |  |
| CRSsNP | 56 (56.57%) | 27 (27.27%) | 16 (16.16%) |
| CRSwNP | 85 (49.42%) | 48 (27.91%) | 39 (22.67%) |
| Control | 81 (58.27%) | 42 (30.22%) | 16 (11.51%) |

```
(4.7221133287424788,
 0.31701656327472139,
 4,
 array([[ 54.7525475 ,  28.46517712,  16.78227538],
        [ 54.7525475 ,  28.46517712,  16.78227538],
        [ 54.7525475 ,  28.46517712,  16.78227538]]))
```

| microbiotype | microbiotype\_1 | microbiotype\_2 | microbiotype\_3 |
| --- | --- | --- | --- |
| continent |  |  |  |
| Asia | 27 (69.23%) | 11 (28.21%) | 1 (2.56%) |
| Australasia | 67 (61.47%) | 23 (21.1%) | 19 (17.43%) |
| Europe | 7 (18.42%) | 22 (57.89%) | 9 (23.68%) |
| North\_America | 89 (56.33%) | 43 (27.22%) | 26 (16.46%) |
| South\_America | 32 (48.48%) | 18 (27.27%) | 16 (24.24%) |

```
(74.644305542869049,
 5.81159185566095e-13,
 8,
 array([[ 50.78673484,  32.33773992,  16.87552524],
        [ 50.78673484,  32.33773992,  16.87552524],
        [ 50.78673484,  32.33773992,  16.87552524],
        [ 50.78673484,  32.33773992,  16.87552524],
        [ 50.78673484,  32.33773992,  16.87552524]]))
```

| microbiotype | microbiotype\_1 | microbiotype\_2 | microbiotype\_3 |
| --- | --- | --- | --- |
| prev\_surgery\_group |  |  |  |
| No | 92 (47.18%) | 57 (29.23%) | 46 (23.59%) |
| Yes | 130 (60.47%) | 60 (27.91%) | 25 (11.63%) |

```
(5.7332915534719531,
 0.05688942674987045,
 2,
 array([[ 53.82230173,  28.56887299,  17.60882528],
        [ 53.82230173,  28.56887299,  17.60882528]]))
```

| microbiotype | microbiotype\_1 | microbiotype\_2 | microbiotype\_3 |
| --- | --- | --- | --- |
| asthma\_english |  |  |  |
| No | 162 (56.45%) | 81 (28.22%) | 44 (15.33%) |
| Yes | 55 (51.4%) | 31 (28.97%) | 21 (19.63%) |

```
(0.77346732584770084,
 0.67927198615124929,
 2,
 array([[ 53.9239311 ,  28.59747957,  17.47858934],
        [ 53.9239311 ,  28.59747957,  17.47858934]]))
```

| microbiotype | microbiotype\_1 | microbiotype\_2 | microbiotype\_3 |
| --- | --- | --- | --- |
| aspirin\_sensitivity\_english |  |  |  |
| No | 202 (55.34%) | 106 (29.04%) | 57 (15.62%) |
| Yes | 12 (48.0%) | 5 (20.0%) | 8 (32.0%) |

```
(7.8256270581873579,
 0.019984195774319591,
 2,
 array([[ 51.67123288,  24.52054795,  23.80821918],
        [ 51.67123288,  24.52054795,  23.80821918]]))
```

| microbiotype | microbiotype\_1 | microbiotype\_2 | microbiotype\_3 |
| --- | --- | --- | --- |
| diabetes\_english |  |  |  |
| No | 189 (54.94%) | 98 (28.49%) | 57 (16.57%) |
| Yes | 22 (55.0%) | 11 (27.5%) | 7 (17.5%) |

```
(0.042877484369132003,
 0.97878943413522668,
 2,
 array([[ 54.97093023,  27.99418605,  17.03488372],
        [ 54.97093023,  27.99418605,  17.03488372]]))
```

| microbiotype | microbiotype\_1 | microbiotype\_2 | microbiotype\_3 |
| --- | --- | --- | --- |
| smoker\_current\_english |  |  |  |
| No | 204 (54.4%) | 110 (29.33%) | 61 (16.27%) |
| Yes | 15 (57.69%) | 4 (15.38%) | 7 (26.92%) |

```
(7.0769825567208056,
 0.029057133153877392,
 2,
 array([[ 56.04615385,  22.35897436,  21.59487179],
        [ 56.04615385,  22.35897436,  21.59487179]]))
```

#### Diagnosis and Continent¶

In [37]:

```
def sparsify(dataframe, columns=[]):
    df = dataframe
   
    indices_df = pd.concat(
        [pd.Series(df.index.format(sparsify=True, adjoin=False)[df.index.names.index(lvl)], name=lvl)
         for lvl in df.index.names]
        , axis=1)
   
    series_lst = []
   
    for col in df.columns:
        if col in columns:
            sparsify_on_index_col = indices_df.columns[0]
            index_pos_true = indices_df[sparsify_on_index_col] == ''
            index_positions = index_pos_true[index_pos_true==True].index
            sparsified = df[col].reset_index(drop=True)
            sparsified.iloc[index_positions] = ''
            series_lst.append(sparsified)
        else:
            series_lst.append(df[col].reset_index(drop=True))
       
    cols_df = pd.concat(series_lst, axis=1)
   
    return indices_df.join(cols_df)
```

In [38]:

```
tbl1_crosstab = []

tbls_chisq_pvals = {}

tbl1_chisq_pvals = {}

cols1_lbls_dict = {}

cols1_lbls_dict['diagnosis'] = "Diagnosis"
cols1_lbls_dict['continent'] = "Continent"

tbl1_crosstabs_cols = ['diagnosis',
                     'continent']

for col in tbl1_crosstabs_cols:
    tbl_crosstab = crosstab_with_percentages(exp_genus.mapping_df, col, "microbiotype", axis=0, percent=100, decimal_places=1)
    
    tbl_chisq = chi2_contingency(crosstab_percentages(exp_genus.mapping_df, col, "microbiotype", axis=0, percent=100))
    tbl_chisq_pval = tbl_chisq[1]
    
    tbl1_crosstab.append(tbl_crosstab)
    
    tbl1_chisq_pvals[cols1_lbls_dict[col]] = tbl_chisq_pval
    tbls_chisq_pvals[cols1_lbls_dict[col]] = tbl_chisq_pval
    

tbl1_crosstab_allvars = pd.concat(tbl1_crosstab, keys=[cols1_lbls_dict[k] for k in tbl1_crosstabs_cols], names=['variable', 'value'])

tbl1_chisq_pval_df = pd.Series(tbl1_chisq_pvals, name="p value").apply(round, args=(3,)).to_frame()
tbl1_chisq_pval_df['p value'] = tbl1_chisq_pval_df['p value'].apply(lambda x: "< 0.001" if x==0 else str(x))
tbl1_chisq_pval_df.index.name = 'variable'

tbl1_crosstab_allvars_pvals = tbl1_crosstab_allvars.join(tbl1_chisq_pval_df).reset_index().set_index(['variable', "p value", 'value'])
tbl1_crosstab_allvars_pvals_reset = tbl1_crosstab_allvars_pvals.reset_index("p value")
tbl1_crosstab_allvars_pvals_reset = tbl1_crosstab_allvars_pvals_reset[['microbiotype_1', 'microbiotype_2', 'microbiotype_3', "p value"]]
```

In [39]:

```
sparsify(tbl1_crosstab_allvars_pvals_reset, columns='p value')
```

Out[39]:

|  | variable | value | microbiotype\_1 | microbiotype\_2 | microbiotype\_3 | p value |
| --- | --- | --- | --- | --- | --- | --- |
| 0 | Diagnosis | CRSsNP | 56 (56.6%) | 27 (27.3%) | 16 (16.2%) | 0.317 |
| 1 |  | CRSwNP | 85 (49.4%) | 48 (27.9%) | 39 (22.7%) |  |
| 2 |  | Control | 81 (58.3%) | 42 (30.2%) | 16 (11.5%) |  |
| 3 | Continent | Asia | 27 (69.2%) | 11 (28.2%) | 1 (2.6%) | < 0.001 |
| 4 |  | Australasia | 67 (61.5%) | 23 (21.1%) | 19 (17.4%) |  |
| 5 |  | Europe | 7 (18.4%) | 22 (57.9%) | 9 (23.7%) |  |
| 6 |  | North\_America | 89 (56.3%) | 43 (27.2%) | 26 (16.5%) |  |
| 7 |  | South\_America | 32 (48.5%) | 18 (27.3%) | 16 (24.2%) |  |

#### Other clinical covariates¶

In [40]:

```
tbl2_crosstab = []

tbl2_chisq_pvals = {}

cols2_lbls_dict = {}

cols2_lbls_dict['prev_surgery_group'] = "Primary surgery"
cols2_lbls_dict['asthma_english'] = "Asthma"
cols2_lbls_dict['aspirin_sensitivity_english'] = "Aspirin sensitivity" 
cols2_lbls_dict['diabetes_english'] = "Diabetes"
cols2_lbls_dict['gord_english'] = "GORD"
cols2_lbls_dict['smoker_current_english'] = "Current Smoker" 

tbl2_crosstabs_cols = ['asthma_english',
                     'aspirin_sensitivity_english',
                     'diabetes_english',
                     'gord_english',
                     'smoker_current_english',
                     'prev_surgery_group']

for col in tbl2_crosstabs_cols:
    tbl_crosstab = crosstab_with_percentages(exp_genus.mapping_df, col, "microbiotype", axis=0, percent=100, decimal_places=1)
    
    tbl_chisq = chi2_contingency(crosstab_percentages(exp_genus.mapping_df, col, "microbiotype", axis=0, percent=100))
    tbl_chisq_pval = tbl_chisq[1]
    
    tbl2_crosstab.append(tbl_crosstab)
    tbl2_chisq_pvals[cols2_lbls_dict[col]] = tbl_chisq_pval
    tbls_chisq_pvals[cols2_lbls_dict[col]] = tbl_chisq_pval
    

tbl2_crosstab_allvars = pd.concat(tbl2_crosstab, keys=[cols2_lbls_dict[k] for k in tbl2_crosstabs_cols], names=['variable', 'value'])

chisq_pval_df = pd.Series(tbls_chisq_pvals, name="p value").apply(round, args=(3,)).to_frame()
chisq_pval_df.index.name = 'variable'

tbl2_crosstab_allvars_pvals = tbl2_crosstab_allvars.join(chisq_pval_df).reset_index().set_index(['variable', "p value", 'value'])

tbl2_crosstab_allvars_pvals_reset = tbl2_crosstab_allvars_pvals.reset_index("p value")
tbl2_crosstab_allvars_pvals_reset = tbl2_crosstab_allvars_pvals_reset[['microbiotype_1', 'microbiotype_2', 'microbiotype_3', "p value"]]
```

In [41]:

```
sparsify(tbl2_crosstab_allvars_pvals_reset, columns=['p value'])
```

Out[41]:

|  | variable | value | microbiotype\_1 | microbiotype\_2 | microbiotype\_3 | p value |
| --- | --- | --- | --- | --- | --- | --- |
| 0 | Asthma | No | 162 (56.4%) | 81 (28.2%) | 44 (15.3%) | 0.679 |
| 1 |  | Yes | 55 (51.4%) | 31 (29.0%) | 21 (19.6%) |  |
| 2 | Aspirin sensitivity | No | 202 (55.3%) | 106 (29.0%) | 57 (15.6%) | 0.02 |
| 3 |  | Yes | 12 (48.0%) | 5 (20.0%) | 8 (32.0%) |  |
| 4 | Diabetes | No | 189 (54.9%) | 98 (28.5%) | 57 (16.6%) | 0.979 |
| 5 |  | Yes | 22 (55.0%) | 11 (27.5%) | 7 (17.5%) |  |
| 6 | GORD | No | 177 (55.3%) | 93 (29.1%) | 50 (15.6%) | 0.914 |
| 7 |  | Yes | 35 (55.6%) | 17 (27.0%) | 11 (17.5%) |  |
| 8 | Current Smoker | No | 204 (54.4%) | 110 (29.3%) | 61 (16.3%) | 0.029 |
| 9 |  | Yes | 15 (57.7%) | 4 (15.4%) | 7 (26.9%) |  |
| 10 | Primary surgery | No | 92 (47.2%) | 57 (29.2%) | 46 (23.6%) | 0.057 |
| 11 |  | Yes | 130 (60.5%) | 60 (27.9%) | 25 (11.6%) |  |

*P value corrections*

In [42]:

```
from statsmodels.stats.multitest import multipletests as statsmodels_multipletests

results['aspirin_chisq_pval'] = round(tbls_chisq_pvals['Aspirin sensitivity'], 3)

#correct p value using stats models
cc_corr_pvals = statsmodels_multipletests([p for p in tbls_chisq_pvals.values()], method="fdr_bh")[1]
cc_corrected_pvals_dict = {k:v for k,v in zip(tbls_chisq_pvals.keys(), cc_corr_pvals)}

tbl1_corrected_pvals_dict = {k:cc_corrected_pvals_dict[k] for k in cc_corrected_pvals_dict
                            if k in ['Diagnosis', 'Continent']}

tbl2_corrected_pvals_dict = {k:cc_corrected_pvals_dict[k] for k in cc_corrected_pvals_dict
                            if k not in ['Diagnosis', 'Continent']}

results['aspirin_chisq_pval_corrected'] = round(cc_corrected_pvals_dict['Aspirin sensitivity'], 3)


results['continent_chisq_pval_corrected'] = round(cc_corrected_pvals_dict['Continent'], 3)
if results['continent_chisq_pval_corrected'] == 0:
    results['continent_chisq_pval_corrected'] = 0.001 #round to 3 decimal places if < 0.001
```

In [43]:

```
#Rebuild table 1 with p value corrections 
for col in tbl1_crosstabs_cols:
    tbl_crosstab = crosstab_with_percentages(exp_genus.mapping_df, col, "microbiotype", axis=0, percent=100, decimal_places=1)
    
    tbl_chisq = chi2_contingency(crosstab_percentages(exp_genus.mapping_df, col, "microbiotype", axis=0, percent=100))
    tbl_chisq_pval = tbl_chisq[1]
    
    tbl1_crosstab.append(tbl_crosstab)

tbl1_crosstab_allvars = pd.concat(tbl1_crosstab, keys=[cols1_lbls_dict[k] for k in tbl1_crosstabs_cols], names=['variable', 'value'])

tbl1_chisq_pval_df = pd.Series(tbl1_corrected_pvals_dict, name="p value").apply(round, args=(3,)).to_frame()
tbl1_chisq_pval_df['p value'] = tbl1_chisq_pval_df['p value'].apply(lambda x: "< 0.001" if x==0 else str(x))
tbl1_chisq_pval_df.index.name = 'variable'

tbl1_crosstab_allvars_pvals = tbl1_crosstab_allvars.join(tbl1_chisq_pval_df).reset_index().set_index(['variable', "p value", 'value'])
tbl1_crosstab_allvars_pvals_reset = tbl1_crosstab_allvars_pvals.reset_index("p value")
tbl1_crosstab_allvars_pvals_reset = tbl1_crosstab_allvars_pvals_reset[['microbiotype_1', 'microbiotype_2', 'microbiotype_3', "p value"]]

sparsified_tbl1_crosstab = sparsify(tbl1_crosstab_allvars_pvals_reset, columns='p value')
display(sparsified_tbl1_crosstab)
sparsified_tbl1_crosstab.to_csv("./results/microbiotyping/tables/microbiotypes_diag_continent.tsv", sep="\t", index=0)
```

|  | variable | value | microbiotype\_1 | microbiotype\_2 | microbiotype\_3 | p value |
| --- | --- | --- | --- | --- | --- | --- |
| 0 | Diagnosis | CRSsNP | 56 (56.6%) | 27 (27.3%) | 16 (16.2%) | 0.507 |
| 1 |  | CRSwNP | 85 (49.4%) | 48 (27.9%) | 39 (22.7%) |  |
| 2 |  | Control | 81 (58.3%) | 42 (30.2%) | 16 (11.5%) |  |
| 3 | Continent | Asia | 27 (69.2%) | 11 (28.2%) | 1 (2.6%) | < 0.001 |
| 4 |  | Australasia | 67 (61.5%) | 23 (21.1%) | 19 (17.4%) |  |
| 5 |  | Europe | 7 (18.4%) | 22 (57.9%) | 9 (23.7%) |  |
| 6 |  | North\_America | 89 (56.3%) | 43 (27.2%) | 26 (16.5%) |  |
| 7 |  | South\_America | 32 (48.5%) | 18 (27.3%) | 16 (24.2%) |  |

In [44]:

```
#Rebuild table 2 with p value corrections 
tbl2_crosstab_allvars = pd.concat(tbl2_crosstab, keys=[cols2_lbls_dict[k] for k in tbl2_crosstabs_cols], names=['variable', 'value'])

chisq_pval_df = pd.Series(tbl2_corrected_pvals_dict, name="p value").apply(round, args=(3,)).to_frame()
chisq_pval_df.index.name = 'variable'

tbl2_crosstab_allvars_pvals = tbl2_crosstab_allvars.join(chisq_pval_df).reset_index().set_index(['variable', "p value", 'value'])

tbl2_crosstab_allvars_pvals_reset = tbl2_crosstab_allvars_pvals.reset_index("p value")
tbl2_crosstab_allvars_pvals_reset = tbl2_crosstab_allvars_pvals_reset[['microbiotype_1', 'microbiotype_2', 'microbiotype_3', "p value"]]

sparsified_tbl2_crosstab = sparsify(tbl2_crosstab_allvars_pvals_reset, columns='p value')
display(sparsified_tbl2_crosstab)
sparsified_tbl2_crosstab.to_csv("./results/microbiotyping/tables/microbiotypes_variables.tsv", sep="\t", index=0)
```

|  | variable | value | microbiotype\_1 | microbiotype\_2 | microbiotype\_3 | p value |
| --- | --- | --- | --- | --- | --- | --- |
| 0 | Asthma | No | 162 (56.4%) | 81 (28.2%) | 44 (15.3%) | 0.906 |
| 1 |  | Yes | 55 (51.4%) | 31 (29.0%) | 21 (19.6%) |  |
| 2 | Aspirin sensitivity | No | 202 (55.3%) | 106 (29.0%) | 57 (15.6%) | 0.077 |
| 3 |  | Yes | 12 (48.0%) | 5 (20.0%) | 8 (32.0%) |  |
| 4 | Diabetes | No | 189 (54.9%) | 98 (28.5%) | 57 (16.6%) | 0.979 |
| 5 |  | Yes | 22 (55.0%) | 11 (27.5%) | 7 (17.5%) |  |
| 6 | GORD | No | 177 (55.3%) | 93 (29.1%) | 50 (15.6%) | 0.979 |
| 7 |  | Yes | 35 (55.6%) | 17 (27.0%) | 11 (17.5%) |  |
| 8 | Current Smoker | No | 204 (54.4%) | 110 (29.3%) | 61 (16.3%) | 0.077 |
| 9 |  | Yes | 15 (57.7%) | 4 (15.4%) | 7 (26.9%) |  |
| 10 | Primary surgery | No | 92 (47.2%) | 57 (29.2%) | 46 (23.6%) | 0.114 |
| 11 |  | Yes | 130 (60.5%) | 60 (27.9%) | 25 (11.6%) |  |

# Validating microbiotyping on "DataSet Two": the unsupervised approach¶

### Describing Dataset Two¶

In [45]:

```
#non-rarefied dataset 2
exp_full_dataset2.describe()
```

```
Num samples: 129
Num observations: 2053
Total count: 256350
Table density (fraction of non-zero values): 0.012256595566329478

Counts/sample summary:
Min: 12.0
Max: 10241.0
Median: 1249.0
Mean: 1987.2093023255813
Std. dev.: 2143.7899073341564
Sample Metadata Categories: None
Observation Metadata Categories: None

Counts/sample detail:
5833-1895-ADT0A-CGGAGCCT-TCTCTCCG-L001       12
5854-1955-ADT0A-GGAGCTAC-CTAAGCCT-L001       13
5884-2052-ADT0A-GCGTAGTA-TCTCTCCG-L001       32
5641-1349-ACAN3-GTAGAGGA-ACTGCATA-L001       37
2564-2059-ADT0A-CCTAAGAC-CGTCTAAT-L001       42
5573-1038-ACAN3-AGGCAGAA-TATCCTCT-L001       44
5761-1586-ACAN3-GGACTCCT-TCTCTCCG-L001       47
5752-1571-ACAN3-TCCTGAGC-TCTCTCCG-L001       48
5588-1110-ACAN3-CGTACTAG-GTAAGGAG-L001       49
5887-2099-ADT0A-ACTGAGCG-TCTCTCCG-L001       55
5778-1652-ACAN3-GGACTCCT-CGTCTAAT-L001       61
5764-1593-ACAN3-CGAGGCTG-CTAAGCCT-L001       80
5825-1864-ADT0A-TAGCGCTC-CGTCTAAT-L001       97
5823-1854-ADT0A-TAGCGCTC-CTAAGCCT-L001      103
5561-981-ACAN3-CGAGGCTG-CTCTCTAT-L001       132
5651-1397-ACAN3-TCCTGAGC-AAGGAGTA-L001      134
5647-1365-ACAN3-ATCTCAGG-ACTGCATA-L001      169
5671-1429-ACAN3-GCTCATGA-CGTCTAAT-L001      173
5769-1614-ACAN3-GTAGAGGA-CGTCTAAT-L001      180
5612-1233-ACAN3-CGAGGCTG-AAGGAGTA-L001      192
5734-1531-ACAN3-GCTCATGA-CTAAGCCT-L001      202
5621-1280-ACAN3-ATCTCAGG-GTAAGGAG-L001      205
5569-1013-ACAN3-ATCTCAGG-CTCTCTAT-L001      218
5576-1061-ACAN3-AAGAGGCA-TATCCTCT-L001      222
5649-1379-ACAN3-AGGCAGAA-AAGGAGTA-L001      223
4822-1041-ACAN3-TCCTGAGC-TATCCTCT-L001      229
5652-1391-ACAN3-GGACTCCT-AAGGAGTA-L001      287
5563-990-ACAN3-CTCTCTAC-CTCTCTAT-L001       288
5855-1959-ADT0A-TGCAGCTA-AAGGAGTA-L001      338
5870-1998-ADT0A-TCGACGTC-AAGGAGTA-L001      364
5593-1145-ACAN3-TAGGCATG-GTAAGGAG-L001      364
5759-1579-ACAN3-TAGGCATG-TCTCTCCG-L001      365
5628-1295-ACAN3-TCCTGAGC-ACTGCATA-L001      431
5552-1374-ACAN3-TCCTGAGC-CTCTCTAT-L001      455
5861-1970-ADT0A-TACGCTGC-CGTCTAAT-L001      459
5835-1908-ADT0A-TAGCGCTC-TCTCTCCG-L001      489
5876-2007-ADT0A-CGATCAGT-CGTCTAAT-L001      499
5051-2113-ADT0A-ACTGAGCG-CGTCTAAT-L001      505
5824-1863-ADT0A-GGAGCTAC-TCTCTCCG-L001      544
5780-1645-ACAN3-AAGAGGCA-CTAAGCCT-L001      545
5591-1136-ACAN3-GGACTCCT-GTAAGGAG-L001      592
5646-1354-ACAN3-CGTACTAG-AAGGAGTA-L001      672
239-2018-ADT0A-TACGCTGC-CTAAGCCT-L001       674
5793-1726-ACAN3-CGAGGCTG-TCTCTCCG-L001      721
5717-1448-ACAN3-TAGGCATG-CGTCTAAT-L001      811
5839-1921-ADT0A-GGAGCTAC-CGTCTAAT-L001      827
5720-1451-ACAN3-CGTACTAG-TCTCTCCG-L001      858
5900-2086-ADT0A-TGCAGCTA-CTAAGCCT-L001      878
5819-1830-ADT0A-CGGAGCCT-CTAAGCCT-L001      888
5574-1042-ACAN3-GGACTCCT-TATCCTCT-L001      892
5834-1901-ADT0A-CGATCAGT-AAGGAGTA-L001      893
5708-1440-ACAN3-ATCTCAGG-CGTCTAAT-L001      953
5826-1876-ADT0A-TACGCTGC-TCTCTCCG-L001      954
5558-994-ACAN3-GGACTCCT-CTCTCTAT-L001       969
5571-1023-ACAN3-TAAGGCGA-TATCCTCT-L001      980
5897-2084-ADT0A-TGCAGCTA-CGTCTAAT-L001      998
5586-1130-ACAN3-GCTCATGA-TATCCTCT-L001     1000
5581-1100-ACAN3-TAAGGCGA-GTAAGGAG-L001     1069
5880-2017-ADT0A-CGATCAGT-CTAAGCCT-L001     1080
5589-1115-ACAN3-AGGCAGAA-GTAAGGAG-L001     1120
5575-1050-ACAN3-CGAGGCTG-TATCCTCT-L001     1151
5567-1007-ACAN3-GCTCATGA-CTCTCTAT-L001     1153
5543-921-ACAN3-TCCTGAGC-CTAAGCCT-L001      1175
5888-2051-ADT0A-GCGTAGTA-CGTCTAAT-L001     1176
5554-1373-ACAN3-AGGCAGAA-CTCTCTAT-L001     1249
5875-2009-ADT0A-CGGAGCCT-CGTCTAAT-L001     1291
5840-1917-ADT0A-GCGTAGTA-CTAAGCCT-L001     1315
5600-1193-ACAN3-GTAGAGGA-GTAAGGAG-L001     1320
5594-1151-ACAN3-CTCTCTAC-GTAAGGAG-L001     1338
5488-695-ACAN3-GTAGAGGA-AAGGAGTA-L001      1341
5863-1976-ADT0A-TCGACGTC-CTAAGCCT-L001     1388
5765-1597-ACAN3-CTCTCTAC-TCTCTCCG-L001     1389
3853-1824-ACAN3-AGGCAGAA-CGTCTAAT-L001     1453
5650-1392-ACAN3-TAGGCATG-AAGGAGTA-L001     1494
5645-1360-ACAN3-TAAGGCGA-AAGGAGTA-L001     1555
5812-1797-ADT0A-ATGCGCAG-CGTCTAAT-L001     1594
5894-2075-ADT0A-CCTAAGAC-AAGGAGTA-L001     1609
5653-1411-ACAN3-CTCTCTAC-AAGGAGTA-L001     1652
5525-872-ACAN3-TAAGGCGA-CTAAGCCT-L001      1716
5630-1302-ACAN3-TAGGCATG-ACTGCATA-L001     1717
5807-1772-ACAN3-GTAGAGGA-TCTCTCCG-L001     1727
5618-1268-ACAN3-CGTACTAG-ACTGCATA-L001     1736
5637-1326-ACAN3-AAGAGGCA-ACTGCATA-L001     1812
5733-1511-ACAN3-CTCTCTAC-CTAAGCCT-L001     1849
2911-2010-ACAN3-CTCTCTAC-CGTCTAAT-L001     1858
5829-1888-ADT0A-ACTGAGCG-CTAAGCCT-L001     1869
5885-2062-ADT0A-CCTAAGAC-TCTCTCCG-L001     2055
5634-1320-ACAN3-CGAGGCTG-ACTGCATA-L001     2122
5802-1745-ACAN3-GTAGAGGA-CTAAGCCT-L001     2122
5830-1889-ADT0A-ACTCGCTA-TCTCTCCG-L001     2254
5559-992-ACAN3-TAGGCATG-CTCTCTAT-L001      2307
5592-1139-ACAN3-TCCTGAGC-GTAAGGAG-L001     2337
5828-1887-ADT0A-ATGCGCAG-TCTCTCCG-L001     2364
5551-955-ACAN3-TAAGGCGA-CTCTCTAT-L001      2394
5728-1477-ACAN3-TAGGCATG-CTAAGCCT-L001     2532
5862-1973-ADT0A-ATGCGCAG-CTAAGCCT-L001     2558
5769-1604-ACAN3-AAGAGGCA-CGTCTAAT-L001     2629
5553-1372-ACAN3-CGTACTAG-CTCTCTAT-L001     2650
689-1048-ACAN3-TAGGCATG-TATCCTCT-L001      2753
3633-1316-ACAN3-CTCTCTAC-ACTGCATA-L001     3003
5596-1175-ACAN3-AAGAGGCA-GTAAGGAG-L001     3008
5536-902-ACAN3-AGGCAGAA-CTAAGCCT-L001      3069
5629-1296-ACAN3-GGACTCCT-ACTGCATA-L001     3117
1415-1855-ADT0A-ACTCGCTA-CGTCTAAT-L001     3138
5778-1643-ACAN3-TCCTGAGC-CGTCTAAT-L001     3511
5568-1002-ACAN3-GTAGAGGA-CTCTCTAT-L001     3615
5842-1929-ADT0A-TCGACGTC-CGTCTAAT-L001     4034
5562-986-ACAN3-AAGAGGCA-CTCTCTAT-L001      4101
5860-1971-ADT0A-CCTAAGAC-CTAAGCCT-L001     4395
5019-1241-ACAN3-TAAGGCGA-ACTGCATA-L001     4409
5857-1961-ACAN3-CGTACTAG-CGTCTAAT-L001     4520
5800-1748-ACAN3-TAAGGCGA-CGTCTAAT-L001     4787
5485-688-ACAN3-AAGAGGCA-AAGGAGTA-L001      4802
5640-1346-ACAN3-GCTCATGA-ACTGCATA-L001     4913
5827-1870-ADT0A-CGATCAGT-TCTCTCCG-L001     5190
5587-1131-ACAN3-ATCTCAGG-TATCCTCT-L001     5324
5723-1457-ACAN3-GGACTCCT-CTAAGCCT-L001     5741
5577-1064-ACAN3-GTAGAGGA-TATCCTCT-L001     5742
5595-1168-ACAN3-CGAGGCTG-GTAAGGAG-L001     5861
5866-1983-ADT0A-ACTCGCTA-CTAAGCCT-L001     6008
5524-867-ACAN3-ATCTCAGG-AAGGAGTA-L001      6091
4025-1053-ACAN3-CTCTCTAC-TATCCTCT-L001     6126
1631-1265-ACAN3-AGGCAGAA-ACTGCATA-L001     6623
5526-888-ACAN3-CGTACTAG-CTAAGCCT-L001      6638
5721-1452-ACAN3-AGGCAGAA-TCTCTCCG-L001     7524
5572-1025-ACAN3-CGTACTAG-TATCCTCT-L001     7702
5800-1738-ACAN3-ATCTCAGG-CTAAGCCT-L001     7903
5718-1449-ACAN3-TAAGGCGA-TCTCTCCG-L001     8449
5503-772-ACAN3-GCTCATGA-AAGGAGTA-L001     10241
```

In [46]:

```
#rarefied dataset 2
exp_dataset2.describe()
```

```
Num samples: 97
Num observations: 1454
Total count: 38800
Table density (fraction of non-zero values): 0.016265120038571165

Counts/sample summary:
Min: 400.0
Max: 400.0
Median: 400.0
Mean: 400.0
Std. dev.: 0.0
Sample Metadata Categories: None
Observation Metadata Categories: None

Counts/sample detail:
5645-1360-ACAN3-TAAGGCGA-AAGGAGTA-L001    400
1631-1265-ACAN3-AGGCAGAA-ACTGCATA-L001    400
5717-1448-ACAN3-TAGGCATG-CGTCTAAT-L001    400
5765-1597-ACAN3-CTCTCTAC-TCTCTCCG-L001    400
5800-1748-ACAN3-TAAGGCGA-CGTCTAAT-L001    400
5793-1726-ACAN3-CGAGGCTG-TCTCTCCG-L001    400
5653-1411-ACAN3-CTCTCTAC-AAGGAGTA-L001    400
5827-1870-ADT0A-CGATCAGT-TCTCTCCG-L001    400
5840-1917-ADT0A-GCGTAGTA-CTAAGCCT-L001    400
5618-1268-ACAN3-CGTACTAG-ACTGCATA-L001    400
3853-1824-ACAN3-AGGCAGAA-CGTCTAAT-L001    400
5553-1372-ACAN3-CGTACTAG-CTCTCTAT-L001    400
5778-1643-ACAN3-TCCTGAGC-CGTCTAAT-L001    400
5834-1901-ADT0A-CGATCAGT-AAGGAGTA-L001    400
3633-1316-ACAN3-CTCTCTAC-ACTGCATA-L001    400
5554-1373-ACAN3-AGGCAGAA-CTCTCTAT-L001    400
5581-1100-ACAN3-TAAGGCGA-GTAAGGAG-L001    400
5733-1511-ACAN3-CTCTCTAC-CTAAGCCT-L001    400
5720-1451-ACAN3-CGTACTAG-TCTCTCCG-L001    400
5019-1241-ACAN3-TAAGGCGA-ACTGCATA-L001    400
5574-1042-ACAN3-GGACTCCT-TATCCTCT-L001    400
5800-1738-ACAN3-ATCTCAGG-CTAAGCCT-L001    400
5894-2075-ADT0A-CCTAAGAC-AAGGAGTA-L001    400
5551-955-ACAN3-TAAGGCGA-CTCTCTAT-L001     400
5630-1302-ACAN3-TAGGCATG-ACTGCATA-L001    400
5780-1645-ACAN3-AAGAGGCA-CTAAGCCT-L001    400
5900-2086-ADT0A-TGCAGCTA-CTAAGCCT-L001    400
5835-1908-ADT0A-TAGCGCTC-TCTCTCCG-L001    400
5562-986-ACAN3-AAGAGGCA-CTCTCTAT-L001     400
5589-1115-ACAN3-AGGCAGAA-GTAAGGAG-L001    400
5721-1452-ACAN3-AGGCAGAA-TCTCTCCG-L001    400
5866-1983-ADT0A-ACTCGCTA-CTAAGCCT-L001    400
5888-2051-ADT0A-GCGTAGTA-CGTCTAAT-L001    400
5577-1064-ACAN3-GTAGAGGA-TATCCTCT-L001    400
5807-1772-ACAN3-GTAGAGGA-TCTCTCCG-L001    400
5897-2084-ADT0A-TGCAGCTA-CGTCTAAT-L001    400
5802-1745-ACAN3-GTAGAGGA-CTAAGCCT-L001    400
1415-1855-ADT0A-ACTCGCTA-CGTCTAAT-L001    400
5595-1168-ACAN3-CGAGGCTG-GTAAGGAG-L001    400
5728-1477-ACAN3-TAGGCATG-CTAAGCCT-L001    400
5876-2007-ADT0A-CGATCAGT-CGTCTAAT-L001    400
5524-867-ACAN3-ATCTCAGG-AAGGAGTA-L001     400
5558-994-ACAN3-GGACTCCT-CTCTCTAT-L001     400
5634-1320-ACAN3-CGAGGCTG-ACTGCATA-L001    400
5640-1346-ACAN3-GCTCATGA-ACTGCATA-L001    400
5600-1193-ACAN3-GTAGAGGA-GTAAGGAG-L001    400
5826-1876-ADT0A-TACGCTGC-TCTCTCCG-L001    400
5568-1002-ACAN3-GTAGAGGA-CTCTCTAT-L001    400
5842-1929-ADT0A-TCGACGTC-CGTCTAAT-L001    400
5723-1457-ACAN3-GGACTCCT-CTAAGCCT-L001    400
2911-2010-ACAN3-CTCTCTAC-CGTCTAAT-L001    400
5594-1151-ACAN3-CTCTCTAC-GTAAGGAG-L001    400
5829-1888-ADT0A-ACTGAGCG-CTAAGCCT-L001    400
5812-1797-ADT0A-ATGCGCAG-CGTCTAAT-L001    400
5862-1973-ADT0A-ATGCGCAG-CTAAGCCT-L001    400
5552-1374-ACAN3-TCCTGAGC-CTCTCTAT-L001    400
5628-1295-ACAN3-TCCTGAGC-ACTGCATA-L001    400
5830-1889-ADT0A-ACTCGCTA-TCTCTCCG-L001    400
5861-1970-ADT0A-TACGCTGC-CGTCTAAT-L001    400
5559-992-ACAN3-TAGGCATG-CTCTCTAT-L001     400
5051-2113-ADT0A-ACTGAGCG-CGTCTAAT-L001    400
5571-1023-ACAN3-TAAGGCGA-TATCCTCT-L001    400
5567-1007-ACAN3-GCTCATGA-CTCTCTAT-L001    400
5718-1449-ACAN3-TAAGGCGA-TCTCTCCG-L001    400
5769-1604-ACAN3-AAGAGGCA-CGTCTAAT-L001    400
5857-1961-ACAN3-CGTACTAG-CGTCTAAT-L001    400
5880-2017-ADT0A-CGATCAGT-CTAAGCCT-L001    400
5596-1175-ACAN3-AAGAGGCA-GTAAGGAG-L001    400
5885-2062-ADT0A-CCTAAGAC-TCTCTCCG-L001    400
5650-1392-ACAN3-TAGGCATG-AAGGAGTA-L001    400
5572-1025-ACAN3-CGTACTAG-TATCCTCT-L001    400
5819-1830-ADT0A-CGGAGCCT-CTAAGCCT-L001    400
5824-1863-ADT0A-GGAGCTAC-TCTCTCCG-L001    400
5488-695-ACAN3-GTAGAGGA-AAGGAGTA-L001     400
5525-872-ACAN3-TAAGGCGA-CTAAGCCT-L001     400
5526-888-ACAN3-CGTACTAG-CTAAGCCT-L001     400
4025-1053-ACAN3-CTCTCTAC-TATCCTCT-L001    400
5591-1136-ACAN3-GGACTCCT-GTAAGGAG-L001    400
5839-1921-ADT0A-GGAGCTAC-CGTCTAAT-L001    400
5646-1354-ACAN3-CGTACTAG-AAGGAGTA-L001    400
5543-921-ACAN3-TCCTGAGC-CTAAGCCT-L001     400
5629-1296-ACAN3-GGACTCCT-ACTGCATA-L001    400
5575-1050-ACAN3-CGAGGCTG-TATCCTCT-L001    400
5587-1131-ACAN3-ATCTCAGG-TATCCTCT-L001    400
5586-1130-ACAN3-GCTCATGA-TATCCTCT-L001    400
5875-2009-ADT0A-CGGAGCCT-CGTCTAAT-L001    400
5708-1440-ACAN3-ATCTCAGG-CGTCTAAT-L001    400
5592-1139-ACAN3-TCCTGAGC-GTAAGGAG-L001    400
689-1048-ACAN3-TAGGCATG-TATCCTCT-L001     400
5828-1887-ADT0A-ATGCGCAG-TCTCTCCG-L001    400
5536-902-ACAN3-AGGCAGAA-CTAAGCCT-L001     400
5485-688-ACAN3-AAGAGGCA-AAGGAGTA-L001     400
5503-772-ACAN3-GCTCATGA-AAGGAGTA-L001     400
5860-1971-ADT0A-CCTAAGAC-CTAAGCCT-L001    400
5637-1326-ACAN3-AAGAGGCA-ACTGCATA-L001    400
239-2018-ADT0A-TACGCTGC-CTAAGCCT-L001     400
5863-1976-ADT0A-TCGACGTC-CTAAGCCT-L001    400
```

In [47]:

```
results['n_dataset2_before_rarefaction'] = len(exp_full_dataset2.samples)

results['n_dataset2'] = len(exp_dataset2.samples)

results['n_dataset2_CRSsNP'] = exp_dataset2.mapping_df['diagnosis'].value_counts()['CRSsNP']
results['n_dataset2_CRSwNP'] = exp_dataset2.mapping_df['diagnosis'].value_counts()['CRSwNP']
results['n_dataset2_Control'] = exp_dataset2.mapping_df['diagnosis'].value_counts()['Control']
```

#### Taxonomic composition of Dataset Two samples¶

Here we also observe the dominance of *Corynebacterium* and *Staphylococcus*.

In [48]:

```
clean_taxa_in_index(exp_genus_dataset2.apply(AbundancePrevalenceStatistics()).data_df.head(10))
```

Out[48]:

|  | mean\_relative\_abundance | prevalence |
| --- | --- | --- |
| genus |  |  |
| Corynebacterium | 28.551546 | 68.041237 |
| Staphylococcus | 26.917526 | 77.319588 |
| Haemophilus | 10.113402 | 23.711340 |
| Moraxella | 4.971649 | 11.340206 |
| Streptococcus | 4.332474 | 23.711340 |
| unidentified (Enterobacteriaceae) | 3.536082 | 9.278351 |
| Alloiococcus | 2.989691 | 12.371134 |
| Fusobacterium | 2.827320 | 17.525773 |
| Pseudomonas | 2.260309 | 10.309278 |
| unidentified (Neisseriaceae) | 2.023196 | 10.309278 |

In [49]:

```
from omicexperiment.transforms.observation import TopAbundantObservations
exp_genus_dataset2_top10 = exp_genus_dataset2.apply(TopAbundantObservations(10)).to_relative_abundance()
exp_genus_dataset2_top10.groupby("diagnosis").plot(backend="matplotlib")
pyplot.show()
```

Similar to what we reported in our manuscript Paramasivan et al., in this dataset there also appears to be a lower relative abundance of Corynebacteria in CRSwNP compared to controls.

### Performing the microbiotyping methodology on Dataset Two samples¶

In [50]:

```
#Compute JSD
dataset_2_counts_df = exp_genus_dataset2.data_df
%R -i dataset_2_counts_df
%R library(philentropy)
%R dataset2_jsd_dist <- as.matrix(philentropy::distance(t(dataset_2_counts_df/100), "jensen-shannon"))
%R -o dataset2_jsd_dist

dataset2_jsd_dist = pd.DataFrame(dataset2_jsd_dist, index=dataset_2_counts_df.columns, columns=dataset_2_counts_df.columns)


#Perform PCoA
dataset2_beta_div_exp = exp_genus_dataset2.with_data_df(dataset2_jsd_dist)
dataset2_dist_matrix_pcoa = dataset2_beta_div_exp.apply(PCoA)
dataset2_three_PCs_df = dataset2_dist_matrix_pcoa.data_df.T[['PC1', 'PC2', 'PC3']]
dataset2_three_PCs_df_with_metadata = dataset2_three_PCs_df.join(exp_genus_dataset2.mapping_df[['dataset']])

# Perform K-Means
from sklearn.cluster import KMeans
dataset2_kmeans = KMeans(n_clusters=3, random_state=0).fit(dataset2_three_PCs_df[['PC1', 'PC2']])

dataset2_clusters = pd.Series(["cluster_" + str(x + 1) for x in dataset2_kmeans.labels_],
                           index=dataset2_three_PCs_df.index)

exp_genus_dataset2.mapping_df['cluster'] = dataset2_clusters
dataset2_three_PCs_df_with_metadata = dataset2_three_PCs_df_with_metadata.join(exp_genus_dataset2.mapping_df[['cluster']])

#relate clusters to the original microbiotypes
dataset2_clusters_df = exp_genus_dataset2.groupby('cluster').data_df

corynebacterium_dominated_cluster = dataset2_clusters_df.T.sort_values("g__Corynebacterium", ascending=False).index[0]
staph_dominated_cluster = dataset2_clusters_df.T.sort_values("g__Staphylococcus", ascending=False).index[0]
haemophilus_max_cluster = dataset2_clusters_df.T.sort_values("g__Streptococcus", ascending=False).index[0]

microbiotype_replace_dict = {corynebacterium_dominated_cluster: 'microbiotype_1',
                            staph_dominated_cluster: 'microbiotype_2',
                            haemophilus_max_cluster: 'microbiotype_3'}

dataset2_three_PCs_df_with_metadata['microbiotype'] = exp_genus_dataset2.mapping_df['cluster'].replace(microbiotype_replace_dict)
exp_genus_dataset2.mapping_df['microbiotype'] = exp_genus_dataset2.mapping_df['cluster'].replace(microbiotype_replace_dict)
```

```
/home/ahmed/dev/biomenv3/lib/python3.6/site-packages/rpy2/rinterface/__init__.py:185: RRuntimeWarning: Metric: 'jensen-shannon' using unit: 'log'.

  warnings.warn(x, RRuntimeWarning)
/home/ahmed/dev/biomenv3/lib/python3.6/site-packages/skbio/stats/ordination/_principal_coordinate_analysis.py:111: RuntimeWarning: The result contains negative eigenvalues. Please compare their magnitude with the magnitude of some of the largest positive eigenvalues. If the negative ones are smaller, it's probably safe to ignore them, but if they are large in magnitude, the results won't be useful. See the Notes section for more details. The smallest eigenvalue is -10.17309946948783 and the largest is 68.41189832433243.
  RuntimeWarning
```

In [51]:

```
exp_genus_dataset2_top10.to_relative_abundance().groupby('cluster').plot(backend="matplotlib")

import seaborn
seaborn.lmplot('PC1', 'PC2', hue='cluster', fit_reg=False,
               data=dataset2_three_PCs_df_with_metadata)
```

Out[51]:

```
<seaborn.axisgrid.FacetGrid at 0x7fa4607119e8>
```

And that is the composition of the microbiotypes using this unsupervised approach..

In [52]:

```
clean_taxa_in_index(exp_genus_dataset2_top10.groupby("microbiotype").data_df)
```

Out[52]:

| microbiotype | microbiotype\_1 | microbiotype\_2 | microbiotype\_3 |
| --- | --- | --- | --- |
| Other | 11.559211 | 7.528846 | 14.492424 |
| Alloiococcus | 7.565789 | 0.000000 | 0.075758 |
| Corynebacterium | 62.144737 | 11.894231 | 2.992424 |
| Fusobacterium | 0.335526 | 1.932692 | 6.401515 |
| Haemophilus | 0.118421 | 0.028846 | 29.568182 |
| Moraxella | 1.664474 | 0.000000 | 12.696970 |
| Pseudomonas | 0.250000 | 0.461538 | 5.992424 |
| Staphylococcus | 10.513158 | 77.875000 | 5.659091 |
| Streptococcus | 1.098684 | 0.173077 | 11.333333 |
| unidentified (Enterobacteriaceae) | 2.440789 | 0.057692 | 7.537879 |
| unidentified (Neisseriaceae) | 2.309211 | 0.048077 | 3.250000 |

Results are similar to the original microbiotypes!  
Let us look at prevalence..

In [53]:

```
dataset2_microbiotypes_prevalence_df = exp_genus_dataset2.mapping_df['microbiotype'].value_counts() / \
                                        exp_genus_dataset2.mapping_df['microbiotype'].value_counts().sum() * 100

dataset2_microbiotypes_prevalence_df = dataset2_microbiotypes_prevalence_df.apply(round, args=(2,))

display(dataset2_microbiotypes_prevalence_df)

results['n_dataset2_microbiotype1_percent'] = round(dataset2_microbiotypes_prevalence_df['microbiotype_1'], 1)
results['n_dataset2_microbiotype2_percent'] = round(dataset2_microbiotypes_prevalence_df['microbiotype_2'], 1)
results['n_dataset2_microbiotype3_percent'] = round(dataset2_microbiotypes_prevalence_df['microbiotype_3'], 1)
```

```
microbiotype_1    39.18
microbiotype_3    34.02
microbiotype_2    26.80
Name: microbiotype, dtype: float64
```

Again in this dataset "Dataset Two", it appears that the Corynebacterium-dominated microbiotype is most prevalent, similar to what was found in the Main Dataset.

We thus demonstrated on a separate dataset ("Dataset Two"), the reproduction of our microbiotyping methodology in an **unsupervised approach**.

# Validating microbiotyping on "DataSet Two": the semi-supervised approach¶

##### Combine the two genus-level OTU tables¶

In [54]:

```
#combine counts
combined_data_df = pd.concat([exp_genus.to_relative_abundance().data_df,
                              exp_genus_dataset2.to_relative_abundance().data_df],
                             axis=1, verify_integrity=True).fillna(0)

        
#combine metadata
combined_mapping_df = pd.concat([exp_genus.mapping_df,exp_genus_dataset2.mapping_df],
                                 axis=0)
combined_mapping_columns_to_include = ['dataset', 'diagnosis']
combined_mapping_df = combined_mapping_df[combined_mapping_columns_to_include]

#combined OmicExperiment object
combined_exp = MicrobiomeExperiment(combined_data_df, combined_mapping_df)

results["n_combined_datasets"] = len(combined_exp.samples)

combined_exp.describe()
```

```
Num samples: 507
Num observations: 228
Total count: 50700.0
Table density (fraction of non-zero values): 0.020701408353230217

Counts/sample summary:
Min: 100.0
Max: 100.0
Median: 100.0
Mean: 100.0
Std. dev.: 0.0
Sample Metadata Categories: None
Observation Metadata Categories: None

Counts/sample detail:
AF18-16S-AR34D-TCGACGTC-GCGTAAGA          100.0
AU17-16S-B39FG-CGTACTAG-CTAAGCCT          100.0
AH15-16S-ARRDR-TGCAGCTA-CTCTCTAT          100.0
AX15-16S-B3RMH-CCTAAGAC-TTCTAGCT          100.0
AH30-16S-ARRDR-GGAGCTAC-GTAAGGAG          100.0
AB18-16S-ATC1H-TCGACGTC-TATCCTCT          100.0
AE17-16S-AR34D-TACGCTGC-TTCTAGCT          100.0
AA30-16S-ATC1H-TACGCTGC-CTCTCTAT          100.0
AA15-16S-ARW7H-GCTCATGA-GAGCCTTA          100.0
AM18-16S-AT526-GCTCATGA-AAGGCTAT          100.0
AS08-16S-B39FG-GTAGAGGA-CTCTCTAT          100.0
AX12-16S-B3RMH-ATGCGCAG-TTCTAGCT          100.0
AS20-16S-B39FG-GTAGAGGA-TATCCTCT          100.0
AN10-16S-AT526-AAGAGGCA-TTATGCGA          100.0
AH03-16S-AR34D-CCTAAGAC-TTATGCGA          100.0
AC16-16S-ATC1H-CGGAGCCT-AAGGAGTA          100.0
AK22-16S-AT526-AGGCAGAA-TTCTAGCT          100.0
AH13-16S-ARRDR-CCTAAGAC-CTCTCTAT          100.0
AA04-16S-ARW7H-ATCTCAGG-AAGGCTAT          100.0
AU05-16S-B39FG-CGTACTAG-AAGGAGTA          100.0
AP18-16S-AT62V-CGGAGCCT-CCTAGAGT          100.0
AF25-16S-AR34D-TAGCGCTC-CTATTAAG          100.0
AK11-16S-AT526-TCCTGAGC-TCGACTAG          100.0
AQ24-16S-AT62V-GCGTAGTA-AAGGCTAT          100.0
AN14-16S-AT62V-CGGAGCCT-TCGACTAG          100.0
AA11-16S-ARW7H-CTCTCTAC-GAGCCTTA          100.0
AW17-16S-B39FG-GTAGAGGA-CGTCTAAT          100.0
AW22-16S-B39FG-AGGCAGAA-TCTCTCCG          100.0
AB10-16S-ATC1H-CGGAGCCT-TATCCTCT          100.0
AD25-16S-ATC1H-ATGCGCAG-TCTCTCCG          100.0
AB26-16S-ATC1H-ACTGAGCG-GTAAGGAG          100.0
AW13-16S-B39FG-TAGGCATG-CGTCTAAT          100.0
AW06-16S-B39FG-GCTCATGA-CTAAGCCT          100.0
AP02-16S-AT62V-TCGACGTC-TCGACTAG          100.0
AI23-16S-ARRDR-ACTCGCTA-AAGGAGTA          100.0
AP30-16S-AT62V-GCGTAGTA-GCGTAAGA          100.0
AI10-16S-ARRDR-TCGACGTC-GTAAGGAG          100.0
AB19-16S-ATC1H-ACTCGCTA-GTAAGGAG          100.0
AE27-16S-AR34D-GCGTAGTA-CCTAGAGT          100.0
AI22-16S-ARRDR-TCGACGTC-ACTGCATA          100.0
AJ09-16S-ARRDR-TACGCTGC-CTAAGCCT          100.0
AD18-16S-ATC1H-TGCAGCTA-CGTCTAAT          100.0
AB06-16S-ATC1H-TCGACGTC-CTCTCTAT          100.0
AH09-16S-ARRDR-TACGCTGC-CTCTCTAT          100.0
AP12-16S-AT62V-CGATCAGT-TTCTAGCT          100.0
AQ20-16S-AT62V-TGCAGCTA-CTATTAAG          100.0
AH16-16S-ARRDR-TCGACGTC-CTCTCTAT          100.0
AI18-16S-ARRDR-ACTGAGCG-ACTGCATA          100.0
AA05-16S-ARW7H-TAAGGCGA-GAGCCTTA          100.0
AM16-16S-AT526-AAGAGGCA-AAGGCTAT          100.0
AT11-16S-B39FG-TAGGCATG-ACTGCATA          100.0
AS29-16S-B39FG-TAGGCATG-GTAAGGAG          100.0
AQ08-16S-AT62V-TGCAGCTA-GCGTAAGA          100.0
AP01-16S-AT62V-TGCAGCTA-TCGACTAG          100.0
AJ20-16S-ARRDR-CGGAGCCT-CGTCTAAT          100.0
AA02-16S-ARW7H-GTAGAGGA-AAGGCTAT          100.0
AL07-16S-AT526-TAGGCATG-CCTAGAGT          100.0
AX11-16S-B3RMH-TACGCTGC-TTCTAGCT          100.0
AH20-16S-ARRDR-CGGAGCCT-TATCCTCT          100.0
AC12-16S-ATC1H-TCGACGTC-ACTGCATA          100.0
AH17-16S-ARRDR-ACTCGCTA-TATCCTCT          100.0
AM01-16S-AT526-TAGGCATG-CTATTAAG          100.0
AX16-16S-B3RMH-CGATCAGT-TTCTAGCT          100.0
AI05-16S-ARRDR-TAGCGCTC-GTAAGGAG          100.0
AJ13-16S-ARRDR-CCTAAGAC-CTAAGCCT          100.0
AJ14-16S-ARRDR-CGATCAGT-CTAAGCCT          100.0
AS21-16S-B39FG-GCTCATGA-TATCCTCT          100.0
AB07-16S-ATC1H-ACTCGCTA-TATCCTCT          100.0
AS02-16S-B39FG-TCCTGAGC-CTCTCTAT          100.0
AI13-16S-ARRDR-GCGTAGTA-ACTGCATA          100.0
AM14-16S-AT526-CTCTCTAC-AAGGCTAT          100.0
AL24-16S-AT526-GCTCATGA-GCGTAAGA          100.0
AC19-16S-ATC1H-TAGCGCTC-AAGGAGTA          100.0
AH05-16S-AR34D-TGCAGCTA-TTATGCGA          100.0
AJ06-16S-ARRDR-GGAGCTAC-CTAAGCCT          100.0
AS06-16S-B39FG-CGAGGCTG-CTCTCTAT          100.0
AX19-16S-B3RMH-ACTCGCTA-CCTAGAGT          100.0
AS27-16S-B39FG-TCCTGAGC-GTAAGGAG          100.0
AE24-16S-AR34D-TCGACGTC-TTCTAGCT          100.0
AK08-16S-ARRDR-CGATCAGT-TCTCTCCG          100.0
AG07-16S-AR34D-TAGCGCTC-AAGGCTAT          100.0
AG23-16S-AR34D-TGCAGCTA-GAGCCTTA          100.0
AA25-16S-ARW7H-AAGAGGCA-TTATGCGA          100.0
AL08-16S-AT526-CTCTCTAC-CCTAGAGT          100.0
AB22-16S-ATC1H-CGGAGCCT-GTAAGGAG          100.0
AQ15-16S-AT62V-ATGCGCAG-CTATTAAG          100.0
AC27-16S-ATC1H-GCGTAGTA-CTAAGCCT          100.0
AK21-16S-AT526-CGTACTAG-TTCTAGCT          100.0
AW15-16S-B39FG-CGAGGCTG-CGTCTAAT          100.0
AS22-16S-B39FG-ATCTCAGG-TATCCTCT          100.0
AK04-16S-ARRDR-ATGCGCAG-TCTCTCCG          100.0
AM23-16S-AT526-TCCTGAGC-GAGCCTTA          100.0
AH07-16S-ARRDR-GCGTAGTA-CTCTCTAT          100.0
AD13-16S-ATC1H-ATGCGCAG-CGTCTAAT          100.0
AM30-16S-AT526-GCTCATGA-GAGCCTTA          100.0
AM05-16S-AT526-GTAGAGGA-CTATTAAG          100.0
AP17-16S-AT62V-GCGTAGTA-CCTAGAGT          100.0
AM03-16S-AT526-CGAGGCTG-CTATTAAG          100.0
AU15-16S-B39FG-ATCTCAGG-AAGGAGTA          100.0
AW01-16S-B39FG-TAGGCATG-CTAAGCCT          100.0
AG27-16S-AR34D-GCGTAGTA-TTATGCGA          100.0
AJ29-16S-ARRDR-ACTCGCTA-TCTCTCCG          100.0
AQ04-16S-AT62V-TAGCGCTC-GCGTAAGA          100.0
AA17-16S-ARW7H-TAAGGCGA-TTATGCGA          100.0
AA13-16S-ARW7H-AAGAGGCA-GAGCCTTA          100.0
AM07-16S-AT526-ATCTCAGG-CTATTAAG          100.0
AK13-16S-AT526-TAGGCATG-TCGACTAG          100.0
AU08-16S-B39FG-GGACTCCT-AAGGAGTA          100.0
AS11-16S-B39FG-TAAGGCGA-TATCCTCT          100.0
AS10-16S-B39FG-ATCTCAGG-CTCTCTAT          100.0
AH12-16S-ARRDR-ACTGAGCG-CTCTCTAT          100.0
AX10-16S-B3RMH-CGGAGCCT-TTCTAGCT          100.0
AI06-16S-ARRDR-ACTGAGCG-GTAAGGAG          100.0
AX18-16S-B3RMH-TCGACGTC-TTCTAGCT          100.0
AH26-16S-ARRDR-CGATCAGT-TATCCTCT          100.0
AB23-16S-ATC1H-TACGCTGC-GTAAGGAG          100.0
AC07-16S-ATC1H-TAGCGCTC-ACTGCATA          100.0
AH01-16S-AR34D-TAGCGCTC-TTATGCGA          100.0
AK14-16S-AT526-CTCTCTAC-TCGACTAG          100.0
AT09-16S-B39FG-TCCTGAGC-ACTGCATA          100.0
AW20-16S-B39FG-TAAGGCGA-TCTCTCCG          100.0
AA12-16S-ARW7H-CGAGGCTG-GAGCCTTA          100.0
AH02-16S-AR34D-ACTGAGCG-TTATGCGA          100.0
AM11-16S-AT526-TCCTGAGC-AAGGCTAT          100.0
AN01-16S-AT526-ATCTCAGG-GAGCCTTA          100.0
AJ18-16S-ARRDR-GGAGCTAC-CGTCTAAT          100.0
AH28-16S-ARRDR-TCGACGTC-TATCCTCT          100.0
AJ05-16S-ARRDR-ACTCGCTA-CTAAGCCT          100.0
5840-1917-ADT0A-GCGTAGTA-CTAAGCCT-L001    100.0
5618-1268-ACAN3-CGTACTAG-ACTGCATA-L001    100.0
5553-1372-ACAN3-CGTACTAG-CTCTCTAT-L001    100.0
5800-1738-ACAN3-ATCTCAGG-CTAAGCCT-L001    100.0
5778-1643-ACAN3-TCCTGAGC-CGTCTAAT-L001    100.0
5834-1901-ADT0A-CGATCAGT-AAGGAGTA-L001    100.0
3633-1316-ACAN3-CTCTCTAC-ACTGCATA-L001    100.0
5554-1373-ACAN3-AGGCAGAA-CTCTCTAT-L001    100.0
5581-1100-ACAN3-TAAGGCGA-GTAAGGAG-L001    100.0
5733-1511-ACAN3-CTCTCTAC-CTAAGCCT-L001    100.0
5720-1451-ACAN3-CGTACTAG-TCTCTCCG-L001    100.0
5019-1241-ACAN3-TAAGGCGA-ACTGCATA-L001    100.0
5574-1042-ACAN3-GGACTCCT-TATCCTCT-L001    100.0
5826-1876-ADT0A-TACGCTGC-TCTCTCCG-L001    100.0
5842-1929-ADT0A-TCGACGTC-CGTCTAAT-L001    100.0
239-2018-ADT0A-TACGCTGC-CTAAGCCT-L001     100.0
5723-1457-ACAN3-GGACTCCT-CTAAGCCT-L001    100.0
5525-872-ACAN3-TAAGGCGA-CTAAGCCT-L001     100.0
5526-888-ACAN3-CGTACTAG-CTAAGCCT-L001     100.0
4025-1053-ACAN3-CTCTCTAC-TATCCTCT-L001    100.0
5591-1136-ACAN3-GGACTCCT-GTAAGGAG-L001    100.0
5839-1921-ADT0A-GGAGCTAC-CGTCTAAT-L001    100.0
5646-1354-ACAN3-CGTACTAG-AAGGAGTA-L001    100.0
5543-921-ACAN3-TCCTGAGC-CTAAGCCT-L001     100.0
5629-1296-ACAN3-GGACTCCT-ACTGCATA-L001    100.0
5575-1050-ACAN3-CGAGGCTG-TATCCTCT-L001    100.0
5587-1131-ACAN3-ATCTCAGG-TATCCTCT-L001    100.0
5827-1870-ADT0A-CGATCAGT-TCTCTCCG-L001    100.0
5653-1411-ACAN3-CTCTCTAC-AAGGAGTA-L001    100.0
5793-1726-ACAN3-CGAGGCTG-TCTCTCCG-L001    100.0
5800-1748-ACAN3-TAAGGCGA-CGTCTAAT-L001    100.0
5780-1645-ACAN3-AAGAGGCA-CTAAGCCT-L001    100.0
5900-2086-ADT0A-TGCAGCTA-CTAAGCCT-L001    100.0
5835-1908-ADT0A-TAGCGCTC-TCTCTCCG-L001    100.0
5562-986-ACAN3-AAGAGGCA-CTCTCTAT-L001     100.0
5589-1115-ACAN3-AGGCAGAA-GTAAGGAG-L001    100.0
5721-1452-ACAN3-AGGCAGAA-TCTCTCCG-L001    100.0
5866-1983-ADT0A-ACTCGCTA-CTAAGCCT-L001    100.0
5888-2051-ADT0A-GCGTAGTA-CGTCTAAT-L001    100.0
5577-1064-ACAN3-GTAGAGGA-TATCCTCT-L001    100.0
5807-1772-ACAN3-GTAGAGGA-TCTCTCCG-L001    100.0
5897-2084-ADT0A-TGCAGCTA-CGTCTAAT-L001    100.0
5802-1745-ACAN3-GTAGAGGA-CTAAGCCT-L001    100.0
1415-1855-ADT0A-ACTCGCTA-CGTCTAAT-L001    100.0
5586-1130-ACAN3-GCTCATGA-TATCCTCT-L001    100.0
5595-1168-ACAN3-CGAGGCTG-GTAAGGAG-L001    100.0
5876-2007-ADT0A-CGATCAGT-CGTCTAAT-L001    100.0
5524-867-ACAN3-ATCTCAGG-AAGGAGTA-L001     100.0
5558-994-ACAN3-GGACTCCT-CTCTCTAT-L001     100.0
5634-1320-ACAN3-CGAGGCTG-ACTGCATA-L001    100.0
5640-1346-ACAN3-GCTCATGA-ACTGCATA-L001    100.0
5600-1193-ACAN3-GTAGAGGA-GTAAGGAG-L001    100.0
5630-1302-ACAN3-TAGGCATG-ACTGCATA-L001    100.0
5551-955-ACAN3-TAAGGCGA-CTCTCTAT-L001     100.0
5894-2075-ADT0A-CCTAAGAC-AAGGAGTA-L001    100.0
3853-1824-ACAN3-AGGCAGAA-CGTCTAAT-L001    100.0
1631-1265-ACAN3-AGGCAGAA-ACTGCATA-L001    100.0
5717-1448-ACAN3-TAGGCATG-CGTCTAAT-L001    100.0
5765-1597-ACAN3-CTCTCTAC-TCTCTCCG-L001    100.0
5728-1477-ACAN3-TAGGCATG-CTAAGCCT-L001    100.0
5875-2009-ADT0A-CGGAGCCT-CGTCTAAT-L001    100.0
5708-1440-ACAN3-ATCTCAGG-CGTCTAAT-L001    100.0
5592-1139-ACAN3-TCCTGAGC-GTAAGGAG-L001    100.0
AL11-16S-AT526-GTAGAGGA-CCTAGAGT          100.0
AE06-16S-AR34D-TAGCGCTC-TCGACTAG          100.0
AB02-16S-ATC1H-ACTGAGCG-CTCTCTAT          100.0
AA14-16S-ARW7H-GTAGAGGA-GAGCCTTA          100.0
AQ17-16S-AT62V-ACTGAGCG-CTATTAAG          100.0
AW25-16S-B39FG-TAGGCATG-TCTCTCCG          100.0
AL27-16S-AT526-CGTACTAG-CTATTAAG          100.0
AB21-16S-ATC1H-GCGTAGTA-GTAAGGAG          100.0
AS09-16S-B39FG-GCTCATGA-CTCTCTAT          100.0
AG25-16S-AR34D-ACTCGCTA-TTATGCGA          100.0
AS13-16S-B39FG-AGGCAGAA-TATCCTCT          100.0
AP22-16S-AT62V-TAGCGCTC-CCTAGAGT          100.0
AU14-16S-B39FG-GCTCATGA-AAGGAGTA          100.0
5645-1360-ACAN3-TAAGGCGA-AAGGAGTA-L001    100.0
AS23-16S-B39FG-TAAGGCGA-GTAAGGAG          100.0
AP28-16S-AT62V-ACTCGCTA-GCGTAAGA          100.0
AJ01-16S-ARRDR-CCTAAGAC-AAGGAGTA          100.0
AQ18-16S-AT62V-CCTAAGAC-CTATTAAG          100.0
AN19-16S-AT62V-CCTAAGAC-TCGACTAG          100.0
AF12-16S-AR34D-ATGCGCAG-GCGTAAGA          100.0
AK16-16S-AT526-AAGAGGCA-TCGACTAG          100.0
AK30-16S-AT526-GCTCATGA-TTCTAGCT          100.0
AA16-16S-ARW7H-ATCTCAGG-GAGCCTTA          100.0
AG20-16S-AR34D-ACTGAGCG-GAGCCTTA          100.0
AJ23-16S-ARRDR-TAGCGCTC-CGTCTAAT          100.0
AU01-16S-B39FG-GTAGAGGA-ACTGCATA          100.0
AJ04-16S-ARRDR-TCGACGTC-AAGGAGTA          100.0
AH04-16S-AR34D-CGATCAGT-TTATGCGA          100.0
AK06-16S-ARRDR-ACTGAGCG-TCTCTCCG          100.0
AF15-16S-AR34D-CCTAAGAC-GCGTAAGA          100.0
5650-1392-ACAN3-TAGGCATG-AAGGAGTA-L001    100.0
5596-1175-ACAN3-AAGAGGCA-GTAAGGAG-L001    100.0
689-1048-ACAN3-TAGGCATG-TATCCTCT-L001     100.0
5828-1887-ADT0A-ATGCGCAG-TCTCTCCG-L001    100.0
5536-902-ACAN3-AGGCAGAA-CTAAGCCT-L001     100.0
5485-688-ACAN3-AAGAGGCA-AAGGAGTA-L001     100.0
5503-772-ACAN3-GCTCATGA-AAGGAGTA-L001     100.0
5860-1971-ADT0A-CCTAAGAC-CTAAGCCT-L001    100.0
5637-1326-ACAN3-AAGAGGCA-ACTGCATA-L001    100.0
5488-695-ACAN3-GTAGAGGA-AAGGAGTA-L001     100.0
5824-1863-ADT0A-GGAGCTAC-TCTCTCCG-L001    100.0
5819-1830-ADT0A-CGGAGCCT-CTAAGCCT-L001    100.0
5559-992-ACAN3-TAGGCATG-CTCTCTAT-L001     100.0
2911-2010-ACAN3-CTCTCTAC-CGTCTAAT-L001    100.0
5594-1151-ACAN3-CTCTCTAC-GTAAGGAG-L001    100.0
5885-2062-ADT0A-CCTAAGAC-TCTCTCCG-L001    100.0
5829-1888-ADT0A-ACTGAGCG-CTAAGCCT-L001    100.0
5862-1973-ADT0A-ATGCGCAG-CTAAGCCT-L001    100.0
5552-1374-ACAN3-TCCTGAGC-CTCTCTAT-L001    100.0
5628-1295-ACAN3-TCCTGAGC-ACTGCATA-L001    100.0
5830-1889-ADT0A-ACTCGCTA-TCTCTCCG-L001    100.0
5861-1970-ADT0A-TACGCTGC-CGTCTAAT-L001    100.0
5051-2113-ADT0A-ACTGAGCG-CGTCTAAT-L001    100.0
5572-1025-ACAN3-CGTACTAG-TATCCTCT-L001    100.0
5571-1023-ACAN3-TAAGGCGA-TATCCTCT-L001    100.0
5567-1007-ACAN3-GCTCATGA-CTCTCTAT-L001    100.0
5718-1449-ACAN3-TAAGGCGA-TCTCTCCG-L001    100.0
5769-1604-ACAN3-AAGAGGCA-CGTCTAAT-L001    100.0
5857-1961-ACAN3-CGTACTAG-CGTCTAAT-L001    100.0
5880-2017-ADT0A-CGATCAGT-CTAAGCCT-L001    100.0
5812-1797-ADT0A-ATGCGCAG-CGTCTAAT-L001    100.0
5568-1002-ACAN3-GTAGAGGA-CTCTCTAT-L001    100.0
AL18-16S-AT526-GGACTCCT-GCGTAAGA          100.0
AN07-16S-AT526-TAGGCATG-TTATGCGA          100.0
AU12-16S-B39FG-AAGAGGCA-AAGGAGTA          100.0
AH27-16S-ARRDR-TGCAGCTA-TATCCTCT          100.0
AS04-16S-B39FG-TAGGCATG-CTCTCTAT          100.0
AK09-16S-ARRDR-TGCAGCTA-TCTCTCCG          100.0
AW29-16S-B3RMH-TACGCTGC-TCGACTAG          100.0
AH06-16S-AR34D-TCGACGTC-TTATGCGA          100.0
AW16-16S-B39FG-AAGAGGCA-CGTCTAAT          100.0
AX09-16S-B3RMH-GCGTAGTA-TTCTAGCT          100.0
AP14-16S-AT62V-TCGACGTC-TTCTAGCT          100.0
AM15-16S-AT526-CGAGGCTG-AAGGCTAT          100.0
AD22-16S-ATC1H-GCGTAGTA-TCTCTCCG          100.0
AL01-16S-AT526-ATCTCAGG-TTCTAGCT          100.0
AH18-16S-ARRDR-GGAGCTAC-TATCCTCT          100.0
AM13-16S-AT526-TAGGCATG-AAGGCTAT          100.0
AK01-16S-ARRDR-GCGTAGTA-TCTCTCCG          100.0
AC25-16S-ATC1H-ACTCGCTA-CTAAGCCT          100.0
AE02-16S-AR34D-GCGTAGTA-TCGACTAG          100.0
AA31-16S-ATC1H-ATGCGCAG-CTCTCTAT          100.0
AX05-16S-B3RMH-TGCAGCTA-TCGACTAG          100.0
AS01-16S-B39FG-AGGCAGAA-CTCTCTAT          100.0
AB29-16S-ATC1H-TGCAGCTA-GTAAGGAG          100.0
AW19-16S-B39FG-ATCTCAGG-CGTCTAAT          100.0
AI28-16S-ARRDR-ATGCGCAG-AAGGAGTA          100.0
AG22-16S-AR34D-CGATCAGT-GAGCCTTA          100.0
AQ27-16S-AT62V-ATGCGCAG-AAGGCTAT          100.0
AB16-16S-ATC1H-CGATCAGT-TATCCTCT          100.0
AW03-16S-B39FG-CGAGGCTG-CTAAGCCT          100.0
AK24-16S-AT526-GGACTCCT-TTCTAGCT          100.0
AU16-16S-B39FG-TAAGGCGA-CTAAGCCT          100.0
AD15-16S-ATC1H-ACTGAGCG-CGTCTAAT          100.0
AI19-16S-ARRDR-CCTAAGAC-ACTGCATA          100.0
AD26-16S-ATC1H-TAGCGCTC-TCTCTCCG          100.0
AE22-16S-AR34D-CGATCAGT-TTCTAGCT          100.0
AU20-16S-B39FG-GGACTCCT-CTAAGCCT          100.0
AW10-16S-B39FG-AGGCAGAA-CGTCTAAT          100.0
AC22-16S-ATC1H-CGATCAGT-AAGGAGTA          100.0
AP25-16S-AT62V-CGATCAGT-CCTAGAGT          100.0
AA19-16S-ARW7H-AGGCAGAA-TTATGCGA          100.0
AE21-16S-AR34D-CCTAAGAC-TTCTAGCT          100.0
AB11-16S-ATC1H-TACGCTGC-TATCCTCT          100.0
AM17-16S-AT526-GTAGAGGA-AAGGCTAT          100.0
AP21-16S-AT62V-ATGCGCAG-CCTAGAGT          100.0
AC18-16S-ATC1H-ATGCGCAG-AAGGAGTA          100.0
AC10-16S-ATC1H-CGATCAGT-ACTGCATA          100.0
AL03-16S-AT526-CGTACTAG-CCTAGAGT          100.0
AW07-16S-B39FG-ATCTCAGG-CTAAGCCT          100.0
AN08-16S-AT526-CTCTCTAC-TTATGCGA          100.0
AF08-16S-AR34D-GGAGCTAC-GCGTAAGA          100.0
AT06-16S-B39FG-TAAGGCGA-ACTGCATA          100.0
AJ15-16S-ARRDR-TGCAGCTA-CTAAGCCT          100.0
AM26-16S-AT526-CTCTCTAC-GAGCCTTA          100.0
AS12-16S-B39FG-CGTACTAG-TATCCTCT          100.0
AG30-16S-AR34D-ATGCGCAG-TTATGCGA          100.0
AC01-16S-ATC1H-ACTCGCTA-ACTGCATA          100.0
AJ03-16S-ARRDR-TGCAGCTA-AAGGAGTA          100.0
AS26-16S-B39FG-AGGCAGAA-GTAAGGAG          100.0
AG12-16S-AR34D-TCGACGTC-AAGGCTAT          100.0
AB25-16S-ATC1H-TAGCGCTC-GTAAGGAG          100.0
AB14-16S-ATC1H-ACTGAGCG-TATCCTCT          100.0
AU03-16S-B39FG-ATCTCAGG-ACTGCATA          100.0
AS28-16S-B39FG-GGACTCCT-GTAAGGAG          100.0
AM10-16S-AT526-AGGCAGAA-AAGGCTAT          100.0
AK10-16S-AT526-AGGCAGAA-TCGACTAG          100.0
AX08-16S-B3RMH-GGAGCTAC-TTCTAGCT          100.0
AK23-16S-AT526-TCCTGAGC-TTCTAGCT          100.0
AT13-16S-B39FG-CGAGGCTG-ACTGCATA          100.0
AK25-16S-AT526-TAGGCATG-TTCTAGCT          100.0
AQ29-16S-AT62V-ACTGAGCG-AAGGCTAT          100.0
AT04-16S-B39FG-GCTCATGA-GTAAGGAG          100.0
AL25-16S-AT526-ATCTCAGG-GCGTAAGA          100.0
AL02-16S-AT526-TAAGGCGA-CCTAGAGT          100.0
AG18-16S-AR34D-ATGCGCAG-GAGCCTTA          100.0
AG29-16S-AR34D-TACGCTGC-TTATGCGA          100.0
AH29-16S-ARRDR-ACTCGCTA-GTAAGGAG          100.0
AP23-16S-AT62V-ACTGAGCG-CCTAGAGT          100.0
AD21-16S-ATC1H-GGAGCTAC-TCTCTCCG          100.0
AC29-16S-ATC1H-TACGCTGC-CTAAGCCT          100.0
AN16-16S-AT62V-ATGCGCAG-TCGACTAG          100.0
AQ30-16S-AT62V-CCTAAGAC-AAGGCTAT          100.0
AN13-16S-AT62V-GCGTAGTA-TCGACTAG          100.0
AC14-16S-ATC1H-GGAGCTAC-AAGGAGTA          100.0
AU13-16S-B39FG-GTAGAGGA-AAGGAGTA          100.0
AQ21-16S-AT62V-TCGACGTC-CTATTAAG          100.0
AL10-16S-AT526-AAGAGGCA-CCTAGAGT          100.0
AB03-16S-ATC1H-CCTAAGAC-CTCTCTAT          100.0
AM08-16S-AT526-TAAGGCGA-AAGGCTAT          100.0
AB09-16S-ATC1H-GCGTAGTA-TATCCTCT          100.0
AC08-16S-ATC1H-ACTGAGCG-ACTGCATA          100.0
AK28-16S-AT526-AAGAGGCA-TTCTAGCT          100.0
AK17-16S-AT526-GTAGAGGA-TCGACTAG          100.0
AX02-16S-B3RMH-ACTGAGCG-TCGACTAG          100.0
AP07-16S-AT62V-TACGCTGC-TTCTAGCT          100.0
AH21-16S-ARRDR-TACGCTGC-TATCCTCT          100.0
AT05-16S-B39FG-ATCTCAGG-GTAAGGAG          100.0
AP05-16S-AT62V-GCGTAGTA-TTCTAGCT          100.0
AP20-16S-AT62V-TACGCTGC-CCTAGAGT          100.0
AQ26-16S-AT62V-TACGCTGC-AAGGCTAT          100.0
AU09-16S-B39FG-TAGGCATG-AAGGAGTA          100.0
AQ19-16S-AT62V-CGATCAGT-CTATTAAG          100.0
AL04-16S-AT526-AGGCAGAA-CCTAGAGT          100.0
AP13-16S-AT62V-TGCAGCTA-TTCTAGCT          100.0
AS19-16S-B39FG-AAGAGGCA-TATCCTCT          100.0
AX06-16S-B3RMH-TCGACGTC-TCGACTAG          100.0
AH19-16S-ARRDR-GCGTAGTA-TATCCTCT          100.0
AK03-16S-ARRDR-TACGCTGC-TCTCTCCG          100.0
AI09-16S-ARRDR-TGCAGCTA-GTAAGGAG          100.0
AE09-16S-AR34D-CGATCAGT-TCGACTAG          100.0
AL28-16S-AT526-AGGCAGAA-CTATTAAG          100.0
AE19-16S-AR34D-TAGCGCTC-TTCTAGCT          100.0
AB05-16S-ATC1H-TGCAGCTA-CTCTCTAT          100.0
AU07-16S-B39FG-TCCTGAGC-AAGGAGTA          100.0
AS18-16S-B39FG-CGAGGCTG-TATCCTCT          100.0
AX07-16S-B3RMH-ACTCGCTA-TTCTAGCT          100.0
AX04-16S-B3RMH-CGATCAGT-TCGACTAG          100.0
AP06-16S-AT62V-CGGAGCCT-TTCTAGCT          100.0
AQ09-16S-AT62V-TCGACGTC-GCGTAAGA          100.0
AS07-16S-B39FG-AAGAGGCA-CTCTCTAT          100.0
AN12-16S-AT526-GCTCATGA-TTATGCGA          100.0
AM06-16S-AT526-GCTCATGA-CTATTAAG          100.0
AU10-16S-B39FG-CTCTCTAC-AAGGAGTA          100.0
AU02-16S-B39FG-GCTCATGA-ACTGCATA          100.0
AI11-16S-ARRDR-ACTCGCTA-ACTGCATA          100.0
AG11-16S-AR34D-TGCAGCTA-AAGGCTAT          100.0
AD30-16S-ATC1H-TGCAGCTA-TCTCTCCG          100.0
AJ24-16S-ARRDR-ACTGAGCG-CGTCTAAT          100.0
AD02-16S-ATC1H-ACTGAGCG-CTAAGCCT          100.0
AW27-16S-B3RMH-GCGTAGTA-TCGACTAG          100.0
AB12-16S-ATC1H-ATGCGCAG-TATCCTCT          100.0
AT08-16S-B39FG-AGGCAGAA-ACTGCATA          100.0
AC03-16S-ATC1H-GCGTAGTA-ACTGCATA          100.0
AC11-16S-ATC1H-TGCAGCTA-ACTGCATA          100.0
AM04-16S-AT526-AAGAGGCA-CTATTAAG          100.0
AA06-16S-ARW7H-CGTACTAG-GAGCCTTA          100.0
AL06-16S-AT526-GGACTCCT-CCTAGAGT          100.0
AP15-16S-AT62V-ACTCGCTA-CCTAGAGT          100.0
AU04-16S-B39FG-TAAGGCGA-AAGGAGTA          100.0
AG10-16S-AR34D-CGATCAGT-AAGGCTAT          100.0
AQ14-16S-AT62V-TACGCTGC-CTATTAAG          100.0
AA07-16S-ARW7H-AGGCAGAA-GAGCCTTA          100.0
AU18-16S-B39FG-AGGCAGAA-CTAAGCCT          100.0
AF30-16S-AR34D-TCGACGTC-CTATTAAG          100.0
AU06-16S-B39FG-AGGCAGAA-AAGGAGTA          100.0
AW04-16S-B39FG-AAGAGGCA-CTAAGCCT          100.0
AM09-16S-AT526-CGTACTAG-AAGGCTAT          100.0
AL15-16S-AT526-CGTACTAG-GCGTAAGA          100.0
AS16-16S-B39FG-TAGGCATG-TATCCTCT          100.0
AI03-16S-ARRDR-TACGCTGC-GTAAGGAG          100.0
AB13-16S-ATC1H-TAGCGCTC-TATCCTCT          100.0
AE20-16S-AR34D-ACTGAGCG-TTCTAGCT          100.0
AW30-16S-B3RMH-ATGCGCAG-TCGACTAG          100.0
AH08-16S-ARRDR-CGGAGCCT-CTCTCTAT          100.0
AB15-16S-ATC1H-CCTAAGAC-TATCCTCT          100.0
AE07-16S-AR34D-ACTGAGCG-TCGACTAG          100.0
AI24-16S-ARRDR-GGAGCTAC-AAGGAGTA          100.0
AT03-16S-B39FG-GTAGAGGA-GTAAGGAG          100.0
AP26-16S-AT62V-TGCAGCTA-CCTAGAGT          100.0
AI20-16S-ARRDR-CGATCAGT-ACTGCATA          100.0
AW05-16S-B39FG-GTAGAGGA-CTAAGCCT          100.0
AD07-16S-ATC1H-TCGACGTC-CTAAGCCT          100.0
AS03-16S-B39FG-GGACTCCT-CTCTCTAT          100.0
AC24-16S-ATC1H-TCGACGTC-AAGGAGTA          100.0
AB08-16S-ATC1H-GGAGCTAC-TATCCTCT          100.0
AW08-16S-B39FG-TAAGGCGA-CGTCTAAT          100.0
AJ10-16S-ARRDR-ATGCGCAG-CTAAGCCT          100.0
AJ11-16S-ARRDR-TAGCGCTC-CTAAGCCT          100.0
AW14-16S-B39FG-CTCTCTAC-CGTCTAAT          100.0
AD05-16S-ATC1H-CGATCAGT-CTAAGCCT          100.0
AC26-16S-ATC1H-GGAGCTAC-CTAAGCCT          100.0
AK29-16S-AT526-GTAGAGGA-TTCTAGCT          100.0
AN15-16S-AT62V-TACGCTGC-TCGACTAG          100.0
AD24-16S-ATC1H-TACGCTGC-TCTCTCCG          100.0
AW23-16S-B39FG-TCCTGAGC-TCTCTCCG          100.0
AC05-16S-ATC1H-TACGCTGC-ACTGCATA          100.0
AX01-16S-B3RMH-TAGCGCTC-TCGACTAG          100.0
AH11-16S-ARRDR-TAGCGCTC-CTCTCTAT          100.0
AD27-16S-ATC1H-ACTGAGCG-TCTCTCCG          100.0
AA21-16S-ARW7H-GGACTCCT-TTATGCGA          100.0
AI04-16S-ARRDR-ATGCGCAG-GTAAGGAG          100.0
AM02-16S-AT526-CTCTCTAC-CTATTAAG          100.0
AJ07-16S-ARRDR-GCGTAGTA-CTAAGCCT          100.0
AT02-16S-B39FG-AAGAGGCA-GTAAGGAG          100.0
AT12-16S-B39FG-CTCTCTAC-ACTGCATA          100.0
AG03-16S-AR34D-GCGTAGTA-AAGGCTAT          100.0
AK19-16S-AT526-ATCTCAGG-TCGACTAG          100.0
AX13-16S-B3RMH-TAGCGCTC-TTCTAGCT          100.0
AB28-16S-ATC1H-CGATCAGT-GTAAGGAG          100.0
AR01-16S-AT62V-TGCAGCTA-AAGGCTAT          100.0
AL26-16S-AT526-TAAGGCGA-CTATTAAG          100.0
AI01-16S-ARRDR-GCGTAGTA-GTAAGGAG          100.0
AU19-16S-B39FG-TCCTGAGC-CTAAGCCT          100.0
AQ10-16S-AT62V-ACTCGCTA-CTATTAAG          100.0
AG09-16S-AR34D-CCTAAGAC-AAGGCTAT          100.0
AI25-16S-ARRDR-GCGTAGTA-AAGGAGTA          100.0
AX03-16S-B3RMH-CCTAAGAC-TCGACTAG          100.0
AE08-16S-AR34D-CCTAAGAC-TCGACTAG          100.0
AJ16-16S-ARRDR-TCGACGTC-CTAAGCCT          100.0
AH22-16S-ARRDR-ATGCGCAG-TATCCTCT          100.0
AM22-16S-AT526-AGGCAGAA-GAGCCTTA          100.0
AF14-16S-AR34D-ACTGAGCG-GCGTAAGA          100.0
AJ19-16S-ARRDR-GCGTAGTA-CGTCTAAT          100.0
AW11-16S-B39FG-TCCTGAGC-CGTCTAAT          100.0
AW21-16S-B39FG-CGTACTAG-TCTCTCCG          100.0
AK27-16S-AT526-CGAGGCTG-TTCTAGCT          100.0
AB17-16S-ATC1H-TGCAGCTA-TATCCTCT          100.0
AQ31-16S-AT62V-CGATCAGT-AAGGCTAT          100.0
AE14-16S-AR34D-GGAGCTAC-TTCTAGCT          100.0
AC15-16S-ATC1H-GCGTAGTA-AAGGAGTA          100.0
AQ07-16S-AT62V-CGATCAGT-GCGTAAGA          100.0
AP09-16S-AT62V-TAGCGCTC-TTCTAGCT          100.0
AB24-16S-ATC1H-ATGCGCAG-GTAAGGAG          100.0
AP11-16S-AT62V-CCTAAGAC-TTCTAGCT          100.0
AD29-16S-ATC1H-CGATCAGT-TCTCTCCG          100.0
AA26-16S-ARW7H-GTAGAGGA-TTATGCGA          100.0
AW09-16S-B39FG-CGTACTAG-CGTCTAAT          100.0
AC09-16S-ATC1H-CCTAAGAC-ACTGCATA          100.0
AI08-16S-ARRDR-CGATCAGT-GTAAGGAG          100.0
AL12-16S-AT526-GCTCATGA-CCTAGAGT          100.0
AA23-16S-ARW7H-CTCTCTAC-TTATGCGA          100.0
AL20-16S-AT526-CTCTCTAC-GCGTAAGA          100.0
AP08-16S-AT62V-ATGCGCAG-TTCTAGCT          100.0
AK18-16S-AT526-GCTCATGA-TCGACTAG          100.0
AA27-16S-ARW7H-GCTCATGA-TTATGCGA          100.0
AH23-16S-ARRDR-TAGCGCTC-TATCCTCT          100.0
AW24-16S-B39FG-GGACTCCT-TCTCTCCG          100.0
AD08-16S-ATC1H-ACTCGCTA-CGTCTAAT          100.0
AQ06-16S-AT62V-CCTAAGAC-GCGTAAGA          100.0
AK26-16S-AT526-CTCTCTAC-TTCTAGCT          100.0
AN11-16S-AT526-GTAGAGGA-TTATGCGA          100.0
AK20-16S-AT526-TAAGGCGA-TTCTAGCT          100.0
AH24-16S-ARRDR-ACTGAGCG-TATCCTCT          100.0
AF13-16S-AR34D-TAGCGCTC-GCGTAAGA          100.0
AM20-16S-AT526-TAAGGCGA-GAGCCTTA          100.0
AG19-16S-AR34D-TAGCGCTC-GAGCCTTA          100.0
AX17-16S-B3RMH-TGCAGCTA-TTCTAGCT          100.0
AM28-16S-AT526-AAGAGGCA-GAGCCTTA          100.0
AM27-16S-AT526-CGAGGCTG-GAGCCTTA          100.0
AC17-16S-ATC1H-TACGCTGC-AAGGAGTA          100.0
AF28-16S-AR34D-CGATCAGT-CTATTAAG          100.0
AH10-16S-ARRDR-ATGCGCAG-CTCTCTAT          100.0
AK07-16S-ARRDR-CCTAAGAC-TCTCTCCG          100.0
AX14-16S-B3RMH-ACTGAGCG-TTCTAGCT          100.0
AQ05-16S-AT62V-ACTGAGCG-GCGTAAGA          100.0
AT14-16S-B39FG-AAGAGGCA-ACTGCATA          100.0
AP10-16S-AT62V-ACTGAGCG-TTCTAGCT          100.0
AP03-16S-AT62V-ACTCGCTA-TTCTAGCT          100.0
AK02-16S-ARRDR-CGGAGCCT-TCTCTCCG          100.0
AT01-16S-B39FG-CGAGGCTG-GTAAGGAG          100.0
AA20-16S-ARW7H-TCCTGAGC-TTATGCGA          100.0
AL23-16S-AT526-GTAGAGGA-GCGTAAGA          100.0
AK05-16S-ARRDR-TAGCGCTC-TCTCTCCG          100.0
AW18-16S-B39FG-GCTCATGA-CGTCTAAT          100.0
5863-1976-ADT0A-TCGACGTC-CTAAGCCT-L001    100.0
```

In [55]:

```
#combined stats by diagnosis
display(combined_mapping_df['diagnosis'].value_counts().to_frame())
```

|  | diagnosis |
| --- | --- |
| CRSwNP | 207 |
| Control | 168 |
| CRSsNP | 132 |

In [56]:

```
#Compute JSD
combined_counts_df = combined_exp.data_df
%R -i combined_counts_df
%R library(philentropy)
%R combined_jsd_dist <- as.matrix(philentropy::distance(t(combined_counts_df/100), "jensen-shannon"))
%R -o combined_jsd_dist

combined_jsd_dist = pd.DataFrame(combined_jsd_dist, index=combined_counts_df.columns, columns=combined_counts_df.columns)


#Perform PCoA
combined_beta_div_exp = combined_exp.with_data_df(combined_jsd_dist)
combined_dist_matrix_pcoa = combined_beta_div_exp.apply(PCoA)
combined_three_PCs_df = combined_dist_matrix_pcoa.data_df.T[['PC1', 'PC2', 'PC3']]
combined_three_PCs_df_with_metadata = combined_three_PCs_df.join(combined_exp.mapping_df[['dataset']])

#subset the combined_three_PCs_df_with_metadata DataFrame
#one dataframe is for dataset 2 ; and the other is for dataset main
dataset2_combined_three_PCs_df_with_metadata = combined_three_PCs_df_with_metadata[combined_three_PCs_df_with_metadata['dataset']=='dataset_two']
datasetmain_combined_three_PCs_df_with_metadata = combined_three_PCs_df_with_metadata[combined_three_PCs_df_with_metadata['dataset']=='dataset_main']
```

```
/home/ahmed/dev/biomenv3/lib/python3.6/site-packages/rpy2/rinterface/__init__.py:185: RRuntimeWarning: Metric: 'jensen-shannon' using unit: 'log'.

  warnings.warn(x, RRuntimeWarning)
/home/ahmed/dev/biomenv3/lib/python3.6/site-packages/skbio/stats/ordination/_principal_coordinate_analysis.py:111: RuntimeWarning: The result contains negative eigenvalues. Please compare their magnitude with the magnitude of some of the largest positive eigenvalues. If the negative ones are smaller, it's probably safe to ignore them, but if they are large in magnitude, the results won't be useful. See the Notes section for more details. The smallest eigenvalue is -3.8674685089255334 and the largest is 24.60851456897876.
  RuntimeWarning
```

#### Now we perform the "semi-supervized K-Means"¶

.. using the K-Means model originally performed on the main dataset - we use the scikit\_learn model's .predict() function.

Alternatively, we could have used a new Random Forest classifier, for example.

In [57]:

```
kmeans_predicted = kmeans_main.predict(dataset2_combined_three_PCs_df_with_metadata[['PC1', 'PC2']])
dataset2_semisupervised_clusters = pd.Series(["cluster_" + str(x + 1) for x in kmeans_predicted], index=dataset2_three_PCs_df.index)

#name the clusters
dataset2_microbiotypes = dataset2_semisupervised_clusters.replace(microbiotype_rename_dict)

dataset2_three_PCs_df_with_metadata['semisupervised_microbiotype'] = dataset2_microbiotypes
exp_genus_dataset2.mapping_df['semisupervised_microbiotype'] = dataset2_microbiotypes
```

And that is the composition of the microbiotypes using this semi-supervised approach.

In [58]:

```
exp_genus_dataset2\
.to_relative_abundance()\
.apply(TopAbundantObservations(10))\
.groupby("semisupervised_microbiotype").data_df
```

Out[58]:

| semisupervised\_microbiotype | microbiotype\_1 | microbiotype\_2 | microbiotype\_3 |
| --- | --- | --- | --- |
| Other | 11.262821 | 7.250000 | 15.427419 |
| g\_\_Alloiococcus | 7.371795 | 0.092593 | 0.000000 |
| g\_\_Corynebacterium | 61.685897 | 9.842593 | 3.161290 |
| g\_\_Fusobacterium | 0.326923 | 2.648148 | 6.129032 |
| g\_\_Haemophilus | 0.115385 | 3.037037 | 28.854839 |
| g\_\_Moraxella | 1.621795 | 0.898148 | 12.733871 |
| g\_\_Pseudomonas | 0.243590 | 0.444444 | 6.379032 |
| g\_\_Staphylococcus | 11.673077 | 75.444444 | 3.830645 |
| g\_\_Streptococcus | 1.070513 | 0.240741 | 12.000000 |
| g\_\_unidentified (f\_\_Enterobacteriaceae) | 2.378205 | 0.055556 | 8.024194 |
| g\_\_unidentified (f\_\_Neisseriaceae) | 2.250000 | 0.046296 | 3.459677 |

.. and compare it to the taxonomy obtained from the unsupervised approach.

In [59]:

```
exp_genus_dataset2_top10.groupby("microbiotype").data_df
```

Out[59]:

| microbiotype | microbiotype\_1 | microbiotype\_2 | microbiotype\_3 |
| --- | --- | --- | --- |
| Other | 11.559211 | 7.528846 | 14.492424 |
| g\_\_Alloiococcus | 7.565789 | 0.000000 | 0.075758 |
| g\_\_Corynebacterium | 62.144737 | 11.894231 | 2.992424 |
| g\_\_Fusobacterium | 0.335526 | 1.932692 | 6.401515 |
| g\_\_Haemophilus | 0.118421 | 0.028846 | 29.568182 |
| g\_\_Moraxella | 1.664474 | 0.000000 | 12.696970 |
| g\_\_Pseudomonas | 0.250000 | 0.461538 | 5.992424 |
| g\_\_Staphylococcus | 10.513158 | 77.875000 | 5.659091 |
| g\_\_Streptococcus | 1.098684 | 0.173077 | 11.333333 |
| g\_\_unidentified (f\_\_Enterobacteriaceae) | 2.440789 | 0.057692 | 7.537879 |
| g\_\_unidentified (f\_\_Neisseriaceae) | 2.309211 | 0.048077 | 3.250000 |

In [60]:

```
#combine dataset two's semisupervised microbiotype assignments with the original main dataset
#microbiotype assignment into one series for the purposes of the combined figure / scatter plot
combined_microbiotypes_series = pd.concat([exp_genus.mapping_df['microbiotype'],
                                          exp_genus_dataset2.mapping_df['semisupervised_microbiotype']])

#insert this series back into mapping file of the combined Experiment object
#and into the combined_three_PCs_df_with_metadata dataframe
combined_exp.mapping_df['microbiotype'] = combined_microbiotypes_series
combined_three_PCs_df_with_metadata['microbiotype'] = combined_microbiotypes_series

display(combined_microbiotypes_series)
```

```
AF18-16S-AR34D-TCGACGTC-GCGTAAGA          microbiotype_1
AX02-16S-B3RMH-ACTGAGCG-TCGACTAG          microbiotype_2
AK17-16S-AT526-GTAGAGGA-TCGACTAG          microbiotype_2
AK28-16S-AT526-AAGAGGCA-TTCTAGCT          microbiotype_1
AC08-16S-ATC1H-ACTGAGCG-ACTGCATA          microbiotype_2
AB09-16S-ATC1H-GCGTAGTA-TATCCTCT          microbiotype_2
AM08-16S-AT526-TAAGGCGA-AAGGCTAT          microbiotype_1
AB03-16S-ATC1H-CCTAAGAC-CTCTCTAT          microbiotype_1
AL10-16S-AT526-AAGAGGCA-CCTAGAGT          microbiotype_2
AQ21-16S-AT62V-TCGACGTC-CTATTAAG          microbiotype_2
AU13-16S-B39FG-GTAGAGGA-AAGGAGTA          microbiotype_1
AC14-16S-ATC1H-GGAGCTAC-AAGGAGTA          microbiotype_1
AN13-16S-AT62V-GCGTAGTA-TCGACTAG          microbiotype_1
AQ30-16S-AT62V-CCTAAGAC-AAGGCTAT          microbiotype_3
AP07-16S-AT62V-TACGCTGC-TTCTAGCT          microbiotype_3
AN16-16S-AT62V-ATGCGCAG-TCGACTAG          microbiotype_1
AD21-16S-ATC1H-GGAGCTAC-TCTCTCCG          microbiotype_2
AP23-16S-AT62V-ACTGAGCG-CCTAGAGT          microbiotype_2
AH29-16S-ARRDR-ACTCGCTA-GTAAGGAG          microbiotype_1
AG29-16S-AR34D-TACGCTGC-TTATGCGA          microbiotype_3
AG18-16S-AR34D-ATGCGCAG-GAGCCTTA          microbiotype_1
AL02-16S-AT526-TAAGGCGA-CCTAGAGT          microbiotype_2
AL25-16S-AT526-ATCTCAGG-GCGTAAGA          microbiotype_3
AT04-16S-B39FG-GCTCATGA-GTAAGGAG          microbiotype_2
AQ29-16S-AT62V-ACTGAGCG-AAGGCTAT          microbiotype_2
AK25-16S-AT526-TAGGCATG-TTCTAGCT          microbiotype_1
AT13-16S-B39FG-CGAGGCTG-ACTGCATA          microbiotype_2
AK23-16S-AT526-TCCTGAGC-TTCTAGCT          microbiotype_1
AX08-16S-B3RMH-GGAGCTAC-TTCTAGCT          microbiotype_1
AC29-16S-ATC1H-TACGCTGC-CTAAGCCT          microbiotype_1
                                               ...      
5765-1597-ACAN3-CTCTCTAC-TCTCTCCG-L001    microbiotype_3
5717-1448-ACAN3-TAGGCATG-CGTCTAAT-L001    microbiotype_3
1631-1265-ACAN3-AGGCAGAA-ACTGCATA-L001    microbiotype_3
3853-1824-ACAN3-AGGCAGAA-CGTCTAAT-L001    microbiotype_3
5894-2075-ADT0A-CCTAAGAC-AAGGAGTA-L001    microbiotype_3
5551-955-ACAN3-TAAGGCGA-CTCTCTAT-L001     microbiotype_2
5630-1302-ACAN3-TAGGCATG-ACTGCATA-L001    microbiotype_2
5600-1193-ACAN3-GTAGAGGA-GTAAGGAG-L001    microbiotype_1
5640-1346-ACAN3-GCTCATGA-ACTGCATA-L001    microbiotype_3
5634-1320-ACAN3-CGAGGCTG-ACTGCATA-L001    microbiotype_1
5558-994-ACAN3-GGACTCCT-CTCTCTAT-L001     microbiotype_3
5524-867-ACAN3-ATCTCAGG-AAGGAGTA-L001     microbiotype_1
5876-2007-ADT0A-CGATCAGT-CGTCTAAT-L001    microbiotype_1
5728-1477-ACAN3-TAGGCATG-CTAAGCCT-L001    microbiotype_3
5595-1168-ACAN3-CGAGGCTG-GTAAGGAG-L001    microbiotype_3
1415-1855-ADT0A-ACTCGCTA-CGTCTAAT-L001    microbiotype_2
5802-1745-ACAN3-GTAGAGGA-CTAAGCCT-L001    microbiotype_1
5897-2084-ADT0A-TGCAGCTA-CGTCTAAT-L001    microbiotype_2
5807-1772-ACAN3-GTAGAGGA-TCTCTCCG-L001    microbiotype_2
5577-1064-ACAN3-GTAGAGGA-TATCCTCT-L001    microbiotype_3
5888-2051-ADT0A-GCGTAGTA-CGTCTAAT-L001    microbiotype_2
5866-1983-ADT0A-ACTCGCTA-CTAAGCCT-L001    microbiotype_3
5721-1452-ACAN3-AGGCAGAA-TCTCTCCG-L001    microbiotype_1
5589-1115-ACAN3-AGGCAGAA-GTAAGGAG-L001    microbiotype_2
5562-986-ACAN3-AAGAGGCA-CTCTCTAT-L001     microbiotype_2
5835-1908-ADT0A-TAGCGCTC-TCTCTCCG-L001    microbiotype_3
5900-2086-ADT0A-TGCAGCTA-CTAAGCCT-L001    microbiotype_1
5780-1645-ACAN3-AAGAGGCA-CTAAGCCT-L001    microbiotype_1
5568-1002-ACAN3-GTAGAGGA-CTCTCTAT-L001    microbiotype_1
5863-1976-ADT0A-TCGACGTC-CTAAGCCT-L001    microbiotype_2
Length: 507, dtype: object
```

In [61]:

```
comparison_unsupervised_vs_semisupervised_df = \
pd.concat([exp_genus_dataset2_top10.groupby("microbiotype").data_df,
          combined_exp[combined_exp.Sample.c.dataset=='dataset_two']\
.apply(TopAbundantObservations(10))\
.groupby("microbiotype").data_df], keys=['unsupervised approach', 'semi-supervised approach'], axis=0)\
.unstack(level=0)

display(comparison_unsupervised_vs_semisupervised_df)

comparison_unsupervised_vs_semisupervised_df\
.to_csv("./results/microbiotyping/tables/SUPP_unsupervised_vs_semisupervised.tsv",
        sep="\t")
```

| microbiotype | microbiotype\_1 | | microbiotype\_2 | | microbiotype\_3 | |
| --- | --- | --- | --- | --- | --- | --- |
|  | unsupervised approach | semi-supervised approach | unsupervised approach | semi-supervised approach | unsupervised approach | semi-supervised approach |
| Other | 11.559211 | 11.262821 | 7.528846 | 7.250000 | 14.492424 | 15.427419 |
| g\_\_Alloiococcus | 7.565789 | 7.371795 | 0.000000 | 0.092593 | 0.075758 | 0.000000 |
| g\_\_Corynebacterium | 62.144737 | 61.685897 | 11.894231 | 9.842593 | 2.992424 | 3.161290 |
| g\_\_Fusobacterium | 0.335526 | 0.326923 | 1.932692 | 2.648148 | 6.401515 | 6.129032 |
| g\_\_Haemophilus | 0.118421 | 0.115385 | 0.028846 | 3.037037 | 29.568182 | 28.854839 |
| g\_\_Moraxella | 1.664474 | 1.621795 | 0.000000 | 0.898148 | 12.696970 | 12.733871 |
| g\_\_Pseudomonas | 0.250000 | 0.243590 | 0.461538 | 0.444444 | 5.992424 | 6.379032 |
| g\_\_Staphylococcus | 10.513158 | 11.673077 | 77.875000 | 75.444444 | 5.659091 | 3.830645 |
| g\_\_Streptococcus | 1.098684 | 1.070513 | 0.173077 | 0.240741 | 11.333333 | 12.000000 |
| g\_\_unidentified (f\_\_Enterobacteriaceae) | 2.440789 | 2.378205 | 0.057692 | 0.055556 | 7.537879 | 8.024194 |
| g\_\_unidentified (f\_\_Neisseriaceae) | 2.309211 | 2.250000 | 0.048077 | 0.046296 | 3.250000 | 3.459677 |

### Explore the differently-assigned samples between the unsupervised and semi-supervised methods¶

Here we note the difference in assignment between the unsupervised and semi-supervised approaches.

In [62]:

```
exp_genus_dataset2.mapping_df[['microbiotype','semisupervised_microbiotype']]
```

Out[62]:

|  | microbiotype | semisupervised\_microbiotype |
| --- | --- | --- |
| 5645-1360-ACAN3-TAAGGCGA-AAGGAGTA-L001 | microbiotype\_1 | microbiotype\_1 |
| 5650-1392-ACAN3-TAGGCATG-AAGGAGTA-L001 | microbiotype\_1 | microbiotype\_1 |
| 5885-2062-ADT0A-CCTAAGAC-TCTCTCCG-L001 | microbiotype\_2 | microbiotype\_2 |
| 5596-1175-ACAN3-AAGAGGCA-GTAAGGAG-L001 | microbiotype\_1 | microbiotype\_1 |
| 5880-2017-ADT0A-CGATCAGT-CTAAGCCT-L001 | microbiotype\_1 | microbiotype\_1 |
| 5857-1961-ACAN3-CGTACTAG-CGTCTAAT-L001 | microbiotype\_3 | microbiotype\_3 |
| 5769-1604-ACAN3-AAGAGGCA-CGTCTAAT-L001 | microbiotype\_3 | microbiotype\_3 |
| 5718-1449-ACAN3-TAAGGCGA-TCTCTCCG-L001 | microbiotype\_2 | microbiotype\_2 |
| 5567-1007-ACAN3-GCTCATGA-CTCTCTAT-L001 | microbiotype\_1 | microbiotype\_1 |
| 5571-1023-ACAN3-TAAGGCGA-TATCCTCT-L001 | microbiotype\_1 | microbiotype\_1 |
| 5572-1025-ACAN3-CGTACTAG-TATCCTCT-L001 | microbiotype\_2 | microbiotype\_2 |
| 5051-2113-ADT0A-ACTGAGCG-CGTCTAAT-L001 | microbiotype\_1 | microbiotype\_1 |
| 5861-1970-ADT0A-TACGCTGC-CGTCTAAT-L001 | microbiotype\_1 | microbiotype\_1 |
| 5830-1889-ADT0A-ACTCGCTA-TCTCTCCG-L001 | microbiotype\_3 | microbiotype\_3 |
| 5628-1295-ACAN3-TCCTGAGC-ACTGCATA-L001 | microbiotype\_1 | microbiotype\_1 |
| 5552-1374-ACAN3-TCCTGAGC-CTCTCTAT-L001 | microbiotype\_3 | microbiotype\_3 |
| 5862-1973-ADT0A-ATGCGCAG-CTAAGCCT-L001 | microbiotype\_1 | microbiotype\_1 |
| 5812-1797-ADT0A-ATGCGCAG-CGTCTAAT-L001 | microbiotype\_3 | microbiotype\_3 |
| 5829-1888-ADT0A-ACTGAGCG-CTAAGCCT-L001 | microbiotype\_3 | microbiotype\_3 |
| 5594-1151-ACAN3-CTCTCTAC-GTAAGGAG-L001 | microbiotype\_2 | microbiotype\_2 |
| 2911-2010-ACAN3-CTCTCTAC-CGTCTAAT-L001 | microbiotype\_1 | microbiotype\_1 |
| 5559-992-ACAN3-TAGGCATG-CTCTCTAT-L001 | microbiotype\_3 | microbiotype\_3 |
| 5819-1830-ADT0A-CGGAGCCT-CTAAGCCT-L001 | microbiotype\_1 | microbiotype\_1 |
| 5824-1863-ADT0A-GGAGCTAC-TCTCTCCG-L001 | microbiotype\_3 | microbiotype\_3 |
| 5488-695-ACAN3-GTAGAGGA-AAGGAGTA-L001 | microbiotype\_2 | microbiotype\_2 |
| 5637-1326-ACAN3-AAGAGGCA-ACTGCATA-L001 | microbiotype\_2 | microbiotype\_2 |
| 5860-1971-ADT0A-CCTAAGAC-CTAAGCCT-L001 | microbiotype\_1 | microbiotype\_1 |
| 5503-772-ACAN3-GCTCATGA-AAGGAGTA-L001 | microbiotype\_3 | microbiotype\_3 |
| 5485-688-ACAN3-AAGAGGCA-AAGGAGTA-L001 | microbiotype\_3 | microbiotype\_2 |
| 5536-902-ACAN3-AGGCAGAA-CTAAGCCT-L001 | microbiotype\_1 | microbiotype\_1 |
| ... | ... | ... |
| 5765-1597-ACAN3-CTCTCTAC-TCTCTCCG-L001 | microbiotype\_3 | microbiotype\_3 |
| 5717-1448-ACAN3-TAGGCATG-CGTCTAAT-L001 | microbiotype\_3 | microbiotype\_3 |
| 1631-1265-ACAN3-AGGCAGAA-ACTGCATA-L001 | microbiotype\_3 | microbiotype\_3 |
| 3853-1824-ACAN3-AGGCAGAA-CGTCTAAT-L001 | microbiotype\_3 | microbiotype\_3 |
| 5894-2075-ADT0A-CCTAAGAC-AAGGAGTA-L001 | microbiotype\_3 | microbiotype\_3 |
| 5551-955-ACAN3-TAAGGCGA-CTCTCTAT-L001 | microbiotype\_3 | microbiotype\_2 |
| 5630-1302-ACAN3-TAGGCATG-ACTGCATA-L001 | microbiotype\_2 | microbiotype\_2 |
| 5600-1193-ACAN3-GTAGAGGA-GTAAGGAG-L001 | microbiotype\_1 | microbiotype\_1 |
| 5640-1346-ACAN3-GCTCATGA-ACTGCATA-L001 | microbiotype\_3 | microbiotype\_3 |
| 5634-1320-ACAN3-CGAGGCTG-ACTGCATA-L001 | microbiotype\_1 | microbiotype\_1 |
| 5558-994-ACAN3-GGACTCCT-CTCTCTAT-L001 | microbiotype\_3 | microbiotype\_3 |
| 5524-867-ACAN3-ATCTCAGG-AAGGAGTA-L001 | microbiotype\_1 | microbiotype\_1 |
| 5876-2007-ADT0A-CGATCAGT-CGTCTAAT-L001 | microbiotype\_1 | microbiotype\_1 |
| 5728-1477-ACAN3-TAGGCATG-CTAAGCCT-L001 | microbiotype\_3 | microbiotype\_3 |
| 5595-1168-ACAN3-CGAGGCTG-GTAAGGAG-L001 | microbiotype\_3 | microbiotype\_3 |
| 1415-1855-ADT0A-ACTCGCTA-CGTCTAAT-L001 | microbiotype\_2 | microbiotype\_2 |
| 5802-1745-ACAN3-GTAGAGGA-CTAAGCCT-L001 | microbiotype\_1 | microbiotype\_1 |
| 5897-2084-ADT0A-TGCAGCTA-CGTCTAAT-L001 | microbiotype\_2 | microbiotype\_2 |
| 5807-1772-ACAN3-GTAGAGGA-TCTCTCCG-L001 | microbiotype\_2 | microbiotype\_2 |
| 5577-1064-ACAN3-GTAGAGGA-TATCCTCT-L001 | microbiotype\_3 | microbiotype\_3 |
| 5888-2051-ADT0A-GCGTAGTA-CGTCTAAT-L001 | microbiotype\_2 | microbiotype\_2 |
| 5866-1983-ADT0A-ACTCGCTA-CTAAGCCT-L001 | microbiotype\_3 | microbiotype\_3 |
| 5721-1452-ACAN3-AGGCAGAA-TCTCTCCG-L001 | microbiotype\_1 | microbiotype\_1 |
| 5589-1115-ACAN3-AGGCAGAA-GTAAGGAG-L001 | microbiotype\_2 | microbiotype\_2 |
| 5562-986-ACAN3-AAGAGGCA-CTCTCTAT-L001 | microbiotype\_2 | microbiotype\_2 |
| 5835-1908-ADT0A-TAGCGCTC-TCTCTCCG-L001 | microbiotype\_3 | microbiotype\_3 |
| 5900-2086-ADT0A-TGCAGCTA-CTAAGCCT-L001 | microbiotype\_1 | microbiotype\_1 |
| 5780-1645-ACAN3-AAGAGGCA-CTAAGCCT-L001 | microbiotype\_2 | microbiotype\_1 |
| 5568-1002-ACAN3-GTAGAGGA-CTCTCTAT-L001 | microbiotype\_1 | microbiotype\_1 |
| 5863-1976-ADT0A-TCGACGTC-CTAAGCCT-L001 | microbiotype\_2 | microbiotype\_2 |

97 rows × 2 columns

In [63]:

```
comparison_classification = exp_genus_dataset2.mapping_df['microbiotype'] == exp_genus_dataset2.mapping_df['semisupervised_microbiotype']

display(comparison_classification[comparison_classification == False])

differently_classified_samples = comparison_classification[comparison_classification == False].index

results['n_differently_classified_samples'] = len(differently_classified_samples)
results['percent_differently_classified_samples'] = round(results['n_differently_classified_samples'] / results['n_dataset2'] * 100, 2)

print("It appears that there were only *{n_diff_samples}* samples classified differently between both approaches."\
      .format(n_diff_samples=results['n_differently_classified_samples']))
```

```
5485-688-ACAN3-AAGAGGCA-AAGGAGTA-L001     False
5551-955-ACAN3-TAAGGCGA-CTCTCTAT-L001     False
5780-1645-ACAN3-AAGAGGCA-CTAAGCCT-L001    False
dtype: bool
```

```
It appears that there were only *3* samples classified differently between both approaches.
```

In [64]:

```
from omicexperiment.transforms.sample import KeepSamples
display(exp_genus_dataset2_top10.apply(KeepSamples(differently_classified_samples)).data_df)
exp_genus_dataset2_top10.apply(KeepSamples(differently_classified_samples)).plot(backend="matplotlib")
print(differently_classified_samples)
```

|  | 5485-688-ACAN3-AAGAGGCA-AAGGAGTA-L001 | 5551-955-ACAN3-TAAGGCGA-CTCTCTAT-L001 | 5780-1645-ACAN3-AAGAGGCA-CTAAGCCT-L001 |
| --- | --- | --- | --- |
| cluster |  |  |  |
| Other | 0.00 | 0.00 | 0.00 |
| g\_\_Alloiococcus | 2.50 | 0.00 | 0.00 |
| g\_\_Corynebacterium | 0.75 | 0.00 | 44.25 |
| g\_\_Fusobacterium | 0.00 | 21.25 | 0.00 |
| g\_\_Haemophilus | 31.75 | 49.50 | 0.00 |
| g\_\_Moraxella | 24.25 | 0.00 | 0.00 |
| g\_\_Pseudomonas | 0.00 | 0.00 | 0.00 |
| g\_\_Staphylococcus | 40.75 | 27.25 | 55.75 |
| g\_\_Streptococcus | 0.00 | 2.00 | 0.00 |
| g\_\_unidentified (f\_\_Enterobacteriaceae) | 0.00 | 0.00 | 0.00 |
| g\_\_unidentified (f\_\_Neisseriaceae) | 0.00 | 0.00 | 0.00 |

```
Index(['5485-688-ACAN3-AAGAGGCA-AAGGAGTA-L001',
       '5551-955-ACAN3-TAAGGCGA-CTCTCTAT-L001',
       '5780-1645-ACAN3-AAGAGGCA-CTAAGCCT-L001'],
      dtype='object')
```

In [65]:

```
exp_genus_dataset2_top10.mapping_df.T[differently_classified_samples].T['microbiotype']
```

Out[65]:

```
5485-688-ACAN3-AAGAGGCA-AAGGAGTA-L001     microbiotype_3
5551-955-ACAN3-TAAGGCGA-CTCTCTAT-L001     microbiotype_3
5780-1645-ACAN3-AAGAGGCA-CTAAGCCT-L001    microbiotype_2
Name: microbiotype, dtype: object
```

In [66]:

```
exp_genus_dataset2_top10.mapping_df.T[differently_classified_samples].T['semisupervised_microbiotype']
```

Out[66]:

```
5485-688-ACAN3-AAGAGGCA-AAGGAGTA-L001     microbiotype_2
5551-955-ACAN3-TAAGGCGA-CTCTCTAT-L001     microbiotype_2
5780-1645-ACAN3-AAGAGGCA-CTAAGCCT-L001    microbiotype_1
Name: semisupervised_microbiotype, dtype: object
```

We wonder then whether Staph species assignment could help us obtain a more definite classification for these samples.

In [67]:

```
exp_species_staphonly_dataset2 = exp_dataset2.to_relative_abundance()\
                                    .apply(exp_dataset2.Taxonomy.genus=='g__Staphylococcus')\
                                    .apply(TaxonomyGroupBy("species"))\

fig_staph_diff = exp_species_staphonly_dataset2\
.apply(KeepSamples(differently_classified_samples))\
.plot(backend='matplotlib')

ax_staph_diff = fig_staph_diff.axes[0]

ax_staph_diff.set_ylim(0,100)
```

Out[67]:

```
(0, 100)
```

The samples with Staph assigned as *Staph epidermidis* is then more naturally classified as microbiotype 1.

### SUPPLEMENTARY RESULT: Investigating species-level assignment in DataSet 2¶

We performed a post-hoc inspection of species-level assignment in Dataset Two, to investigate whether this finding will be reproducible in a separate dataset.

In [68]:

```
exp_dataset2.to_relative_abundance()\
    .apply(TaxonomyGroupBy("species"))\
    .apply(TopAbundantObservations(10)).to_relative_abundance()\
    .groupby("microbiotype").plot(backend='matplotlib')
```

Out[68]:

Interestingly, this also confirmed clustering of almost all Staphylococcus aureus species in microbiotype 2.

## Final Figure demonstrating both the unsupervised and semi-supervised methods¶

In [69]:

```
fig = pyplot.figure(figsize=(15, 15))
grid = pyplot.GridSpec(2, 2, wspace=0.2, hspace=0.3)
lower_row_of_grid = grid[1,:]
inner_grid = gridspec.GridSpecFromSubplotSpec(1, 3, lower_row_of_grid, wspace=0.1)

#colours of scatter
colours = ['lavender', 'lightgoldenrodyellow', 'pink']
colours_2 = ['royalblue', 'orange', 'crimson']

#colours of bar plot
from matplotlib.colors import to_hex

cmap_tab20 = pyplot.get_cmap('tab20')

colours_barplot = [to_hex(c) for c in list(cmap_tab20.colors[::2])]
colours_barplot_light = [to_hex(c) for c in list(cmap_tab20.colors[1::2])]
colours_barplot_11 = colours + [to_hex(cmap_tab20.colors[::2][0])]

#add an additional colour for Fusobacterium
#taxa_colours['g__Fusobacterium'] = colours_barplot_light[0]
#colours_barplot_12 = pd.Series(taxa_colours).reindex(exp_genus_other.data_df.index)

#Setup the dataframes#####
#here we subset the combined_three_PCs_df_with_metadata DataFrame
#one dataframe is for dataset 2 ; and the other is for dataset main
dataset2_combined_three_PCs_df_with_metadata = combined_three_PCs_df_with_metadata[combined_three_PCs_df_with_metadata['dataset']=='dataset_two']
datasetmain_combined_three_PCs_df_with_metadata = combined_three_PCs_df_with_metadata[combined_three_PCs_df_with_metadata['dataset']=='dataset_main']

#definition of dataframes:
#dataset2_three_PCs_df_with_metadata: principal coordinates of dataset2 unsupervised approach
#dataset2_combined_three_PCs_df_with_metadata: PCs of "dataset2" semi-supervised approach
#datasetmain_combined_three_PCs_df_with_metadata: PCs of "main dataset" with the original unsupervised microbiotypes 

ax1 = fig.add_subplot(grid[0,0])
scatters = []
for i, cluster in enumerate(['microbiotype_1', 'microbiotype_2', 'microbiotype_3']):
    scatter = ax1.scatter(dataset2_three_PCs_df_with_metadata[dataset2_three_PCs_df_with_metadata['microbiotype']==cluster]['PC1'],
                   dataset2_three_PCs_df_with_metadata[dataset2_three_PCs_df_with_metadata['microbiotype']==cluster]['PC2'],
                   c=colours_2[i])
    scatters.append(scatter)

ax1.set_title('Independent K-Means clustering of Dataset Two samples \n using our described K-means microbiotyping approach \n "Unsupervised"')
ax1.set_xlabel("PC1")
ax1.set_ylabel("PC2")
ax1.legend(scatters, ['cluster_1', 'cluster_2', 'cluster_3'])

ax2 = fig.add_subplot(grid[0,1])
for i, cluster in enumerate(['microbiotype_1', 'microbiotype_2', 'microbiotype_3']):
    ax2.scatter(datasetmain_combined_three_PCs_df_with_metadata[datasetmain_combined_three_PCs_df_with_metadata['microbiotype']==cluster]['PC1'],
                   datasetmain_combined_three_PCs_df_with_metadata[datasetmain_combined_three_PCs_df_with_metadata['microbiotype']==cluster]['PC2'],
                   c=colours[i])

scatters=[]
for i, cluster in enumerate(['microbiotype_1', 'microbiotype_2', 'microbiotype_3']):
    scatter = ax2.scatter(dataset2_combined_three_PCs_df_with_metadata[dataset2_combined_three_PCs_df_with_metadata['microbiotype']==cluster]['PC1'],
                dataset2_combined_three_PCs_df_with_metadata[dataset2_combined_three_PCs_df_with_metadata['microbiotype']==cluster]['PC2'],
                c=colours_2[i])
    scatters.append(scatter)

ax2.set_title('Prediction of microbiotypes on Dataset Two samples \n using the K-means model fitted on the Main Dataset \n "Semi-supervised"')
ax2.set_xlabel("PC1")
ax2.set_ylabel("PC2")
ax2.legend(scatters, ['microbiotype_1', 'microbiotype_2', 'microbiotype_3'])


#prepare dataframes
from omicexperiment.transforms.observation import TopAbundantObservations
combined_exp_top = combined_exp.apply(TopAbundantObservations(10))
combined_cumsum_df = return_cumsum_df(combined_exp_top.groupby('microbiotype'))

other_taxa = list(exp_genus_dataset2.data_df.index.difference(combined_cumsum_df.index))
exp_genus_dataset2_other = exp_genus_dataset2.to_relative_abundance().apply(BinObservations(other_taxa))
dataset2_cumsum_df = return_cumsum_df(exp_genus_dataset2_other.groupby('microbiotype'))


#microbiotypes of dataset 2 "unsupervised"
######################################
ax3 = fig.add_subplot(inner_grid[0,0])

ax3.set_ylim(0,100)
ax3.set_ylabel("Relative Abundance (%)")
ax3.set_title('Dataset Two\n"Unsupervised approach"')
ax3.set_xticklabels(['cluster_1', 'cluster_2', 'cluster_3'])
bars3 = []

for i, arr in enumerate(dataset2_cumsum_df.as_matrix()):
    bar_taxon = dataset2_cumsum_df.index[i]
    bar_ = ax3.bar(dataset2_cumsum_df.columns, arr, width=0.6, color=taxa_colours[bar_taxon])
    bars3.append(bar_[0])
    
#microbiotypes of the combined dataset
######################################
ax4 = fig.add_subplot(inner_grid[0,2], sharey=ax3)

ax4.set_ylim(0,100)
ax4.set_ylabel("")
ax4.set_title('Combined Main Dataset + Dataset Two\n"Semi-supervised approach"')
bars4 = []

for i, arr in enumerate(combined_cumsum_df.as_matrix()):
    bar_taxon = combined_cumsum_df.index[i]
    bar_ = ax4.bar(combined_cumsum_df.columns, arr, width=0.6, color=taxa_colours[bar_taxon])
    bars4.append(bar_[0])

#legend
##############
ax_leg = fig.add_subplot(inner_grid[0,1])
ax_leg.axis('off')
ax_leg.legend(bars4, list(combined_cumsum_df.index), loc='center', title="Taxonomy composition - genus level\n")

#figtexts
##############
pyplot.figtext(0.10,0.91, "A.", fontsize=16, axes=ax12)
pyplot.figtext(0.51,0.91, "B.", fontsize=16, axes=ax1)
pyplot.figtext(0.10,0.485, "C.", fontsize=16, axes=ax20)
pyplot.figtext(0.63,0.485, "D.", fontsize=16, axes=ax20)

fig.savefig("./results/microbiotyping/figures/predicted_microbiotypes_for_dataset_two.jpg", bbox_inches="tight", dpi=300)
```

## Addressing criticisms to the concept of enterotypes¶

#### Investigating the distribution of the Berger-Parker index¶

In [70]:

```
berger_parker_index_series = combined_exp.to_relative_abundance().data_df.apply(lambda x: x.max())

#density histograme
hist_y, hist_x = np.histogram(berger_parker_index_series,
                              bins=[0,10,20,30,40,50,60,70,80,90,100],
                              density=True)

#cumulative density function histogram df (note use of np.cumsum())
histogram_df = pd.DataFrame([pd.Series((hist_x / 100), name='x'), pd.Series((np.cumsum(hist_y)*10), name='y')]).T
histogram_df = histogram_df.set_index('x', drop=False)

display(histogram_df)


results['percent_samples_berger_parker_50_or_less'] = round(histogram_df.xs(0.5)['y'] * 100, 1)
results['percent_samples_berger_parker_greater_than_50'] = 100 - results['percent_samples_berger_parker_50_or_less']
results['percent_samples_berger_parker_greater_than_70'] = 100 - round(histogram_df.xs(0.7)['y'] * 100, 1)


#Figure
fig_histo = pyplot.figure(figsize=(6.5, 4))
grid_histo = pyplot.GridSpec(1, 1, wspace=0.2, hspace=0.2)

ax_h = fig_histo.add_subplot(grid_histo[0,0])

histo_bar = ax_h.bar(x=histogram_df.x, height=histogram_df.y, width=0.1, align='edge')
ax_h.legend([histo_bar], ["n = {} samples".format(len(combined_exp.samples))])
ax_h.set_xticks(histogram_df.x)
ax_h.axvline(0.5, c='black', linestyle='--')
ax_h.set_xlabel("Berger-Parker Index\n(proportional abundance of the most dominant taxon)")
ax_h.set_ylabel("Cumulative Distribution Function")

fig_histo.savefig("./results/microbiotyping/figures/SUPP_berger_parker.jpg", bbox_inches='tight', dpi=300)
```

|  | x | y |
| --- | --- | --- |
| x |  |  |
| 0.0 | 0.0 | 0.000000 |
| 0.1 | 0.1 | 0.000000 |
| 0.2 | 0.2 | 0.007890 |
| 0.3 | 0.3 | 0.035503 |
| 0.4 | 0.4 | 0.122288 |
| 0.5 | 0.5 | 0.248521 |
| 0.6 | 0.6 | 0.368836 |
| 0.7 | 0.7 | 0.481262 |
| 0.8 | 0.8 | 0.613412 |
| 0.9 | 0.9 | 1.000000 |
| 1.0 | 1.0 | NaN |

## Results DataFrame¶

In [71]:

```
display(results.to_frame("value"))
results.to_frame("value").to_csv("./results/microbiotyping/records/results1.tsv", sep="\t")
```

|  | value |
| --- | --- |
| variable |  |
| paper | microbiotyping |
| sklearn\_version | 0.20.1 |
| skbio\_version | 0.5.3 |
| n | 410 |
| n\_before\_rarefaction | 532 |
| n\_Control | 139 |
| n\_CRSsNP | 99 |
| n\_CRSwNP | 172 |
| PC1\_proportion\_explained | 36.67 |
| PC2\_proportion\_explained | 21.93 |
| PC3\_proportion\_explained | 9.16 |
| weighted\_unifrac\_PC1\_proportion\_explained | 40.16 |
| weighted\_unifrac\_PC2\_proportion\_explained | 25.67 |
| weighted\_unifrac\_PC3\_proportion\_explained | 6.83 |
| silhouette\_score\_k\_3 | 0.6 |
| silhouette\_score\_k\_4 | 0.61 |
| mra\_coryne\_microbiotype\_1 | 75.29 |
| mra\_staph\_microbiotype\_2 | 74.96 |
| mra\_staph\_aureus\_microbiotype\_2 | 47.81 |
| mra\_staph\_aureus\_microbiotype\_1 | 1.36 |
| mra\_staph\_aureus\_microbiotype\_3 | 0.3 |
| sparcc\_corr\_staph\_aureus\_coryne | -0.339 |
| sparcc\_pval\_staph\_aureus\_coryne | 0.001 |
| sparcc\_corr\_staph\_epi\_coryne | 0.271 |
| sparcc\_pval\_staph\_epi\_coryne | 0.001 |
| sparcc\_corr\_staph\_epi\_staph\_aureus | -0.269 |
| sparcc\_pval\_staph\_epi\_staph\_aureus | 0.001 |
| n\_microbiotype\_3S | 21 |
| n\_microbiotype\_3H | 16 |
| n\_microbiotype\_3M | 9 |
| n\_microbiotype\_3P | 7 |
| n\_microbiotype\_3O | 18 |
| microbiotype\_3\_genera\_mra\_range\_min | 73.49 |
| microbiotype\_3\_genera\_mra\_range\_max | 95.5 |
| biplot\_n\_taxa\_arrows | 5 |
| n\_microbiotype1 | 222 |
| n\_microbiotype2 | 117 |
| n\_microbiotype3 | 71 |
| n\_microbiotype1\_percent | 54.1 |
| n\_microbiotype2\_percent | 28.5 |
| n\_microbiotype3\_percent | 17.3 |
| aspirin\_chisq\_pval | 0.02 |
| aspirin\_chisq\_pval\_corrected | 0.077 |
| continent\_chisq\_pval\_corrected | 0.001 |
| n\_dataset2\_before\_rarefaction | 129 |
| n\_dataset2 | 97 |
| n\_dataset2\_CRSsNP | 33 |
| n\_dataset2\_CRSwNP | 35 |
| n\_dataset2\_Control | 29 |
| n\_dataset2\_microbiotype1\_percent | 39.2 |
| n\_dataset2\_microbiotype2\_percent | 26.8 |
| n\_dataset2\_microbiotype3\_percent | 34 |
| n\_combined\_datasets | 507 |
| n\_differently\_classified\_samples | 3 |
| percent\_differently\_classified\_samples | 3.09 |
| percent\_samples\_berger\_parker\_50\_or\_less | 24.9 |
| percent\_samples\_berger\_parker\_greater\_than\_50 | 75.1 |
| percent\_samples\_berger\_parker\_greater\_than\_70 | 51.9 |
