# Supplementary material for: Microbiotyping the Sinonasal Microbiome
Source: Front Cell Infect Microbiol. 2020 Apr 8;10:137. doi: 10.3389/fcimb.2020.00137 (PMC7156599; doi:10.3389/fcimb.2020.00137)
Supplement: Supplementary file 2 [file Data_Sheet_1.docx]

## Supplementary Tables

Table S1A: Predominant taxa of microbiotype 1.

| genus | Mean Relative Abundance (%) | Prevalence (%) |
| --- | --- | --- |
| Corynebacterium | 75.29 | 100 |
| Staphylococcus | 10.69 | 76.58 |
| Alloiococcus | 2.79 | 28.83 |
| Moraxella | 2.31 | 9.91 |
| unidentified (Enterobacteriaceae) | 1.41 | 15.32 |
| unidentified (Neisseriaceae) | 1.18 | 20.72 |
| Streptococcus | 1 | 21.62 |
| Haemophilus | 0.56 | 9.91 |
| unidentified (Moraxellaceae) | 0.44 | 2.7 |
| Ralstonia | 0.34 | 10.36 |

Table S1B: Predominant taxa of microbiotype 2.

| genus | Mean Relative Abundance (%) | Prevalence (%) |
| --- | --- | --- |
| Staphylococcus | 74.96 | 100 |
| Corynebacterium | 9.87 | 64.1 |
| Streptococcus | 3.22 | 25.64 |
| unidentified (Enterobacteriaceae) | 1.82 | 15.38 |
| Haemophilus | 1.41 | 10.26 |
| Moraxella | 1.27 | 5.13 |
| Ralstonia | 1.19 | 11.97 |
| Pseudomonas | 1.05 | 6.84 |
| Parvimonas | 0.72 | 0.85 |
| unidentified (Neisseriaceae) | 0.61 | 7.69 |

Table S1C: Predominant taxa of microbiotype 3.

| genus | Mean Relative Abundance (%) | Prevalence (%) |
| --- | --- | --- |
| Haemophilus | 23.78 | 40.85 |
| Streptococcus | 23.22 | 46.48 |
| Moraxella | 12.11 | 19.72 |
| Pseudomonas | 9.17 | 15.49 |
| unidentified (Enterobacteriaceae) | 5.74 | 9.86 |
| Serratia | 5.7 | 8.45 |
| Klebsiella | 2.75 | 4.23 |
| Corynebacterium | 2.56 | 46.48 |
| Prevotella | 1.44 | 12.68 |
| Acinetobacter | 1.38 | 1.41 |

Table S2: Addressing previous criticism to gut enterotyping.

| Critique | Answer |
| --- | --- |
| Discrete clusters or a multi-dimensional gradient? | We acknowledge the a proportion of samples fall in the gradient between the proposed microbiotypes. Berger-Parker index investigation showed that most samples had one dominating taxon. |
| Do discrete clusters link to human disease? | No. We report that we could not find an association between the microbiotype and chronic sinusitis disease status. |
| Is sampling frame or selection bias affecting results? | No; Multi-centre international study with consecutive sampling methodology. We also validate on a separate dataset. |
| Use inappropriate visualization such as “star-burst plots”? | We did not use inappropriate visualizations. |
| Use a supervized approach “between-class analysis”? | We use an unsupervised clustering and dimensionality reduction approach. |
| Is an individual’s microbiotype stable over time? | Answer unknown; Future longitudinal studies required. |

## Supplementary Figures


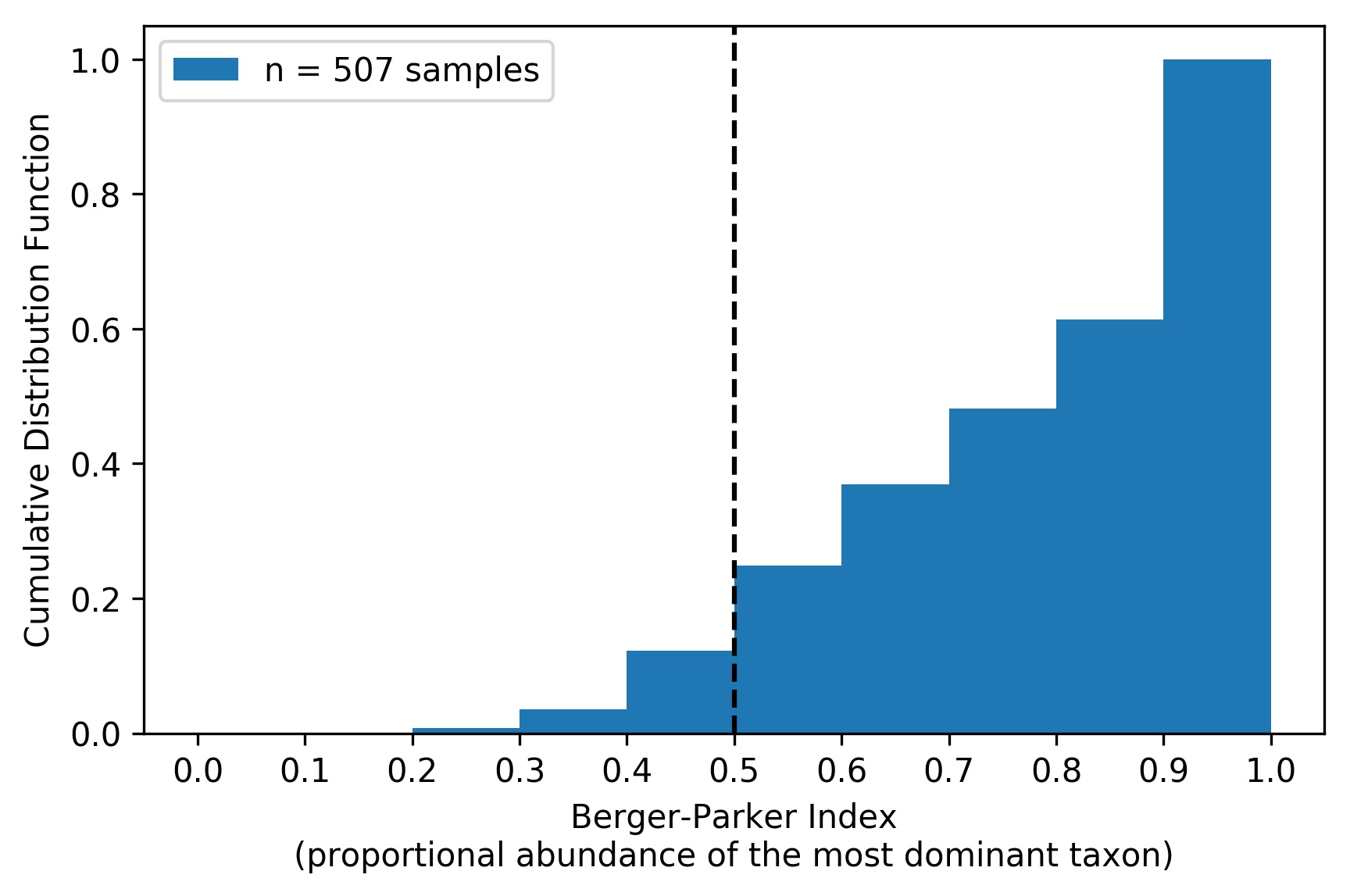


Figure S1: Cumulative distribution function of the Berger-Parker Index in the combined datasets.
